# Molecular mutagenesis of ppGpp: turning a RelA activator into an inhibitor

**DOI:** 10.1038/srep41839

**Published:** 2017-02-03

**Authors:** Jelena Beljantseva, Pavel Kudrin, Steffi Jimmy, Marcel Ehn, Radek Pohl, Vallo Varik, Yuzuru Tozawa, Victoria Shingler, Tanel Tenson, Dominik Rejman, Vasili Hauryliuk

**Affiliations:** 1 University of Tartu, Institute of Technology, Nooruse 1, 50411 Tartu, Estonia; 2 Department of Molecular Biology, Umeå University, Building 6K, 6L University Hospital Area, SE-901 87 Umeå, Sweden; 3 Laboratory for Molecular Infection Medicine Sweden (MIMS), Umeå University, Building 6K and 6L, University Hospital Area, SE-901 87 Umeå, Sweden; 4 Institute of Organic Chemistry and Biochemistry, Czech Academy of Sciences v.v.i., Flemingovo nám. 2, 166 10 Prague 6, Czech Republic; 5 Graduate School of Science and Engineering, Saitama University, 255 Shimo-Okubo, Sakura-ku, Saitama, Saitama 338-8570, Japan

## Abstract

The alarmone nucleotide (p)ppGpp is a key regulator of bacterial metabolism, growth, stress tolerance and virulence, making (p)ppGpp-mediated signaling a promising target for development of antibacterials. Although ppGpp itself is an activator of the ribosome-associated ppGpp synthetase RelA, several ppGpp mimics have been developed as RelA inhibitors. However promising, the currently available ppGpp mimics are relatively inefficient, with IC_50_ in the sub-mM range. In an attempt to identify a potent and specific inhibitor of RelA capable of abrogating (p)ppGpp production in live bacterial cells, we have tested a targeted nucleotide library using a biochemical test system comprised of purified *Escherichia coli* components. While none of the compounds fulfilled this aim, the screen has yielded several potentially useful molecular tools for biochemical and structural work.

Bacteria employ an array of systems to sense their environment and respond to various stimuli. One of such systems is mediated via changes in the intracellular levels of alarmone nucleotides guanosine tetraphosphate (ppGpp) and pentaphosphate (pppGpp), collectively referred to as (p)ppGpp^1^,^2^. The nucleotides are synthesized by RelA/SpoT Homologue (RSH) enzymes^3^ via an in-line nucleophilic attack of the 3′-OH group of GDP (or GTP) on the β-phosphate of ATP^4^ (Fig. 1a). (p)ppGpp is a pleotropic intracellular effector targeting numerous unrelated molecular targets. It regulates transcription via direct interaction with two allosteric sites of *Escherichia coli* RNAP^5^,^6^,^7^; suppresses translation via binding to the GTP-binding pocket of ribosome-associated GTPases^8^,^9^,^10^, DNA replication via binding to the active site of DNA-dependent RNA polymerase primase DnaG^11^,^12^, and nucleotide biosynthesis via direct competition with nucleotide substrates of several enzymes involved in synthesis of GTP^13^ and ATP^14^. In addition, (p)ppGpp activates its own production via interaction with ribosome-dependent *E. coli* RSH RelA^15^.

An acute increase in (p)ppGpp concentration – referred to as ‘the stringent response’ – orchestrates a survival program leading to increased virulence and antibiotic tolerance^16^. In *E. coli*, the stringent response induced by amino acid limitation is mediated by ribosome-associated RSH RelA which is strongly activated by the presence of deacylated tRNA in the ribosomal A-site^17^. Due to the central role of the (p)ppGpp in regulation of bacterial virulence^16^ and recently proposed connection to formation of antibiotic-tolerant persister cells^18^, (p)ppGpp-mediated signaling constitutes a promising target for development of novel antibacterials.

To date two approaches have been employed for the development of chemical tools to inhibit cellular (p)ppGpp production. First, synthetic cationic peptide 1018 and its derivatives were suggested to bind to (p)ppGpp directly and mark the nucleotide for degradation^19^,^20^. The 1018 peptide has a very pleotropic effect on cell physiology: in addition to targeting bacterial biofilm formation, it regulates innate immunity via modulation of macrophage differentiation and suppresses inflammation by attenuating pro-inflammatory cytokine production (reviewed in Mansour *et al*.^21^). Follow up studies have shown that, however promising as an antibacterial, 1018 is not a specific inhibitor of the stringent response^22^,^23^.

The second approach has targeted RSH enzymes directly using ppGpp-based synthetic inhibitors^24^,^25^,^26^. Redesigning the ppGpp scaffold for inhibition of intracellular RSH enzymes poses several problems. First, the molecule has to be more ‘drug-like’, i.e. less charged, more hydrophobic, and, preferably, simpler and smaller. Even though known antibiotics do not follow Lipinski’s ‘rule of five’ – they are larger, have more H-acceptor and H-donor groups and less hydrophobic (especially in the case of compounds targeting Gram-negative bacteria) than drugs in general^27^ – ppGpp is still a clear outlier when it comes to hydrophobicity: it has a calculated distribution-coefficient at pH 7.4, clogD_7.4_, of −13.87, which is more than ten clogD units lower than that of antibacterials on average. Second, in order to survive in the intracellular milieu, the molecule should be made considerably more resistant to chemical and enzymatic degradation. Third, conformational flexibility of 3′ and 5′ pyrophosphate moieties of ppGpp is critical for its interaction with target proteins^28^ imparting additional structural constraints on design of derivatives.

The ‘Wexselblatt’s bisphosphonate’ – or (**10**) – was the first step towards achieving these goals. It is considerably more chemically stable than ppGpp due to replacement of the oxygen atoms connecting the phosphate groups with methylene bridges (Fig. 1b)^26^. However, the compound is relatively inefficient, requiring 1 mM concentration for 50% inhibition of *E. coli* RelA in the test tube, and is extremely hydrophilic (predicted theoretical clogD_7.4_ = −3.18 ± 0.85 using ACD/Labs package), rendering it inactive against live bacteria. The second-generation inhibitor Relacin is a more dramatic modification of the ppGpp scaffold: the pyrophosphate groups are replaced by diglycine moieties and the guanine base has a 2-*N*-isobutyryl (iBu) protecting group attached to the exocyclic amino group at C-2 position (Fig. 1c)^24^,^25^. The resultant compound is significantly less hydrophilic than ppGpp (theoretical clogD_7.4_ = −7.95 ± 1.03), and at mM-range concentrations has a biological effect on Gram-positive bacterium *Bacillus subtilis*^26^.

However promising, the ppGpp-analogues developed to date are still far from entering the drug development pipeline due to their low potency, requiring concentrations of ≈1 mM to achieve significant inhibition of RSH enzymes^24^,^25^. Therefore, we have undertaken a targeted screen for more potent nucleotide-based RSH inhibitors using our biochemical *in vitro* system comprised of purified *E. coli* components^15^.

## Results

For the initial characterization of compounds, we followed the inhibition of [^3^H] GDP conversion to [^3^H] ppGpp catalyzed by *E. coli* RelA in a simplified system in which RelA’s activity was induced by vacant 70S ribosomes and 100 μM of ppGpp^15^. Unlabeled ppGpp was added to reaction mixtures in order to linearize the kinetics of [^3^H] ppGpp synthesis due to an activating effect on the RelA enzyme^15^. We used a targeted library of 69 nucleotides belonging to several structural classes: ‘true’ ppGpp analogues; Relacin and its derivatives; pyrrolidine, azetidine, piperidine and acyclic phosphonates. Chemical structures of tested compounds and titrations in the RelA:70S:ppGpp system are presented in Supplementary Table 1.

### A targeted screen for nucleotide-based RelA inhibitors

#### RSH inhibitors based on the ppGpp molecular scaffold

This class of compounds is unlikely to yield RSH inhibitors active against live bacteria since the exceedingly hydrophilic ppGpp scaffold is likely to compromise the pharmacokinetic properties. Nevertheless, a potent and specific ppGpp-based RSH inhibitor that acts in the test tube is useful, since it could i) serve as molecular tool for biochemical and structural studies and ii) be used to generate Structure-Activity Relationship (SAR) data instructive for development of inhibitors based on other molecular scaffolds. As a reference, we characterized ppGpp itself (Fig. 2a). In agreement with our earlier observations^15^, up to 100 μM of ppGpp activates RelA’s enzymatic activity, while at higher concentrations ppGpp acts as a weak inhibitor of RelA with an IC_50_ of 0.72 ± 0.44 mM.

**Figure 1:**
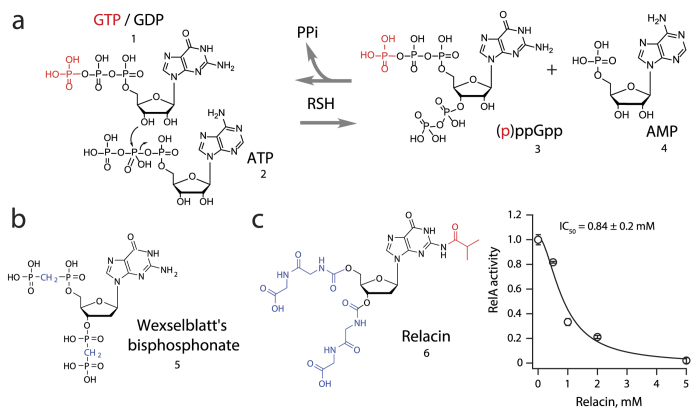
(p)ppGpp synthesis and degradation by RelA-SpoT Homologue (RSH) enzymes and design of RSH inhibitors based on the ppGpp scaffold. (**a**) RSH enzymes synthesize (p)ppGpp using ATP and GTP/GDP as substrates. Hydrolysis of (p)ppGpp regenerates GTP/GDP, accompanied by release of pyrophosphate (PPi). (**b**) Structure of the first-generation ppGpp-based RSH inhibitor 2′-deoxyguanosine-3′-5′-di(methylene bisphosphonate) or (**10**)^26^. (**c**) Structure of the second-generation ppGpp-based RSH inhibitor Relacin^25^ and its efficiency in inhibition of *E. coli* RelA in *in vitro* system from purified components^15^. N^2^-isobutyryl-guanine (G^iBu^) base modification is highlighted in red. The reaction mixture contained 30 nM RelA, 0.5 μM 70S, 100 μM ppGpp, 0.3 mM [^3^H]GDP and 1 mM ATP. RelA enzymatic activity (turnover, ppGpp synthesized per RelA per minute) is normalized to that in the absence of an inhibitor. Error bars represent standard deviations of linear regression estimates, each experiment was performed at least three times.

**Figure 2:**
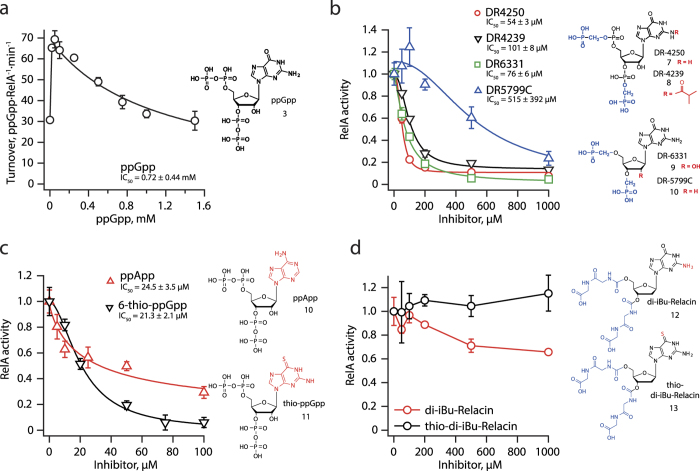
Inhibition of *E. coli* RelA by ppGpp, ppGpp-based compounds and Relacin derivatives. The reaction mixture contained 30 nM RelA, 0.5 μM 70 S, 100 μM ppGpp, 0.3 mM [^3^H]GDP and 1 mM ATP. RelA enzymatic activity (turnover, ppGpp synthesized per RelA per minute) is normalized to that in the absence of an inhibitor. Error bars represent standard deviations of linear regression estimates, each experiment was performed at least three times. (**a**) ppGpp activates RelA at low concentrations (<50 μM) and acts as a weak inhibitor at higher concentrations. (**b**) Addition of N^2^-isobutyryl guanine base modification present in Relacin (highlighted in red) to DR-4250 yielding DR4239 does not dramatically alter the activity. Removal of 2′ hydroxyl group from DR-4250 yielding DR-6241A (Supplementary Table 1) and bis (phosphonoacyl) analogue DR-6331 yielding DR-5799C significantly decreases the activity. (**c**) Substitution of the base in ppGpp for adenine yielding ppApp or 6-thio-guanine yielding thio-ppGpp results in a dramatic increase in the efficiency of RelA inhibition (**d**) SAR elements characteristic for RelA inhibition by ppGpp-based inhibitors (**b,c**) are not transferable to Relacin scaffold: removal of the N^2^-isobutyryl protective group yielding di-iBu-Relacin compromises its activity against RelA and substitution of guanine for 6-thio-guanine yielding thio-di-iBu-Relacin leads to a complete inactivation.

As a first step, we tested several modifications of the phosphate moieties of the scaffold. While several variants of non-hydrolysable ppGpp mimics relying on the modifications of the pyrophosphate moieties have been reported^26^, these compounds are relatively inefficient. The most potent representative, 2′-deoxyguanosine-3′-5′-di(methylene bisphosphonate) or (**10**), has an IC_50_ of ≈1 mM^26^ (Fig. 1b). In an attempt to improve the efficiency, we synthesized a set of derivatives in which the pyrophosphate moieties of ppGpp were replaced with phosphonomethyloxy (PCH_2_O-), phosphonoacetyl (PCH_2_CO-), phosphonopropionyl (PCH_2_CH_2_CO-), phosphonomethylaminocarbonyl (PCH_2_NHCO-), and phosphonomethyloxyphosphate (PCH_2_OPO-) groups (Supplementary Table 1). The most efficient inhibitor from this set is DR-4250 (IC_50_^DR-4250^ = 54 ± 3 μM) (Fig. 2b). The compound differs from ppGpp by the presence of methylene bridges (-CH_2_) adjoining the β-phosphorus atom and pyrophosphate bridging oxygen atom (PCOP). While structurally very similar to (**10**) in which methylene bridges replace the bridging oxygen atom, DR-4250 is an order of magnitude more potent inhibitor of RelA. A 2′-deoxy derivative DR-6241A has reduced activity (IC_50_^DR-6241A^ = 0.47 ± 0.18 mM) and further removal of 3′-pyrophosphate moiety yields even less potent DR-6222 (IC_50_^DR-6222^ = 0.73 ± 0.06 mM) (Supplementary Table 1). In order to reduce the net charge of DR-4250 we removed both phosphate groups generating bis (phosphonomethyl) derivative DR-6331. With an IC_50_ of 76 ± 6 μM the compound is, surprisingly, nearly as active as parental DR-4250 (Fig. 2b). From the medicinal chemistry point of view the structure of DR-6331 is promising for further derivatization because of first, chemical and enzymatic stability due to absence of pyrophosphate or phosphate ester functions and, second, possibility of conversion to a prodrug form with masked negative charges. Similarly to DR-4250, removal of the 2′ OH group of DR6331 yields a significantly less active 2′-deoxyguanosine derivative DR-5799C with IC_50_ of 515 ± 392 μM (Fig. 2b), underscoring the functional importance of 2′ hydroxyl group. We have synthesized and tested five additional bis (phosphonoacyl) analogues; however, none of these compounds are active against RelA (Supplementary Table 1).

We next tested several ppGpp analogues containing a modified nucleotide base. The molecular mechanism by which ppGpp activates RelA’s synthetic activity is unclear^15^. To test the specificity of the effect, we synthesized an adenine derivative of ppGpp, ppApp. The 6-thioguanosine derivative of ppGpp, 6-thio-ppGpp, is a UV-inducible zero-length crosslinking reagent that was successfully used to map the two ppGpp binding sites of *E. coli* RNAP^6^,^29^, suggesting that a similar approach could potentially be used to map the ppGpp binding site of RelA. Surprisingly, unlike ppGpp, neither ppApp nor 6-thio-ppGpp activate RelA’s synthetic activity (Supplementary Figure 1). On the contrary, both compounds are potent inhibitors with IC_50_ of 24.5 ± 3.5 μM and 21.3 ± 2.1 μM, respectively (Fig. 2c). The only nucleotide-based RSH inhibitor that showed activity against bacterial cultures, Relacin, has N^2^-isobutyryl-guanine (G^iBu^) modification of the nucleotide base^25^. This modification is a common protective group used in nucleotide chemistry^30^. The original publication did not explain the rationale behind using this modification – Is it important for the SAR of the inhibitor? Is it merely a result of the omission of the deprotection stage due to technical difficulties? – Therefore, we synthesized and tested several G^iBu^-modified compounds and found that replacement of the G base in DR-4250 with G^iBu^ slightly decreases its activity (IC_50_^DR-4239^ = 101 ± 8 μM) (Fig. 2b).

Finally, we attempted to apply the structural alterations listed above to Relacin. As an initial step we tested Relacin itself. In good agreement with earlier estimates^25^,^31^, the compound is relatively inefficient with IC_50_ of 0.84 ± 0.2 mM (Fig. 1c). Next, we tested the effect of the removal of the iBu protection group from guanine residue of Relacin. This resulted in a near-complete inactivation of the compound (Fig. 2d), demonstrating that iBu is crucial for Relacin’s activity against RelA. When we replaced the G^iBu^ in the Relacin scaffold with 6-thio-G, the resulting molecule DR-5732 had virtually no activity (Fig. 2d).

#### Guanosine phosphonates

The sugar-phosphate moiety of ‘true’ ppGpp-based RSH inhibitors poses a significant hurdle for medicinal chemistry because it is large, complex, and highly charged. Therefore we attempted to develop a simpler and less charged nucleotide phosphonate structural backbone as a platform for future derivatization. This class of compounds has generated efficient inhibitors of various classes of evolutionary unrelated enzymes such as viral DNA polymerases^32^ and malarial hypoxanthine-guanine-xanthine phosphoribosyltransferase^33^.

**Figure 3:**
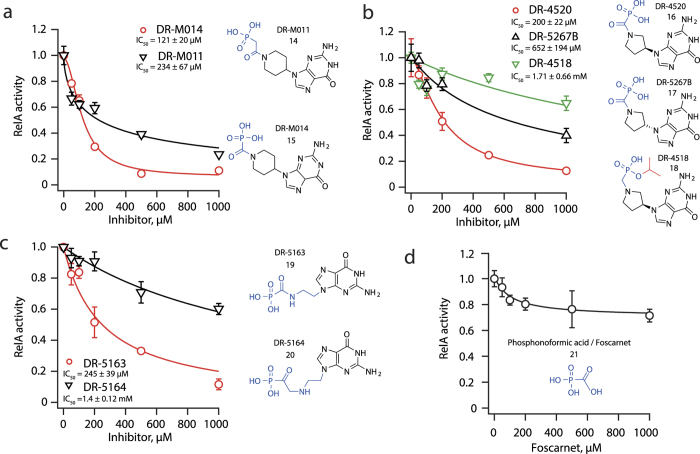
Inhibition of *E. coli* RelA by guanosine phosphonates. The reaction mixture contained 30 nM RelA, 0.5 μM 70 S, 100 μM ppGpp, 0.3 mM [^3^H]GDP and 1 mM ATP. RelA enzymatic activity (turnover, ppGpp synthesized per RelA per minute) is normalized to that in the absence of an inhibitor. Error bars represent standard deviations of linear regression estimates, each experiment was performed at least three times. (**a**) RelA inhibition by piperidine phosphonate DR-M014 containing phosphonocarbonyl group and DR-M011 containing phosphonoacetyl. (**b**) Inversion of the stereocenter in the C-3 position of the pyrrolidine ring of pyrrolidine phosphonate DR-4520 (yielding DR-5267B) or mono-isopropylester modification of the phosphonocarbonyl group (yielding DR-4518) decreases its activity. (**c**) Substitution of phosphonocarbonyl group in acyclic phosphonates DR-5163 by phosphonoacetyl yielding in DR-5164 results in a significant loss of activity against RelA. (**d**) Phosphonoformate or Foscarnet^39^ does not inhibit RelA despite the presence of a phosphonocarbonyl moiety.

We assembled and tested a targeted library of structurally diverse guanine nucleoside phosphonates (see Supplementary Table 1)^34^,^35^,^36^,^37^,^38^. In this compound series – piperidine, pyrrolidine, prolinol, azetidine and acyclic phosphonates – the size of the heterocyclic amine ring is progressively smaller, decreasing from a six-membered ring to a linear molecule. Out of the 12 piperidine phosphonates, the most potent inhibitor is DR-M014 with an IC_50_ of 121 ± 20 μM (Fig. 3a). Replacement of the phosphonocarbonyl group for phosphonoacetyl resulted in a less efficient compound DR-M011 (IC_50_ = 234 ± 67 μM). Out of 21 pyrrolidine phosphonates tested, the most potent inhibitor is DR-4520 with an IC_50_ of 200 ± 22 μM, which is almost twice less efficient than the most potent piperidine phosphonate, DR-M014 (Fig. 3b). An enantiomeric pyrrolidine phosphonate DR-5267B (*R*), which differs from DR-4520 (an 3-C *S* isomer) by the configuration of the 3-C carbon atom of the pyrrolidine ring, is significantly less active (IC_50_^DR-5267B^ = 652 ± 194 μM vs IC_50_^DR-4520^ = 200 ± 22 μM), pointing towards the specificity of inhibition. As was the case for DR-M014, the phosphonocarbonyl group is important for the activity of DR-4520: a mono-isopropylester modification results in a near-inactive compound DR-4518 (IC_50_ = 1.71 ± 0.66 mM) (Fig. 3b). Modification of the pyrrolidine ring by the addition of hydroxymethyl group at C-2 to afford 2-pyrrolidinemethanol (prolinol) resulted in inactivation of the compound (Supplementary Table 1). An acyclic scaffold is highly advantageous from the synthetic chemistry viewpoint due to the absence of stereoisomeric centers, thus eliminating the need for stereospecific synthesis. Out of three tested acyclic phosphonates, the most active representative – DR-5163 – inhibits RelA in our biochemical system with an IC_50_ of 245 ± 39 μM (Fig. 3c). Replacement of the phosphonocarbonyl group in DR-5163 by phosphonoacetyl results in a dramatically less active compound DR-5164 (IC_50_^DR-5164^ = 1.4 ± 0.12 mM) (Fig. 3c).

The presence of a phosphonocarbonyl moiety is a recurring feature of the active phosphonate inhibitors tested so far. Hydrolysis of the amide bond would result in the formation of phosphonoformic acid. This compound, marketed as Foscarnet, is a well-characterized inhibitor of viral DNA polymerase by acting as a substrate analog mimicking the pyrophosphate moiety of NTP^39^. Because both the substrates and products of RSH enzymes contain pyrophosphate, it is likely that the same mechanism is at play in the case of active phosphonates such as DR-M014, DR-4520 and DR-5163. We tested the effect of phosphonoformic acid in our system, and by itself, it has virtually no inhibitory activity at concentrations up to 1 mM (Fig. 3d).

#### Naturally occurring nucleotides

There are several examples of direct cross-talk between bacterial nucleotide-based signaling systems, connecting ppGpp and c-di-AMP^40^, and cyclic GMP and cyclic di-GMP^41^ regulatory networks. Therefore, we tested a set of common signaling nucleotides and nucleotide cofactors: c-di-AMP, c-di-GMP, c-di-GAMP, NADH, NADPH. None of the compounds showed any inhibitory effect on *E. coli* RelA in concentration up to 1 mM (Supplementary Table 1).

### Characterization of the promising RSH inhibitors

For the analysis of the mechanism of action, we selected the ‘true’ ppGpp analogue DR-4250, the most potent piperidine phosphonate DR-M014, and the most extensively characterized RSH inhibitor to date, Relacin^25^,^31^.

#### Inhibition of RelA activated by programmed ‘starved’ ribosomal complexes

**Figure 4:**
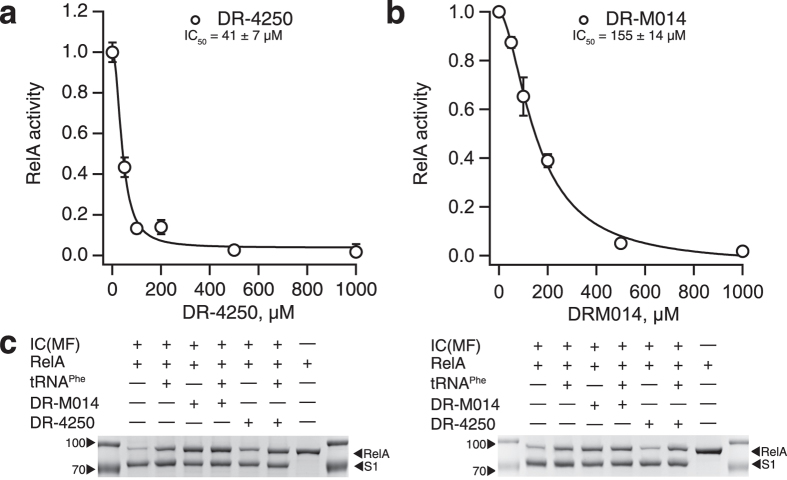
Functional studies of DR-M014 and DR-4250 with programmed ribosomal complexes. (**a**,**b**) Inhibition of RelA activated by starved ribosomal complexes containing deacylated A-site tRNA^Phe^. The reaction mixture contained 30 nM RelA, 0.5 μM 70S, 2 μM tRNA^Phe^, 2 μM tRNA_i_^Met^, 2 μM mRNA(MF), 100 μM ppGpp, 0.3 mM [^3^H] GDP and 1 mM ATP. The experiments were performed in HEPES:Polymix buffer with 5 mM Mg^2+^. Error bars represent standard deviations of linear regression estimates, each experiment was performed at least three times. (**c**) A-site tRNA^Phe^ and nucleotides DR-M014 and DR-4250 promote RelA binding to 70S initiation complexes. The panel shows two independent experimental replicates. Reaction mixture containing combinations of 2 μM RelA, 1 μM 70S initiation complexes programmed with mRNA(MF), 3 μM deacylated tRNA^Phe^ and test compounds at 500 μM was preincubated at 37 °C for 15 minutes prior to loading 50 μl samples on top of a 50 μl 30% sucrose cushion. After centrifugation for 25 minutes (70,000 r.p.m. at 12 °C) the supernatants were quickly aspirated, the pellets resuspended in 20 μl of SDS loading buffer, and the proteins resolved on 10% SDS-PAGE gel.

Screening was performed in a cost-efficient way using RelA activated by the presence of vacant 70S ribosomes and 100 μM ppGpp. However, to become fully active, RelA requires the presence of ‘starved’ ribosomal complexes containing deacylated A-site tRNA^17^. We therefore prepared ‘starved’ complexes using model mRNA coding for fMetPhe (MF) dipeptide and purified deacylated tRNAs tRNA^Phe^ and tRNA^fMet^_i_^42^ to test the efficiency of DR-4250 and DR-M014 in this more physiologically relevant system (Fig. 4a,b). DR-4250 and DR-M014 display similar efficiency in both systems (IC_50_^DR-4250, 70S^ = 54 ± 3 μM vs. IC_50_^DR-4250, 70S(MF:tPhe)^ = 41 ± 7 μM; IC_50_^DR-M014, 70S^ = 121 ± 20 vs. IC_50_^DR-M014,70S(MF:tPhe)^ = 155 ± 14 μM), suggesting a possibility for the compounds to be efficient in live cells.

The ppGpp-analogues reported earlier promote RelA association with 70S ribosome^25^,^26^ and are, therefore, promising tools for generating stable 70S:RelA complexes for structural investigations. We tested DR-4250 and DR-M014 using a modified version of a spin down assay of Agirrezabala and colleagues^43^, using initiation complexes (IC) programmed with MetPhe mRNA, (MF), in the presence and absence of deacylated A-site tRNA^Phe^, and in the presence and absence of inhibitor (Fig. 4c). In a good agreement with earlier results, the presence of A-site deacylated tRNA strongly promotes RelA binding to the ribosome^43^, resulting in a stoichiometry of RelA to ribosomal protein S1 close to unity. Addition of 500 μM DR-4250 has virtually no effect on RelA binding, while DR-M014 significantly promotes RelA binding to the IC in the absence of tRNA^Phe^.

#### DR-M014 and DR-5163 are inefficient inhibitors of *Enterococcus faecalis* SAS RelQ

In *E. coli*, ppGpp is synthesized by the multi-domain RSHs RelA and SpoT, however, numerous single-domain RSHs – Small Alarmone Synthetases (SAS) – are widely distributed across bacterial taxa^3^. We found earlier that *E. faecalis* SAS RelQ (RelQ_*Ef*_) is virtually insensitive to Relacin^31^. Therefore we tested the effects of DR-4250, DR-M014 and DR-5163 on RelQ_*Ef*_ activity (Supplementary Figure 2). While DR-M014 and DR-5163 were almost inactive at concentrations up to 1 mM, DR-4250 did inhibit RelQ_*Ef*_ (IC_50_^RelQ^ = 235 ± 19 μM), though significantly less efficient than *E. coli* RelA.

#### Off-target effects: inhibition of *E. coli* EF-G GTPase and RNA Polymerase

Since (p)ppGpp targets numerous enzymes, it is likely that a compound based on this scaffold would be a promiscuous binder as well. This promiscuity can be viewed as an advantage (e.g. because it would be harder for a bacteria to gain resistance by simultaneously altering several binding sites) or as a disadvantage (e.g. lack of strict specificity would render the inhibitor less useful as a molecular tool). Therefore, it is instrumental to test the off-target effects of the potential inhibitors.

**Figure 5:**
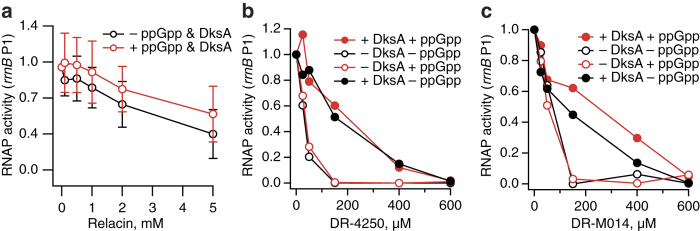
Inhibition of multiple round *in vitro* transcription reaction by Relacin (**a**), DR-4250 (**b**) and DR-M014 (**c**). Effect of inhibitors on *rrnB* P1 (−66 to +50) promoter transcription by RNAP. Graphs depicting inhibitor (0, 0.025, 0.05, 0.15, 0.4 and 0.6 mM) titrations performed at 30 °C in T-buffer with 0.5 nM template (σ^70^-*rrnB* P1 promoter (pRLG6214)) and 5 nM σ^70^-RNAP, in presence or absence of 100 μM ppGpp and/or 2 μM DksA. Error bars represent standard deviations of linear regression estimates, each experiment was performed at least three times.

Initially, we tested the effect of the most promising compounds on GTPase activity of EF-G stimulated by 70S ribosomes. We detected no inhibitory effect of Relacin (up to 5 mM), DR-4250, DR-M014, DR-5191B and DR-5163 (all up to 1 mM) or ppApp (up to 100 μM) (Supplementary Figure 3). Next, we tested their effects on RNAP. In *E. coli,* effects of ppGpp on RNAP are augmented by the transcription initiation factor DksA^44^. This small protein binds to the secondary channel of RNAP^45^,^46^ – a tunnel via which NTPs are delivered to enzyme’s active center^47^. The interaction affects transcriptional initiation via stimulation of an isomerization step in the pathway leading to open complex formation in a promoter-specific manner^44^ and increases the fidelity of transcription elongation^48^. We used multiple round *in vitro* transcription driven from the *rrnB* P1, a well-characterized ppGpp/DksA inhibited promoter, as described earlier^49^. The assays were performed in the presence and absence of 2 μM DksA and/or 100 μM ppGpp (Fig. 5a). When added at concentrations up to 5 mM, Relacin had a mild inhibitory effect, regardless of the presence and absence of DksA and ppGpp. Both DR-4250 and DR-M014 had a very different effect on RNAP (Fig. 5b,c). In the absence of DksA, irrespective of the presence or absence of ppGpp – both components were more potent inhibitors of RNAP then of RelA – and addition of DksA had a pronounced protective effect (compare Figs 4a,b and 5b,c). The intrinsically ppGpp-insensitive promoter *rna1*^44^ displayed the same behavior (Supplementary Figure 4).

#### DR-4250, DR-5163 or DR-M014 do not act on live *B. subtilis* cultures

Finally, we tested the three of our most promising inhibitors (DR-4250, DR-M014 and DR-5163) for RSH inhibition in bacterial culture. For this we used the Gram-positive bacterium *B. subtilis* due to its better uptake of compounds as compared to Gram-negative species such as *E. coli*^50^. The functional test for RSH inhibition relied on the auxotrophy of (p)ppGpp-deficient (ppGpp^0^) *B. subtilis* for methionine and branched-chain amino acids valine, leucine and isoleucine^51^,^23^. The *B. subtilis* RSH repertoire consists of one multi-domain ribosome-associated enzyme Rel and two SAS, YwaC and YjbM^3^,^52^. Because the SAS RelQ_*Ef*_ is refractory to inhibition by our test compounds (Supplementary Figure 2), we used a *B. subtilis* strain lacking SASs (Δ*ywaC*Δ*yjbM*), in which the sole source of (p)ppGpp is Rel^53^. Defined S7 medium^54^ lacking valine supports the growth of Δ*ywaC*Δ*yjbM B. subtilis*, but not the ppGpp^0^ stain (Supplementary Figure 5). Addition of increasing concentrations of DR-4250, DR-5163 or DR-M014 (up to 1 mM) does not affect the growth of Δ*ywaC*Δ*yjbM B. subtilis* in valine drop-out medium S7. This is indicative of the test compounds failing to inhibit Rel and thereby rendering the strain phenotypically ppGpp^0^ (Supplementary Figure 5). A lack of cellular uptake is a likely explanation.

## Discussion

In this project we have screened a targeted nucleotide library – a total of 69 compounds – aiming to identify a potent and specific inhibitor of RSH enzymes capable of abrogation of (p)ppGpp production in bacterial culture. While none of the compounds fulfilled this aim, the screen has yielded several potentially useful molecular tools for biochemical and structural work, as well as highlighted the role of the transcription factor DksA in RNAP fidelity. DksA binds to the secondary channel via which nucleotides enter the catalytic center of the RNAP^46^ and counteracts the misincorporation events^48^. The higher fidelity of RNAP:DksA – and, therefore, better discrimination against nucleotide compounds that can not serve as substrates – is likely to be responsible for the protective effect of DksA against DR-4250 and DR-M014 in *in vitro* transcription assays (Fig. 5).

Recent structures of *E. coli* RelA complexed with ‘starved’ ribosomal complexes are instrumental to our understanding of the protein’s mechanism on the molecular level^42^,^55^. These cryo-electron microscopy (cryo-EM) reconstructions are, however, incomplete: the highly mobile N-terminal catalytic domain is unresolved and that part of the structural model was based on earlier crystal structure of truncated Rel enzyme from *Streptococcus dysgalactiae* subsp. *equisimilis*^4^. The incompleteness of the cryo-EM structure compromises its predictive power for structure-based design of selective RelA inhibitors. ppGpp-analogues were shown to promote RelA’s association with the 70 S ribosome^25^,^26^; we see similar effects for DR-M014 and DR-4250 (Fig. 5). Therefore, this kind of compounds could assist the generation of better-resolved cryo-EM structures by both stabilizing RelA’s structure and promoting it’s binding to starved ribosomal complexes. However, one should be cautious when interpreting the structural data generated using ppGpp mimics. As we show, when interacting with *E. coli* RelA, photoreactive ppGpp analogue 6-thio-ppGpp behaves radically differently from ppGpp: the base modification converts ppGpp from an activator to potent inhibitor of RelA (Fig. 2c). 6-thio-ppGpp is an exceedingly useful molecular tool that was recently used to identify the two ppGpp binding sites of *E. coli* RNAP^6^,^29^. Importantly, both of the binding sites were confirmed by mutagenesis; validation of that kind is essential to ensure the meaningfulness of the crosslinking data. One of the most potent RelA inhibitors identified in the current study is ppApp (Fig. 2c). This nucleotide is naturally produced by a divergent SAS RSH^3^ from *Streptomyces morookaensis*^56^. Since ppApp does not have a pronounced inhibitory effect on either transcription or translation in *E. coli* reconstituted systems^57^, it has a potential as a starting point for the development of a specific RSH inhibitor.

## Methods

Detailed description of experimental procedures can be found in *Supplementary Text*. Biochemical assays utilize *in vitro* translation^58^ and stringent response^15^ systems from *E. coli* purified components. Experiments were performed in HEPES:Polymix^58^ buffer with either 5 (for enzymatically assembled initiation complexes) or 15 mM (for vacant 70 S, as well as non-enzymatically assembled programmed ribosomes) Mg^2+^. Expression and purification of *E. faecalis* RelQ was performed as per Gaca (2015) and colleagues^31^. GTPase and ppGpp synthesis were followed using TLC separation and radiochemical detection of ^3^H-labelled nucleotides^59^. Multiple round *in vitro* transcription assays were performed as per Bernardo *et al*.^49^ with minor modifications. Inhibition efficiency (IC_50_) was calculated using 4-parameter logistic model (Hill equation) as per Sebaugh^60^.

## Additional Information

**How to cite this article**: Beljantseva, J. *et al*. Molecular mutagenesis of ppGpp: turning a RelA activator into an inhibitor. *Sci. Rep.* **7**, 41839; doi: 10.1038/srep41839 (2017).

**Publisher's note:** Springer Nature remains neutral with regard to jurisdictional claims in published maps and institutional affiliations.

## Supplementary Material

Supplementary Information
