# Supplementary material for: Molecular mutagenesis of ppGpp: turning a RelA activator into an inhibitor
Source: Sci Rep. 2017 Feb 3;7:41839. doi: 10.1038/srep41839 (PMC5291098; doi:10.1038/srep41839)
Supplement: Supplementary Information [file srep41839-s1.pdf]

Supplementary Information

Molecular mutagenesis of ppGpp: turning a RelA activator into an inhibitor

Jelena Beljantseva[1,#], Pavel Kudrin[1,#], Steffi Jimmy[2,3], Marcel Ehn[4], Radek Pohl[4], Vallo Varik[1,2,3], Yuzuru Tozawa[5], Victoria Shingler[2], Tanel Tenson[1], Dominik Rejman[4,*], Vasili Hauryliuk[1,2,3,*]

[1]University of Tartu, Institute of Technology, Nooruse 1, 50411 Tartu, Estonia

[2]Department of Molecular Biology, Umeå University, Building 6K, 6L University Hospital Area, SE-901 87 Umeå, Sweden

[3]Laboratory for Molecular Infection Medicine Sweden (MIMS), Umeå University, Building 6K and 6L, University Hospital Area, SE-901 87 Umeå, Sweden

[4]Institute of Organic Chemistry and Biochemistry, Czech Academy of Sciences v.v.i., Flemingovo nám. 2, 166 10 Prague 6, Czech Republic

[5]Graduate School of Science and Engineering, Saitama University, 255 Shimo-Okubo, Sakura-ku, Saitama, Saitama 338-8570, Japan

[#] denotes equal contribution

[*] denotes the corresponding authors

Contact details of corresponding authors:

Dominik Rejman: rejman@uochb.cas.cz, +420220183371

Vasili Hauryliuk: vasili.hauryliuk@umu.se, +46907850807

Supplementary Methods

Thin layer chromatography (TLC) analysis of nucleotide mixtures

Both EF-G GTPase reaction and ppGpp synthesis by RelA were followed by TLC analysis followed by scintillation counting according to Mechold and colleagues, with modifications[1]. Time points from the reaction mixtures (5 µl, see below for details) were quenched by addition 4 µl 70% formic acid supplemented with a cold nucleotide standard used for UV-shadowing (10 mM GDP and 10 mM GTP) and spotted on PEI-TLC plates (Macherey-Nagel). TLC was run in buffer, plates dried, cut into sections guided by UV-shadowing and ^{3}H radioactivity was quantified by scintillation counting in Optisafe-3 (Fisher) scintillation cocktail on Perkin Elmer Tricarb 2810TR counter. Conversion of substrate to product was quantified as described in Shyp et al. (2012)[2].

Enzymatic assays with E. coli *RelA*

Screening: all experiments were performed in HEPES:Polymix, Mg^{2+} of 5 mM, 37 °C. The reaction mixture containing 0.1 µM RelA, 0.5 µM 70S, 300 µM ^{3}H-GDP, 100 µM ppGpp was pre-incubated at 37°C for 2 min and then reaction started by addition of 1 mM ATP, time points (5 µl) taken, quenched with formic acid and analyzed by TLC. Inhibition efficiency (IC_{50}) was calculated using 4-parameter logistic model (Hill equation) as per Sebaugh (2011)[3].

Follow-up characterization: all experiments were performed in HEPES:Polymix, Mg^{2+} of 5 mM, 37 °C. The reaction mixture containing 0.1 µM RelA, 0.5 70S, 2 µM $tRNAf^{Met}_{i}$, 2 µM $tRNA^{Phe}$, 2 µM mRNA(MF) 5'-GGCAAGGAGGUAAAAAUGUUCAAA-3', 300 µM ^{3}H-GDP, 100 µM ppGpp was pre-incubated at 37°C for 2 min and then reaction started by addition of 1 mM ATP, time points (5 µl) taken, quenched with formic acid and analyzed by TLC.

GTPase assays with E. coli *EF-G*

The reaction mixture contained 0.5 µM 70S, 0.1 µM EF-G, in HEPES:Polymix (25 mM Hepes 7.5/ 1 mM DTT / 5 mM Mg^{2+}) pre-incubated for 2 minutes at 37°C prior to addition of 300 µM [^{3}H]GTP substrate (Hartman), after which time points (5 µl) taken, quenched with formic acid and analyzed by TLC (see above).

Spin down assays

Reaction mixture containing combinations of 2 µM RelA, 1 µM 70S initiation complexes programmed with mRNA(MF), 3 µM deacylated $tRNA^{Phe}$ and test compounds at 500 µM was preincubated at 37°C for 15 minutes prior to loading 50 µl samples on top of a 50 µl 30% sucrose cushion. After centrifugation for 25 minutes (70,000 r.p.m. at 12°C) the supernatants were quickly aspirated, the pellets resuspended in 20 µl of SDS loading buffer, and the proteins resolved on 10% SDS-PAGE gel.

Multiple round in vitro *transcription assay*

The assays were performed as per Bernardo et al. (2006)[4] with minor modifications. Reactions were carried out in T-buffer (50 mM Tris-HCl pH 7.5, 100 mM NaCl, 10 mM $MgCl_2$, 1 mM dithiothreitol, 0.1 mM EDTA and 0.275 mg of BSA per ml) at 30°C. Inhibitors were titrated to final concentrations of 0, 0.025, 0.05, 0.15, 0.4 and 0.6 mM. *E. coli* σ^{70}-RNAP holoenzyme (Epicentre, final concentration 5 nM) and template plasmid DNA containing σ^{70}-rrnB P1 promoter, (pRLG6214, final concentration 0.5 nM) was used for all experiments. Before initiation of transcription, *E. coli* σ^{70}-RNAP holoenzyme and T buffer were incubated at 30°C for 5 min. Above mix was added to predispensed mixture of inhibitor and the template plasmid DNA containing σ^{70}-rrnB P1 promoter (pRLG6214), incubated for further 20 min to initiate open complex formation. 2.5 μl of a mixture of ATP (final concentration 0.5 mM), GTP and CTP (final concentration 0.2 mM each), UTP (final concentration 0.08 mM) and [α-^{32}P]-UTP (5 μCi at > 3000 Ci/mmol, PerkinElmer) was added to initiate transcription by RNAP. After 7 min, 1 μl heparin (0.125 mg/ml final concentration) was added to the reaction mixture to prevent re-initiation and further incubated for 5 min to allow completion of initiated transcripts. Transcription was terminated by addition of formamide loading buffer and samples were electrophoresed on a 7M urea-5% polyacrylamide gel and quantified by phosphorimaging.

Growth assays with Bacillus subtilis BSB1 *wild type strain*

SAS deletion and ppGpp^0 strain of *B. subtilis*[5] were grown in S7 liquid medium[6] except for amino acid requirements, which were met as described elsewhere [7]. On 96-well plate, 90 μl of *B subtilis* starter culture was mixed with 10 μL of compound dilution (in 5 mM Tris pH 7.2) so that final OD_{600} was 0.025 (*l* = 1 cm). Following incubation was without shaking at 37°C and growth was monitored with Tecan Infinite M200 at OD_{600} (raw OD_{600} values are reported). *B. subtilis* starter culture was prepared as follows. Late exponential phase cells in S7, with full set of amino acids, were diluted ≈10-fold in S7 lacking either valine or lysine so that, after addition of 8% DMSO, OD_{600} was 0.25. Next, cell suspensions were aliquoted, frozen in liquid nitrogen, and stored at -80°C. Just before the experiment, cells were thawed on ice and appropriate amount of media was added.

Supplementary figures

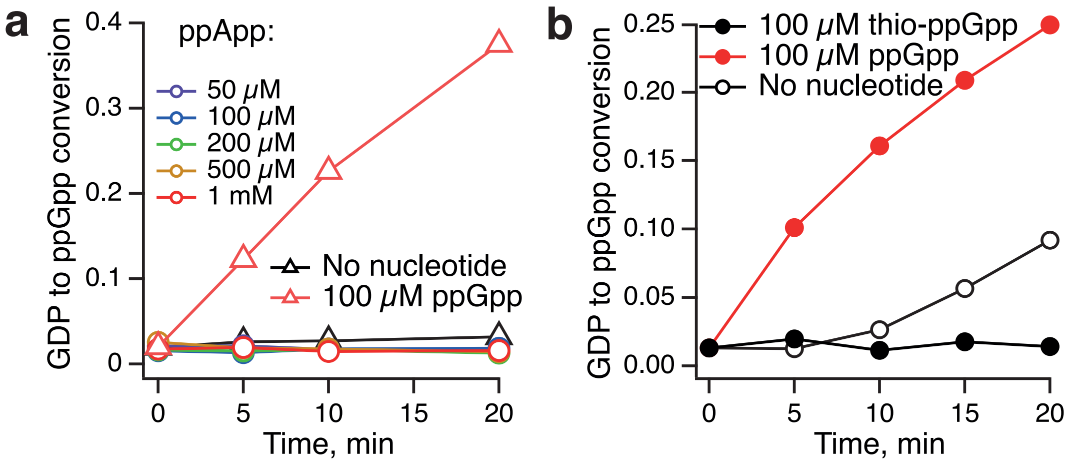

Supplementary Figure 1 | ppApp (a) and thio-ppGpp (b) do not activate RelA's ppGpp synthetic activity. The reaction mixture contained 30 nM RelA, 0.5 mM 70S, 100 μM ppGpp, 300 mM [^{3}H] GDP and 0.5 mM ATP.

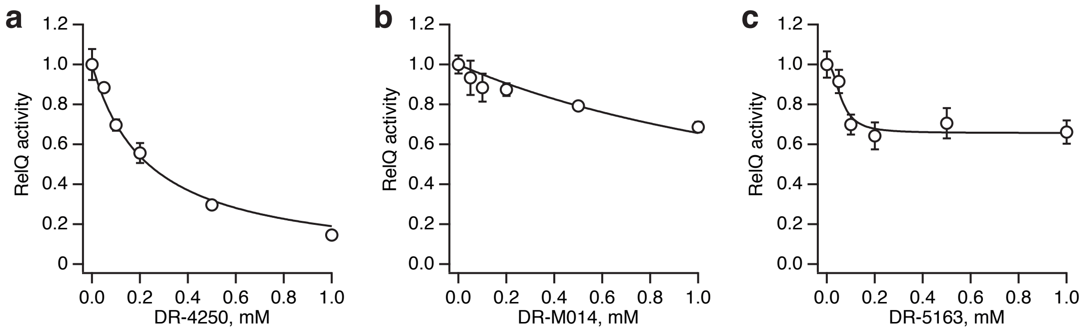

Supplementary Figure 2 | Inhibition of *E. faecalis* SAS RelQ$_{Ef}$ by DR-4250, DR-M014 and DR-5163 is inefficient. The reaction mixture contains 0.25 μM RelQ$_{Ef}$, 300 μM [^{3}H] GDP, 0.5 mg/ml BSA, 100 μM ppGpp, 1 mM ATP in Hepes:Polymix at 37°C.

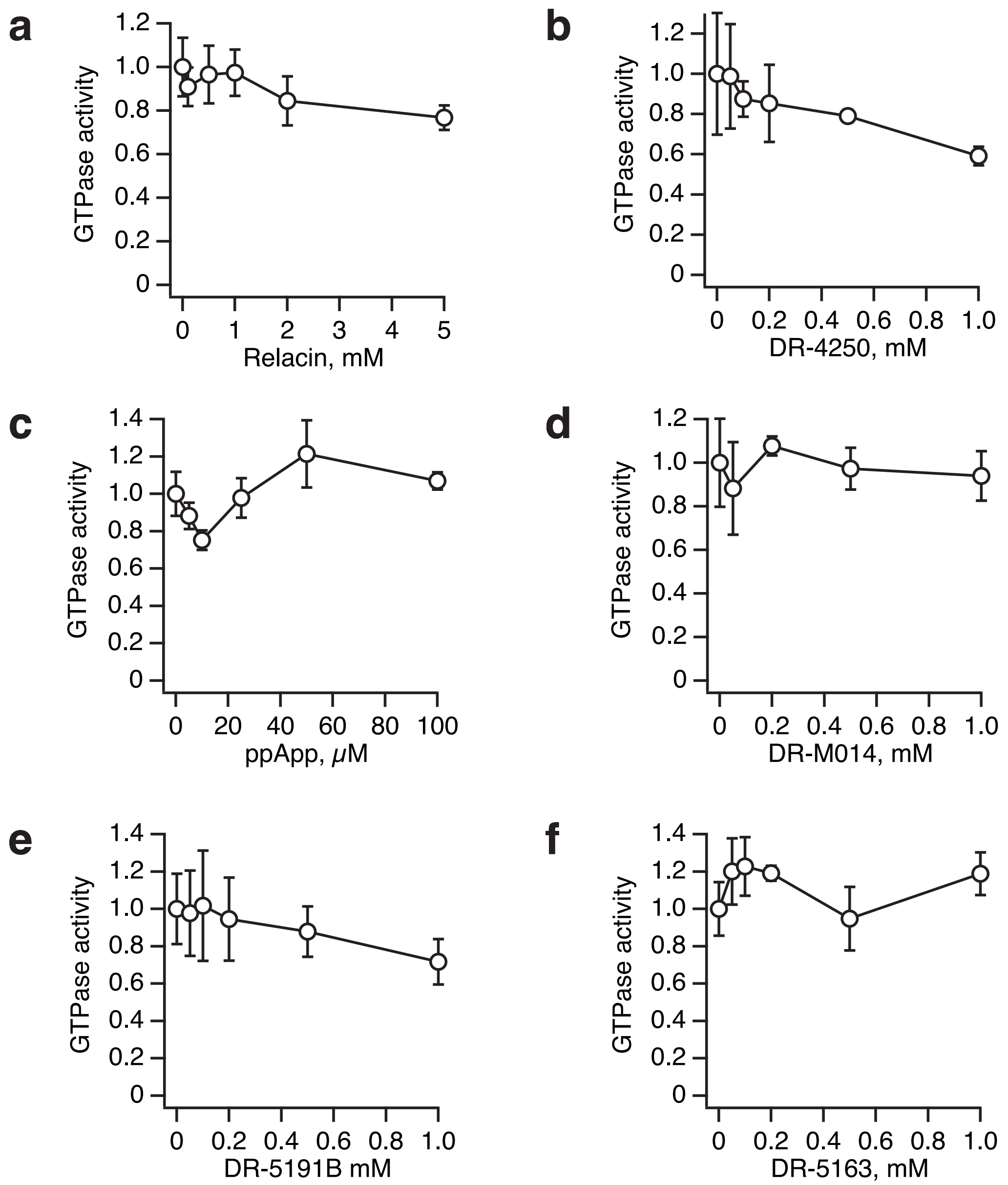

Supplementary Figure 3 | RelA inhibitors do not inhibit the GTPase activity of *E. coli* translation elongation factor EF-G. The reaction mixture contains 0.5 μM 70S, 0.1 μM EF-G, 500 μM [^{3}H] GTP in PM at 37°C as well as increasing concentrations of inhibitors: Relacin (**a**), DR-4250 (**b**), ppApp (**c**), DR-M014 (**d**), DR-5191B (**e**) and DR-5163 (**f**). Error bars represent standard deviations of linear regression estimates.

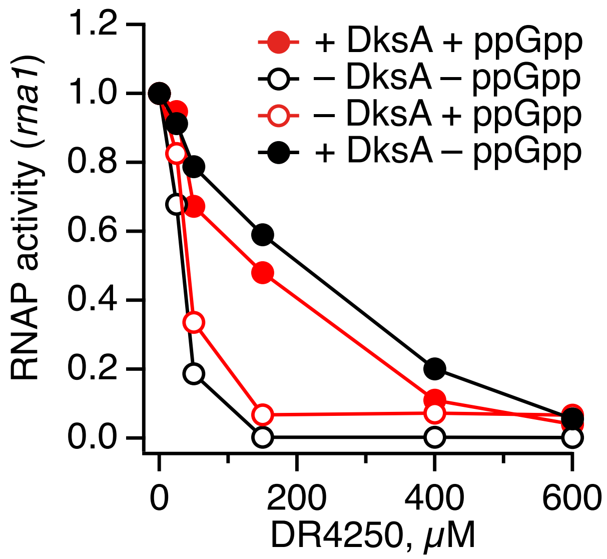

Supplementary Figure 4 | Inhibition of multiple round *in vitro* transcription reaction by DR-4250. Effect of inhibitors on *rna1* P1 promoter transcription by RNAP. Graphs depicting inhibitor (0, 0.025, 0.05, 0.15, 0.4 and 0.6 mM) titrations performed at 30°C in T-buffer with 0.5 nM template (σ^{70}-*rrnB* P1 promoter (pRLG6214)) and 5 nM σ^{70}-RNAP, in presence or absence of 100 μM ppGpp and/or 2 μM DksA.

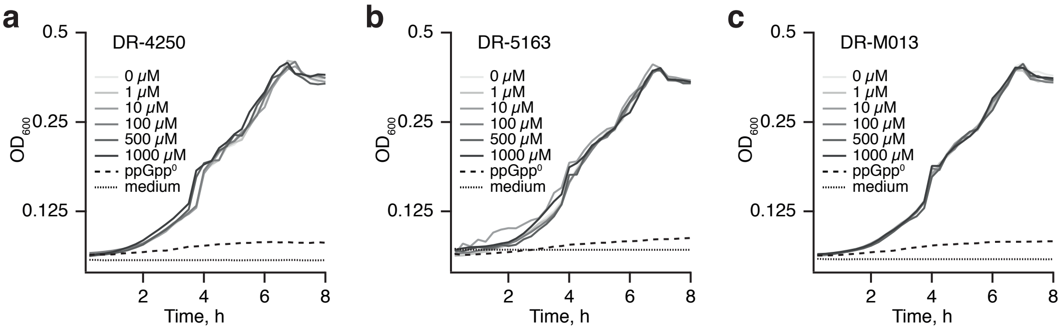

Supplementary Figure 5 | DR-4250, DR-5163 and DR-M013 do not render Δ*ywaC* Δ*yjbM* BSB1 *B. subtilis* ppGpp^{0}. BSB1 *B. subtilis* wild type strain as grown on S7 synthetic media[8] drop-out media lacking valine which abolishes the growth of ppGpp^0 (Δ*rel* Δ*ywaC* Δ*yjbM*) strain due to valine auxotrophy (dashed line). Increasing concentrations of DR-4250, DR-5163 and DR-M013 (from 0 to 1000 μM) did not affect bacterial growth suggesting that the test compounds failed to inhibit the Rel and render the strain ppGpp^0.

Supplementary Table 1 | Inhibition of *E. coli* RelA ppGpp synthesis by test compounds. [3H] GDP conversion to [3H] ppGpp catalyzed by 30 nM *E. coli* RelA activated by 0.5 µM *E. coli* vacant 70S ribosomes and 100 µM of ppGpp was followed in the presence of increasing concentrations of tested compounds. All experiments were performed in 1xHEPES:Polymix, Mg^{2+} of 5 mM, 37 °C. Error bars represent standard deviations of the turnover estimates by linear regression.

Nº	Code	Structure	Example titration
1	**ppGpp**	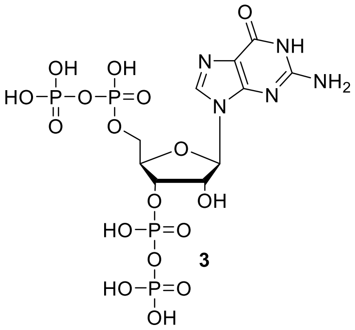 3	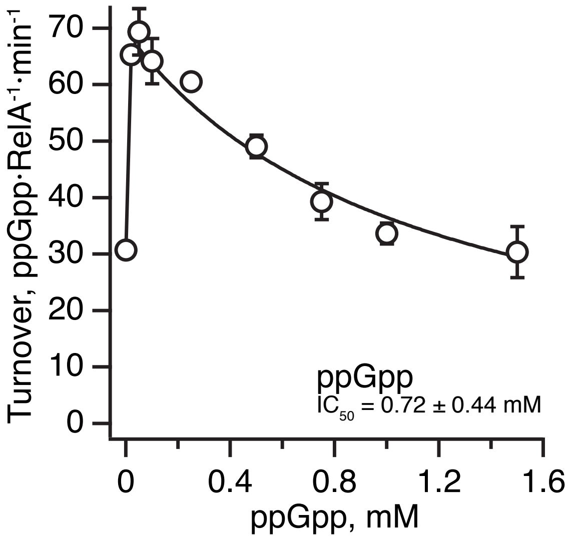
**ppGpp mimics**			
2	**DR-5663**<br>**ppApp**	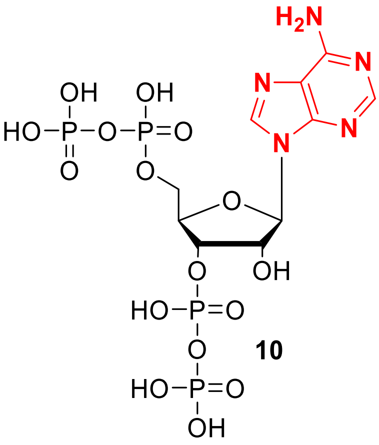 10	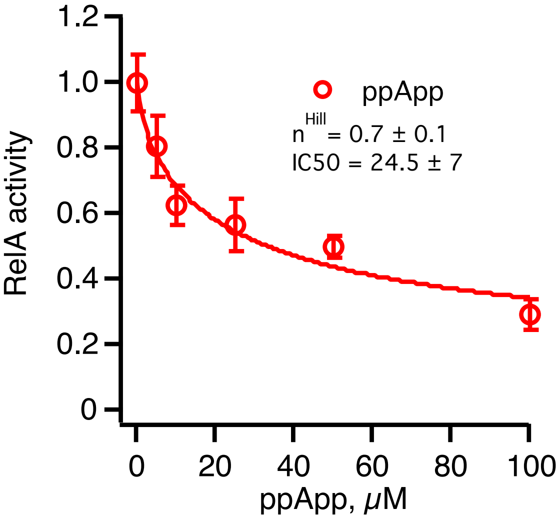

3	**Thio-ppGpp**	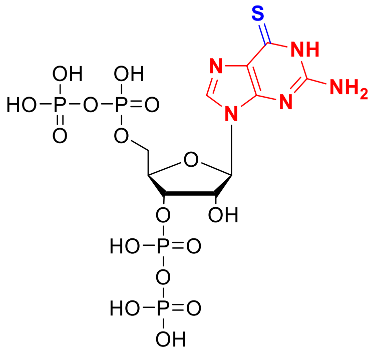	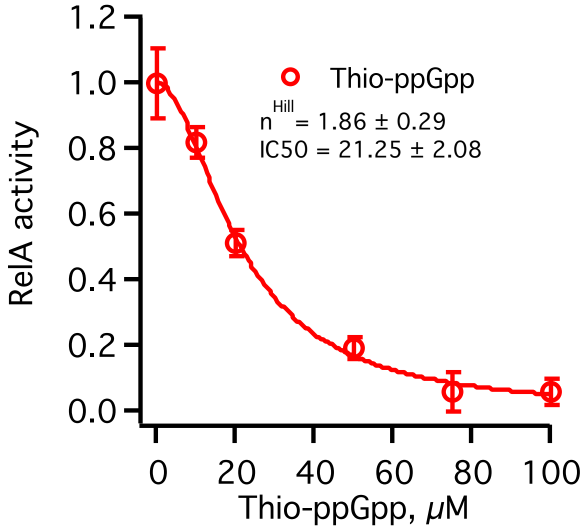
4	**DR-4250**	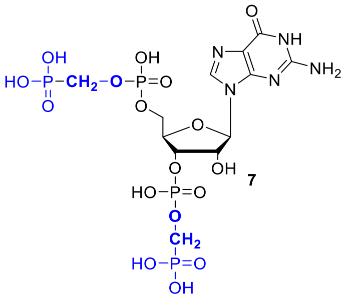 7	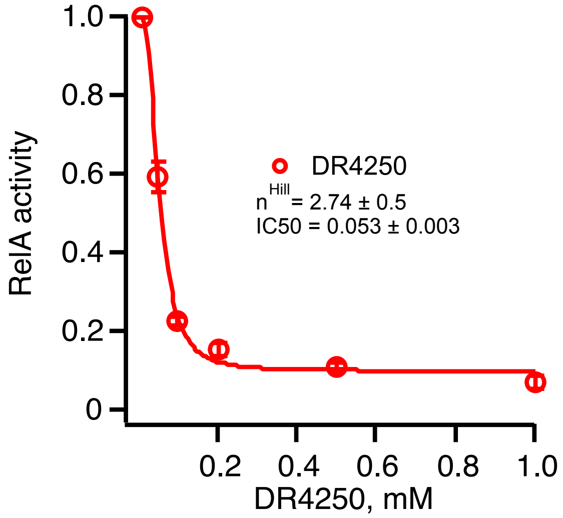
5	**DR-4239**	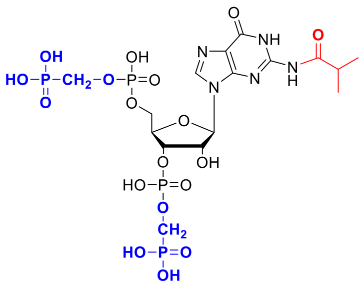	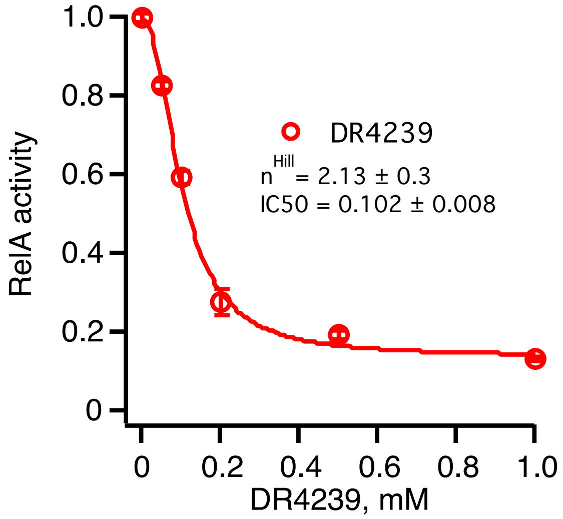

6	**DR-4238**	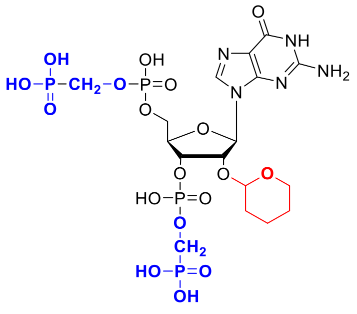	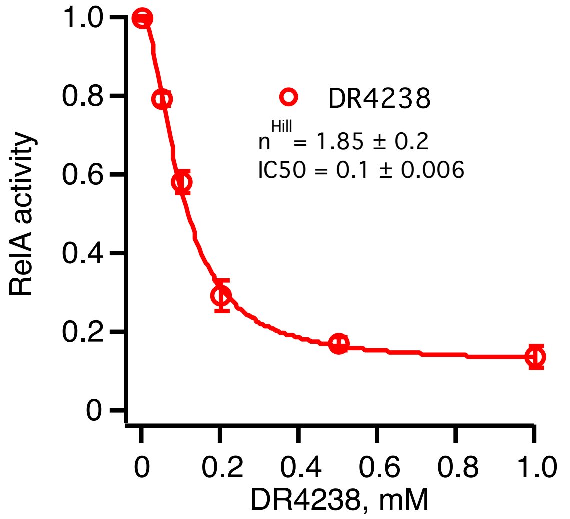
7	**DR-6241A**	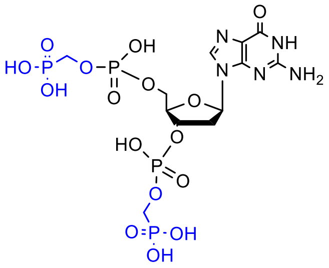	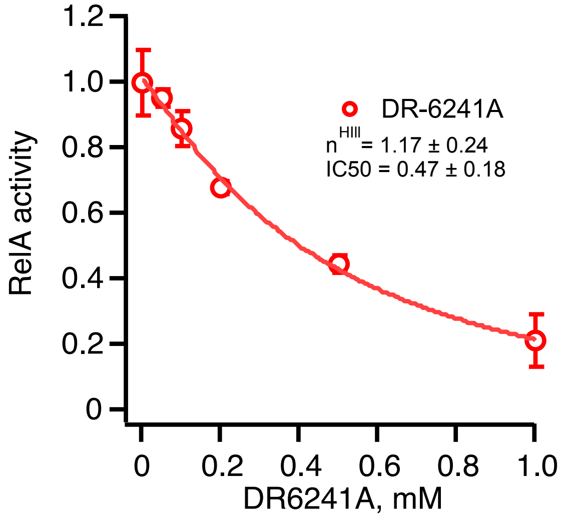
8	**DR-6222**	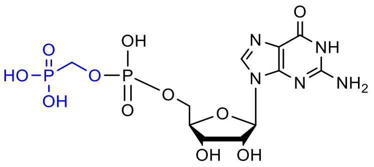	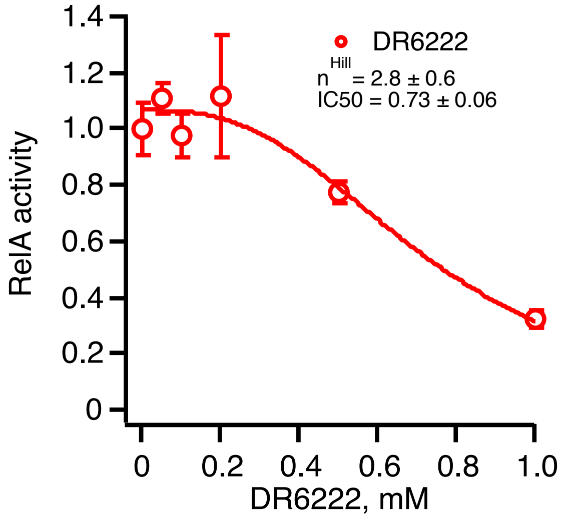

<table>
<tr><td>9</td><td>DR-5799C</td><td>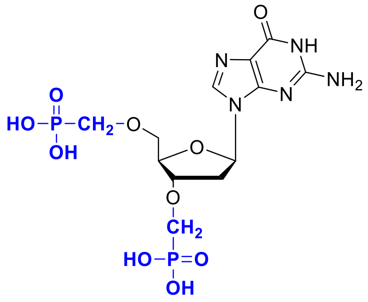</td><td>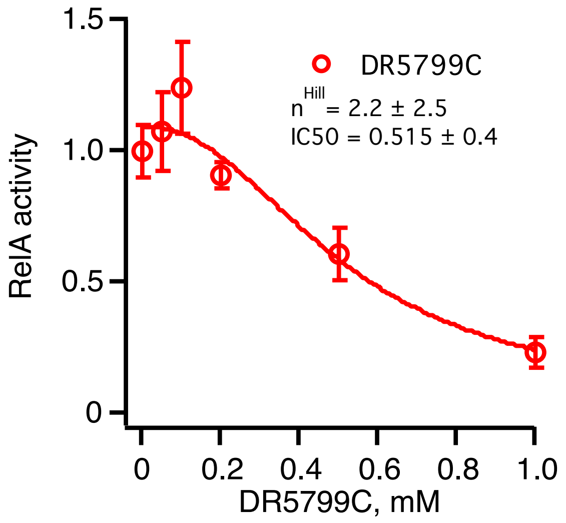
</td></tr>
<tr><td>10</td><td>DR-6331</td><td>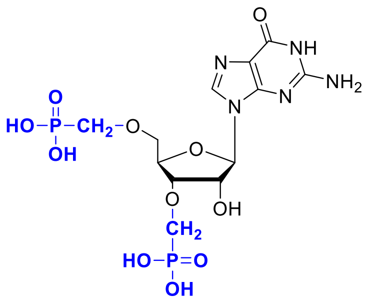</td><td>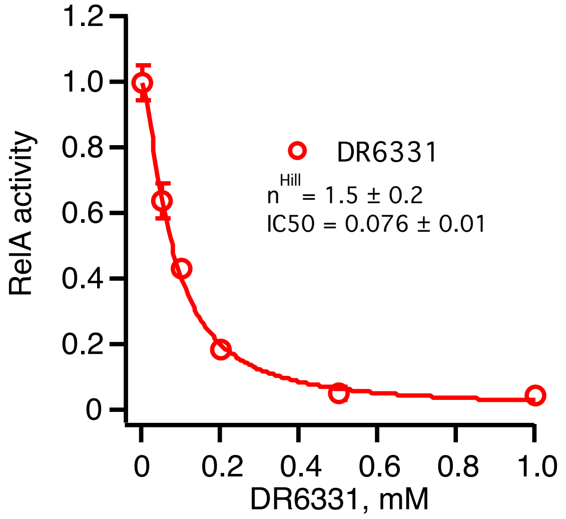
</td></tr>
<tr><td>11</td><td>DR-5824A</td><td>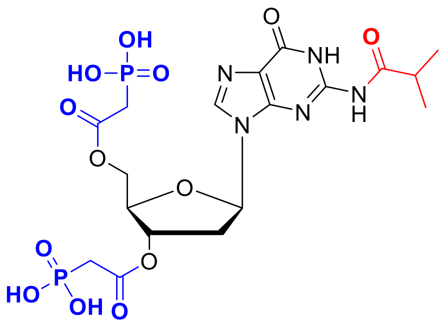</td><td>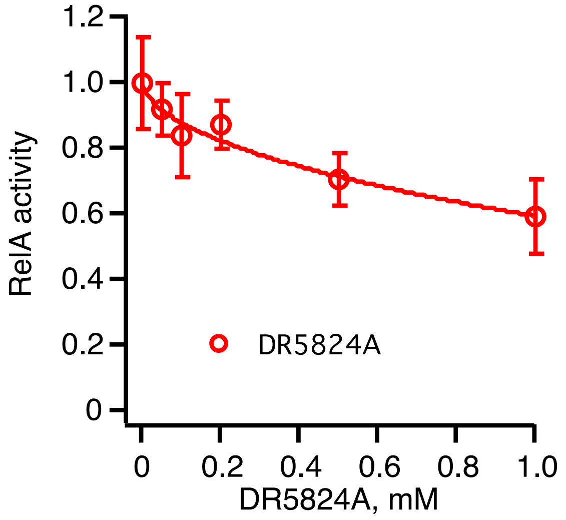
</td></tr>
</table>

12	**DR-5825A**	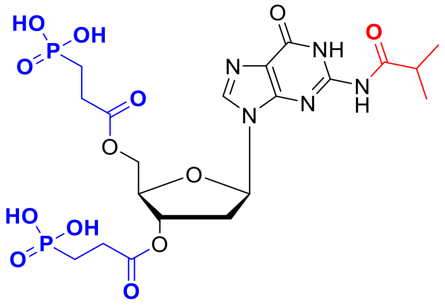	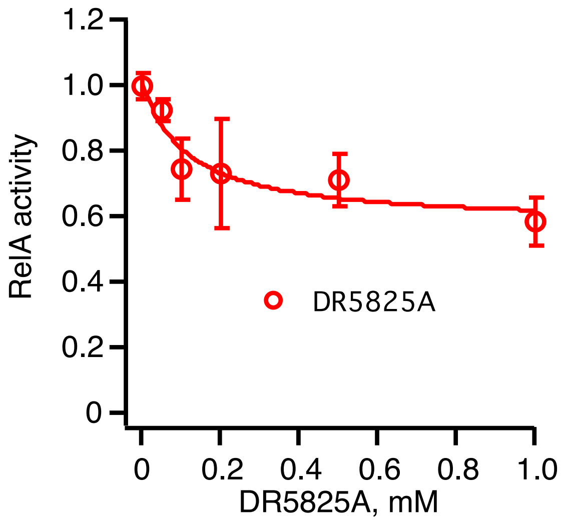
13	**DR-5839A**	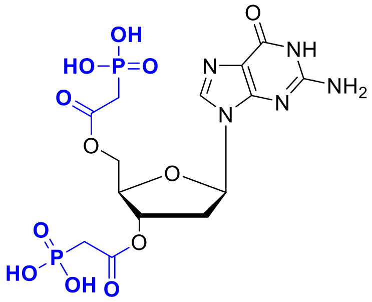	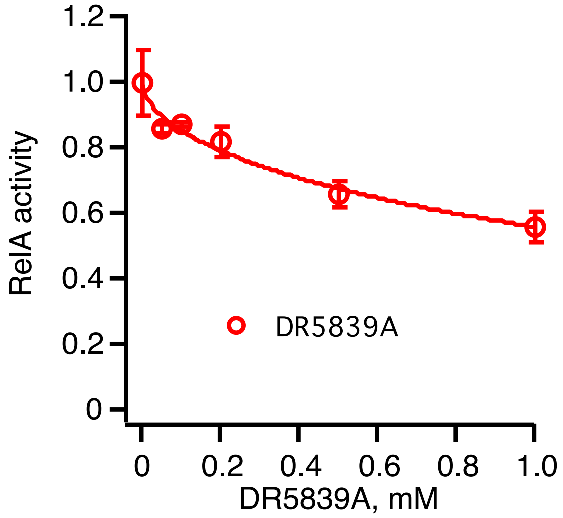
14	**DR-5835**	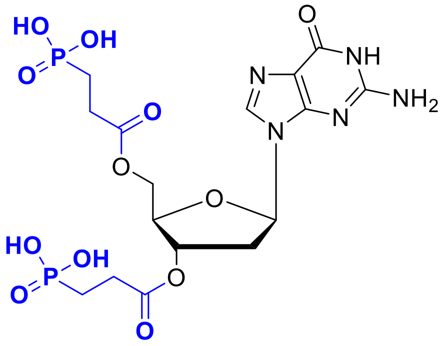	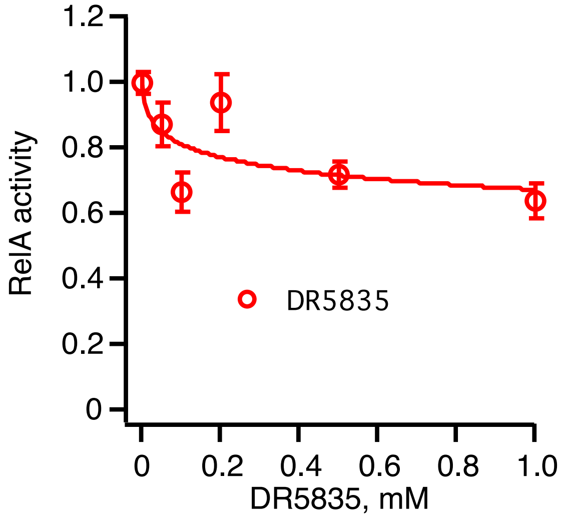

15	**DR-5836**	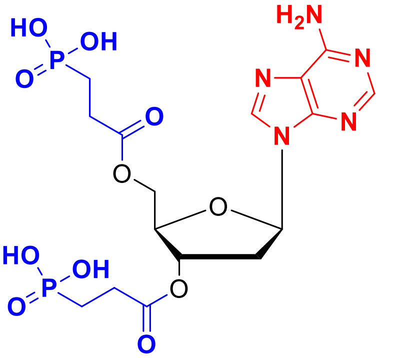	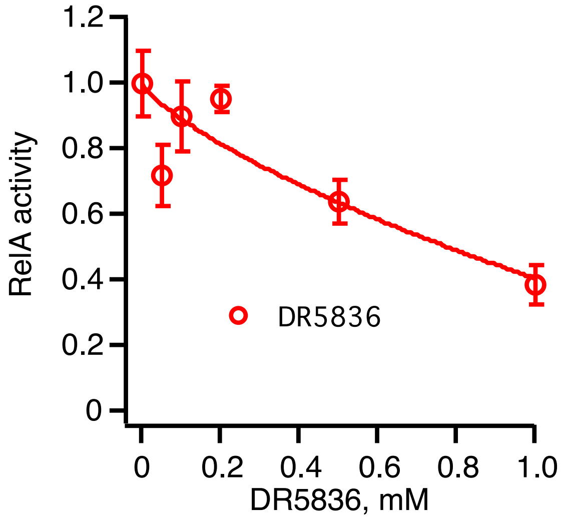
		**Relacin**	
16	**Relacin**	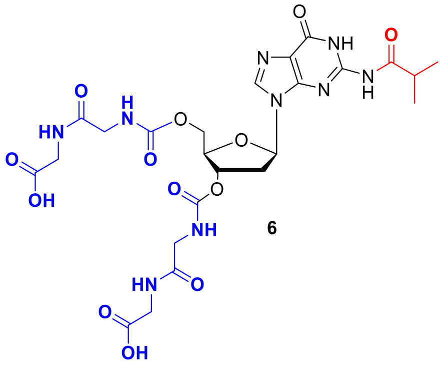	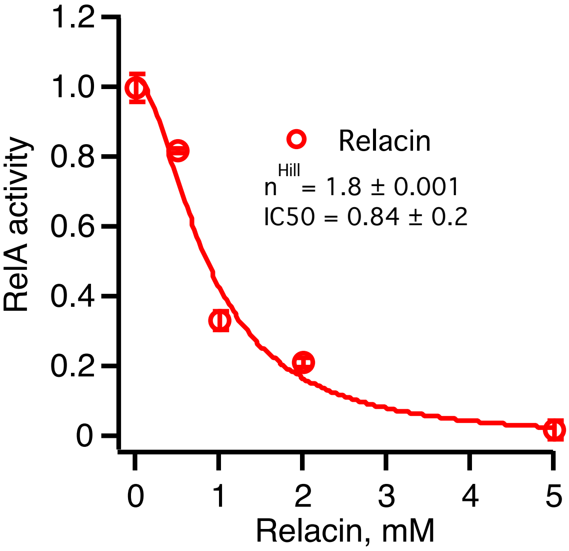
17	**DiBuRel**	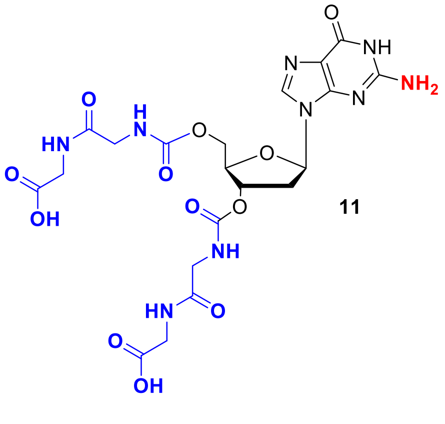	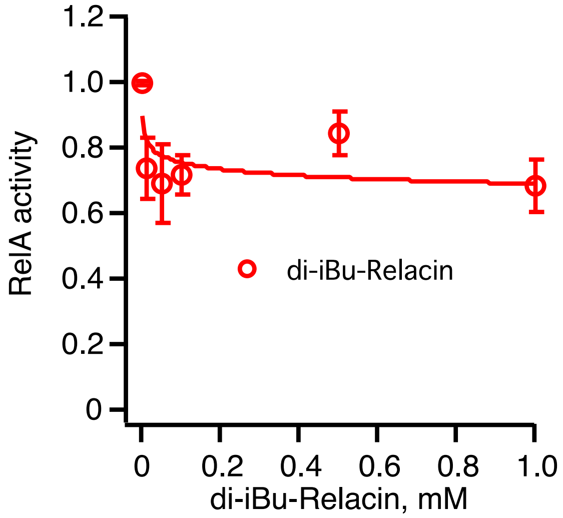

<table>
<tr>
<td>18</td>
<td>DR-5732</td>
<td>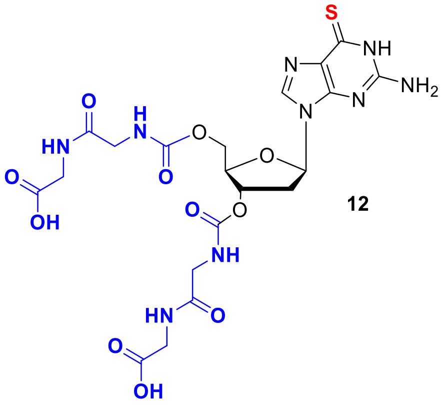

12</td>
<td>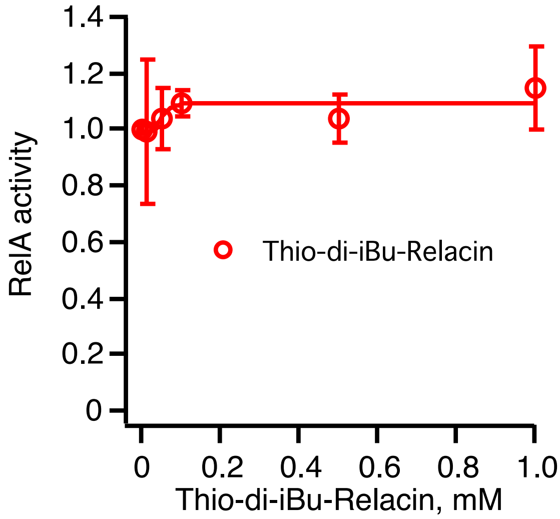
</td>
</tr>
<tr>
<td colspan="4">Piperidine phosphonates</td>
</tr>
<tr>
<td>19</td>
<td>DR-M011</td>
<td>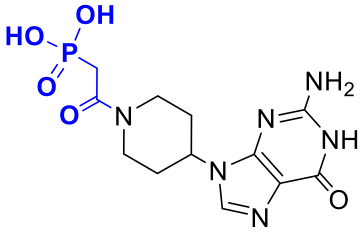

14</td>
<td>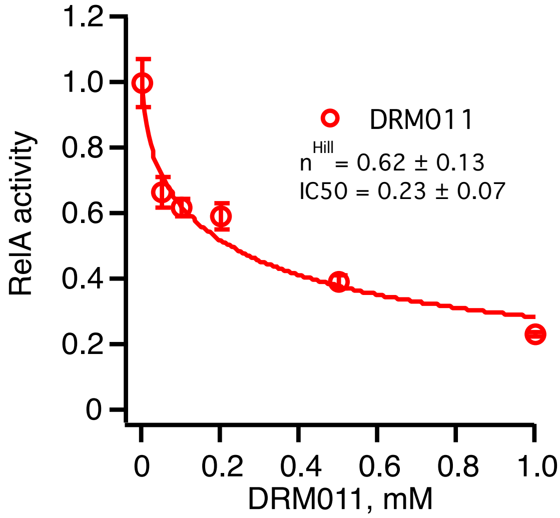
</td>
</tr>
<tr>
<td>20</td>
<td>DR-M014</td>
<td>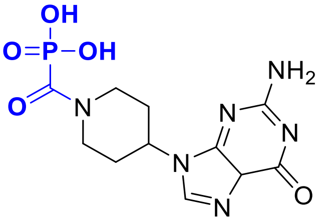

15</td>
<td>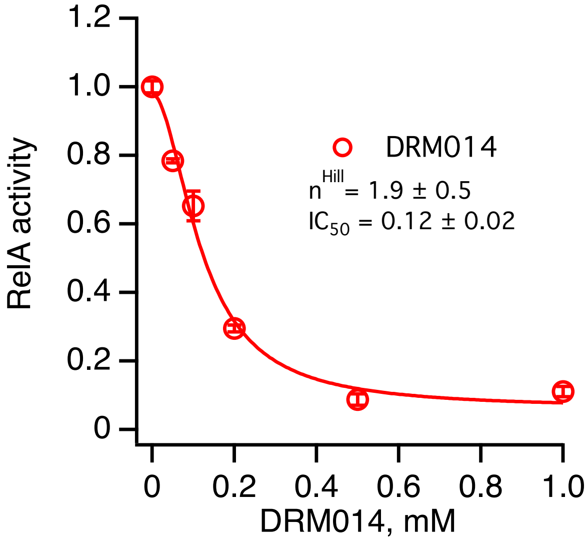
</td>
</tr>
</table>

21	**DR-6011B**	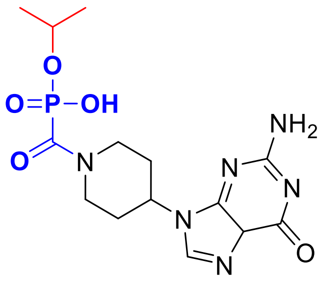 **16**	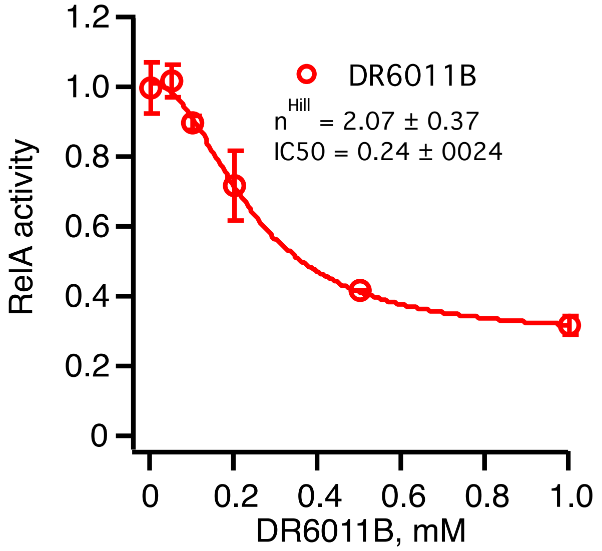
22	**DR-5994A**	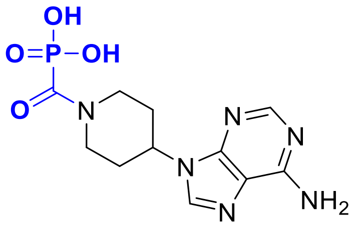	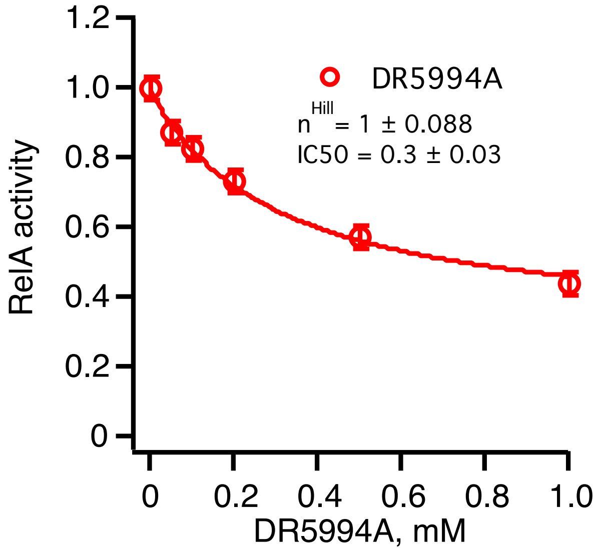
23	**DR-5994B**	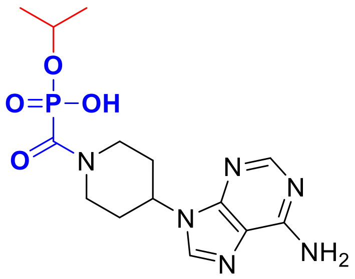	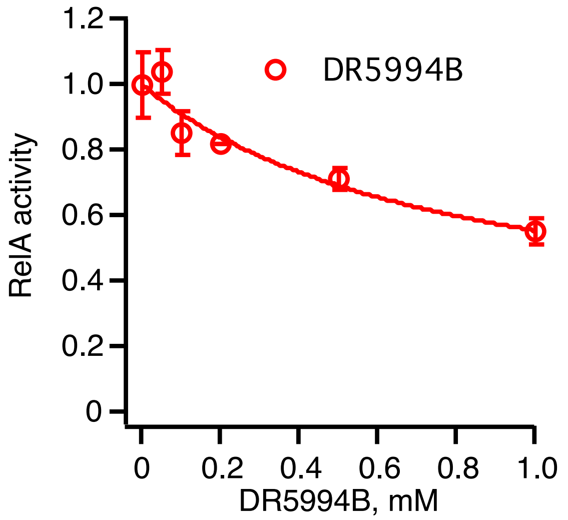

24	**DR-5165-B**	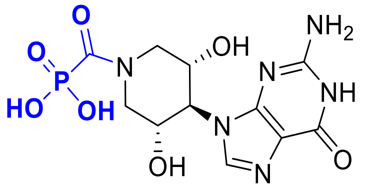	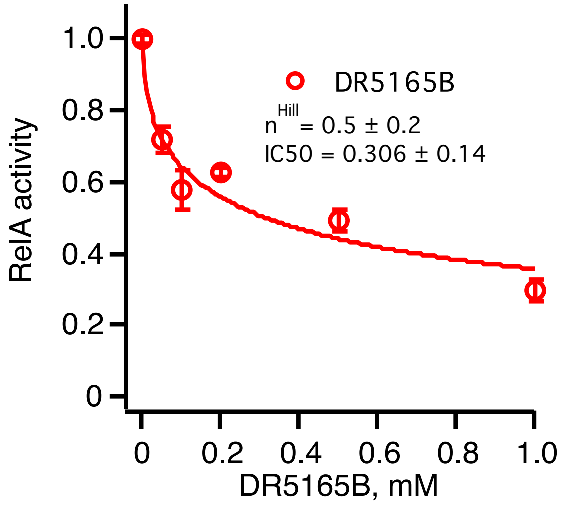
25	**DR-5320**	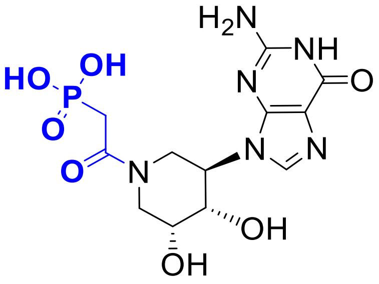	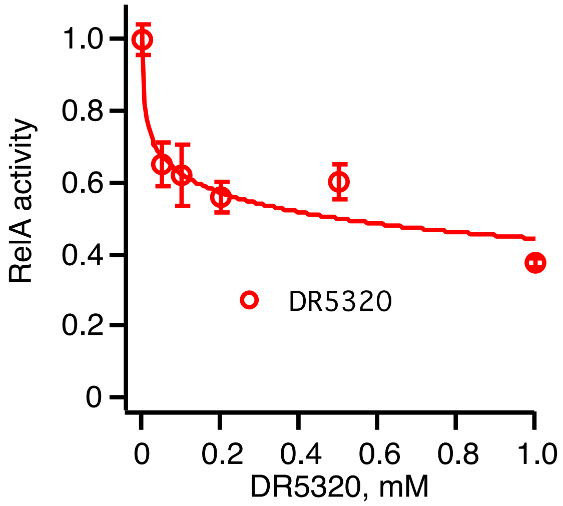
26	**DR-5319F4**	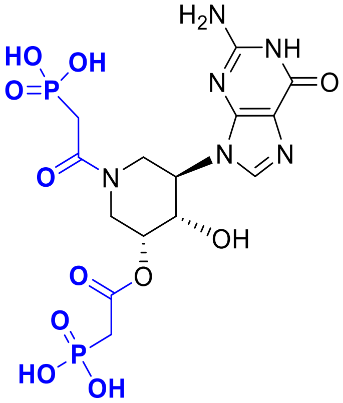	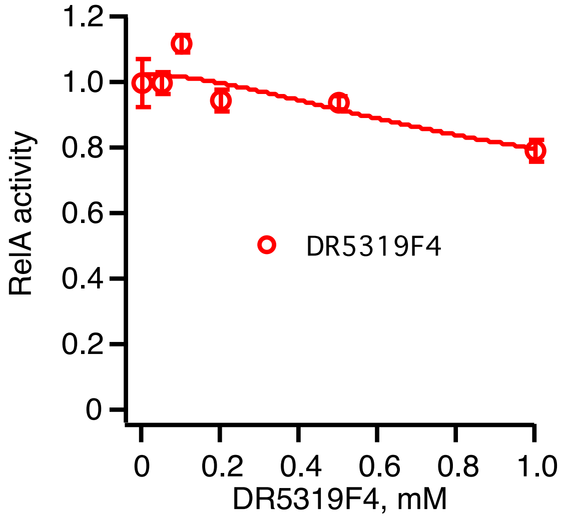

27	**K839**	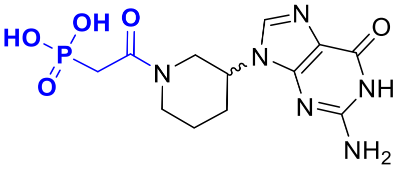	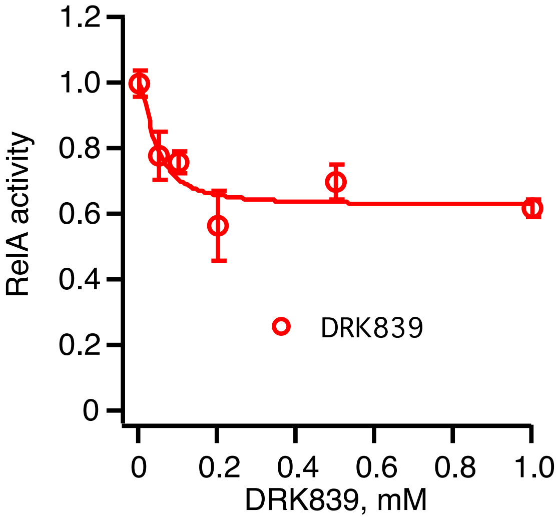
28	**K850**	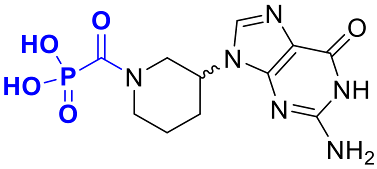	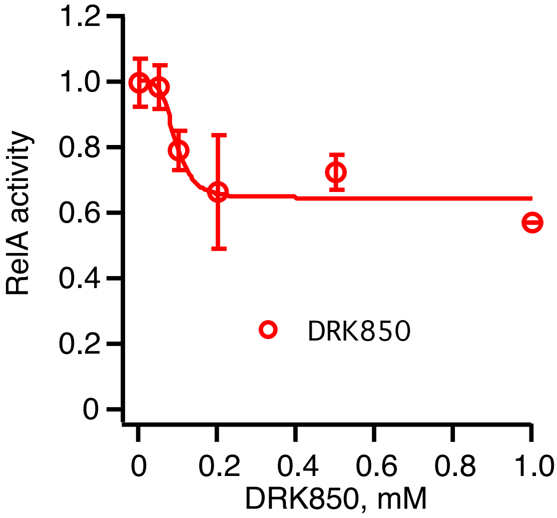
29	**K775**	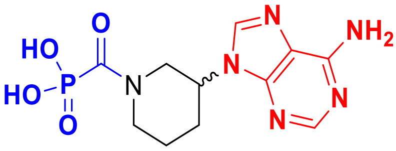	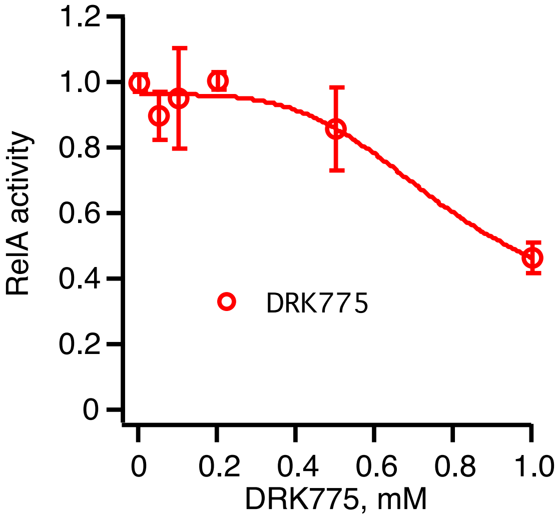

30	**K759**	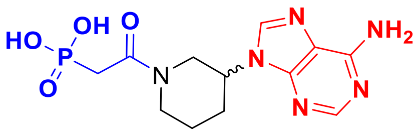	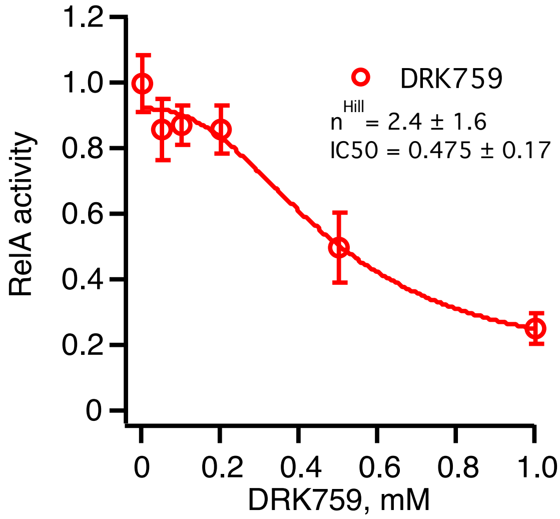
31	**K811**	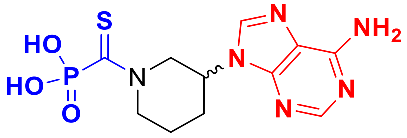	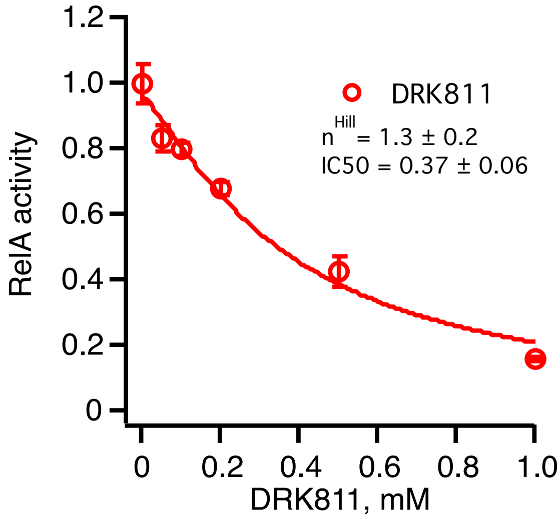
**Pyrrolidine phosphonates**			
32	**DR-4520**	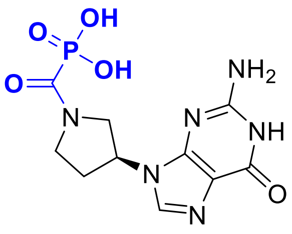 **17**	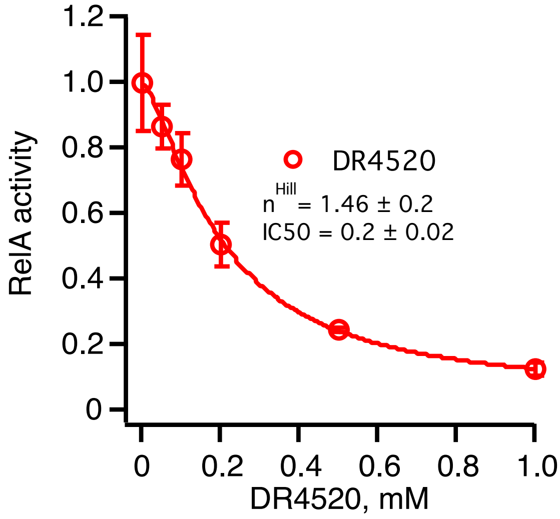

33	**DR-5267**	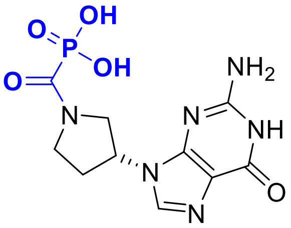 **18**	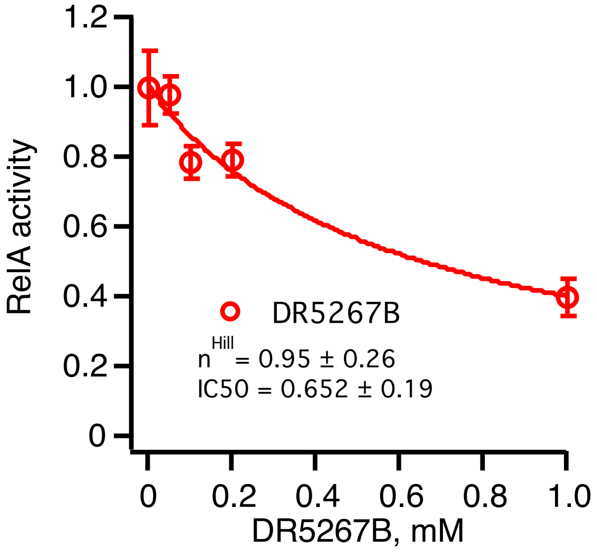
34	**DR-4518**	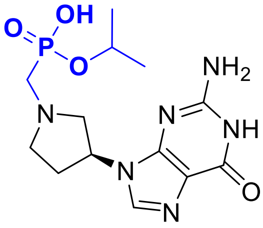 **19**	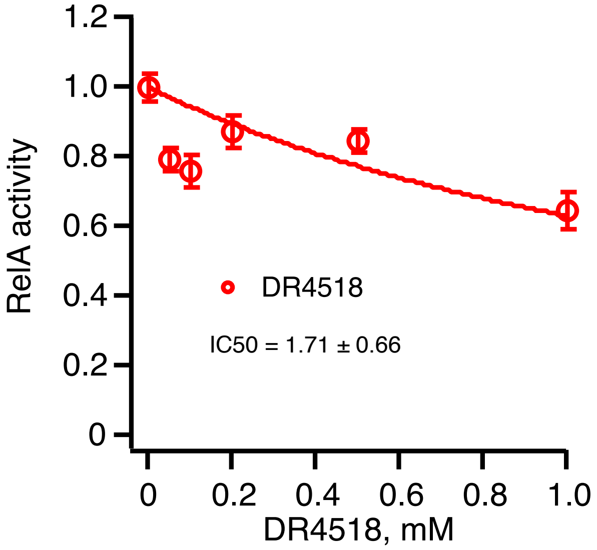
35	**DR-5256**	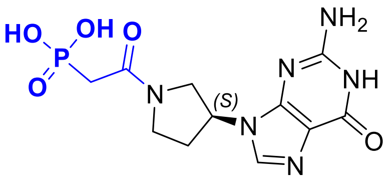	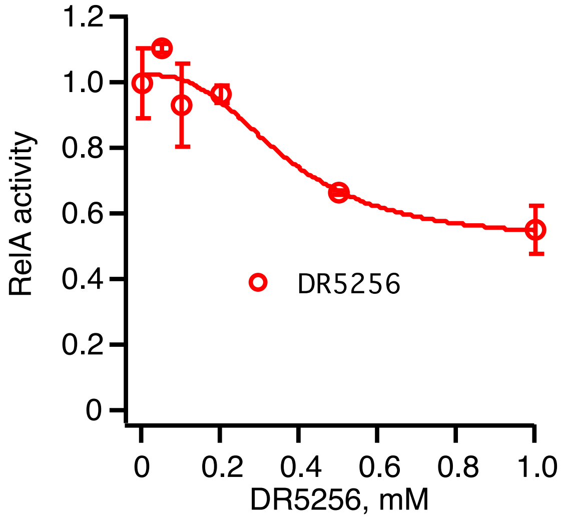

36	**DR-3781**	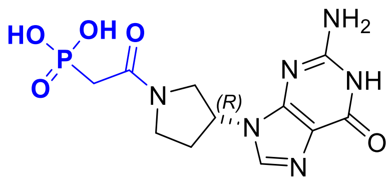	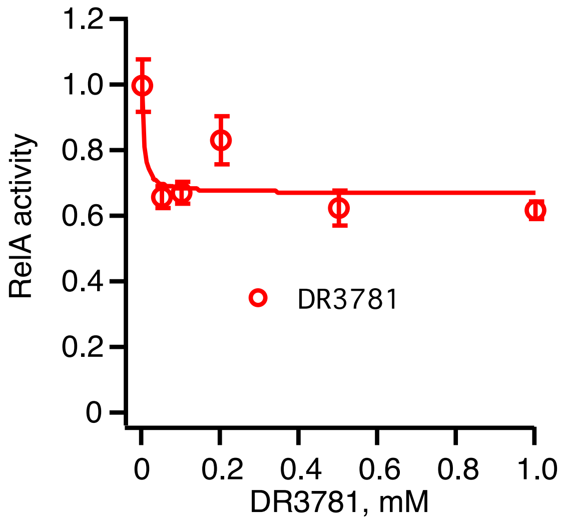
37	**DR-5580**	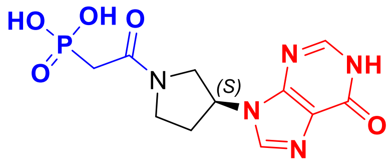	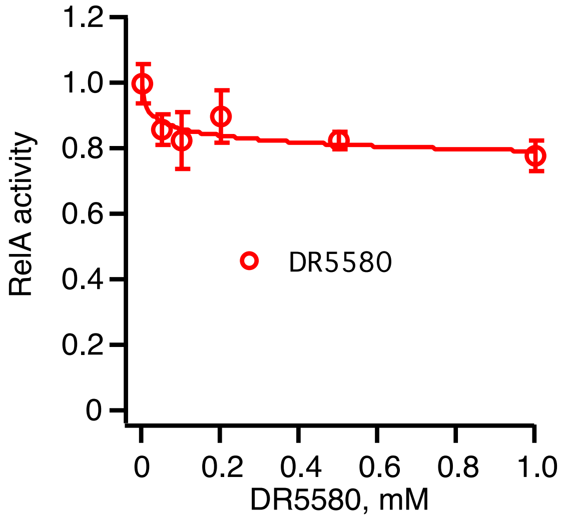
38	**DR-5418-A**	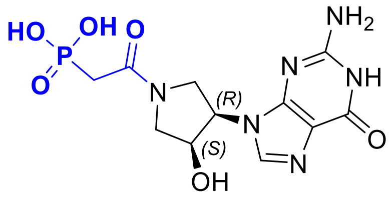	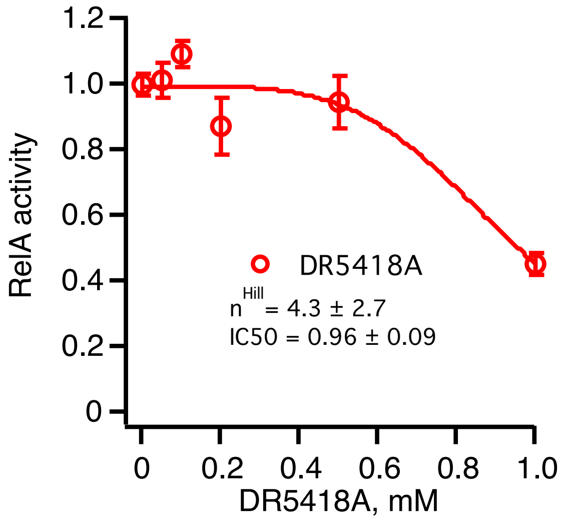

39	**DR-5269**	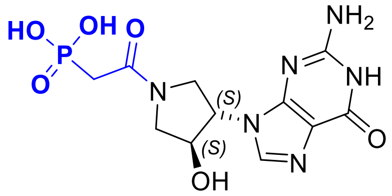	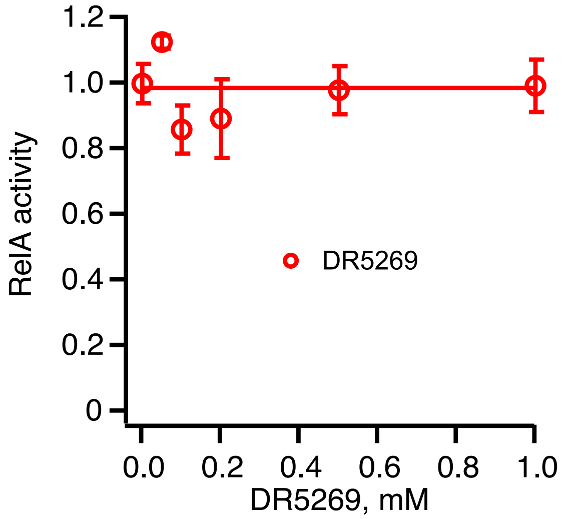
40	**DR-5336A**	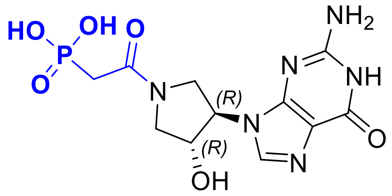	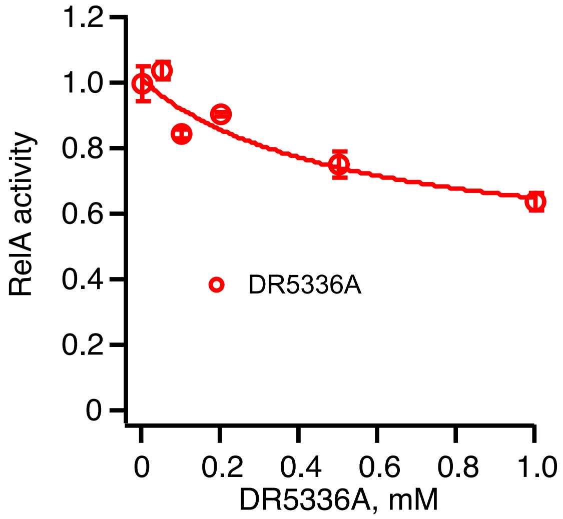
41	**DR-4661**	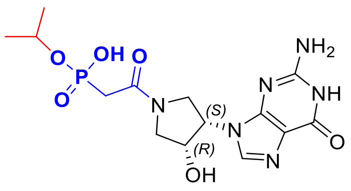	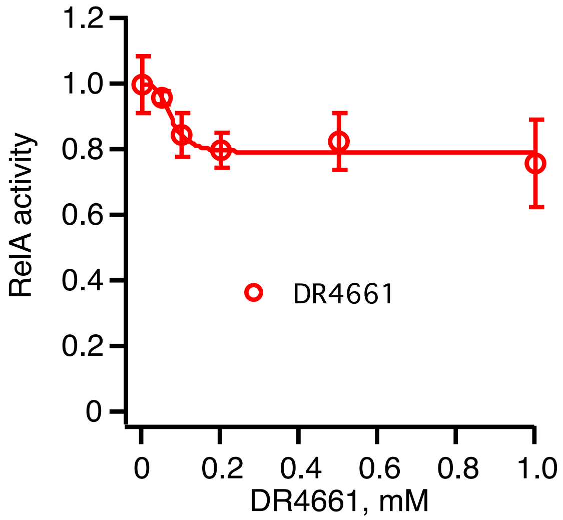

<table>
<tr><td>42</td><td>DR-5191A</td><td>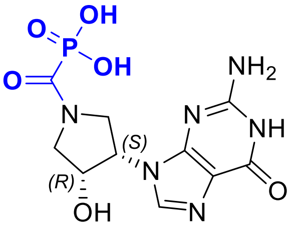
</td><td>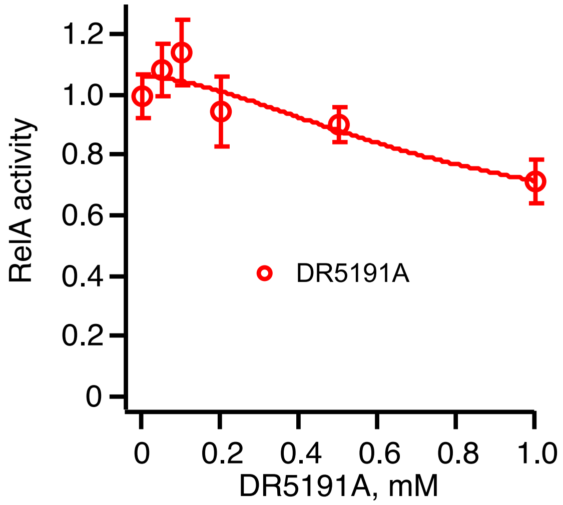
</td></tr>
<tr><td>43</td><td>DR-5300</td><td>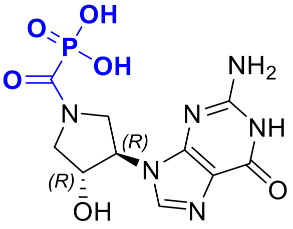
</td><td>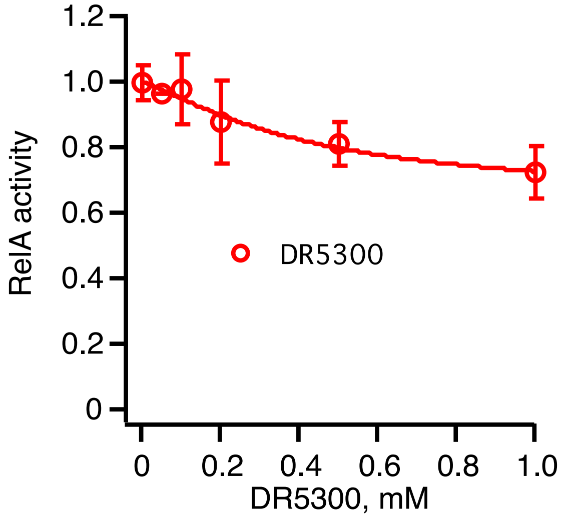
</td></tr>
<tr><td>44</td><td>DR-5191B</td><td>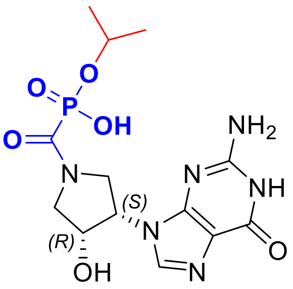
</td><td>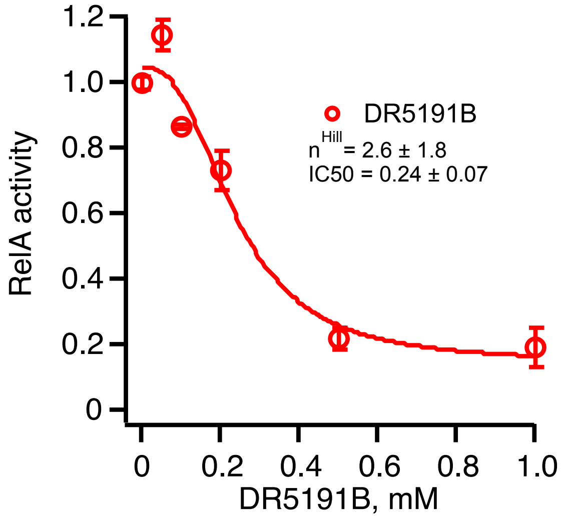
</td></tr>
</table>

45	**DR-4871**	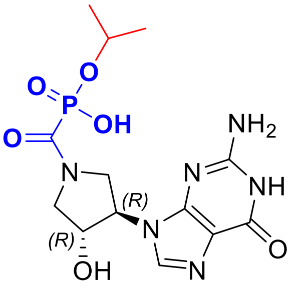	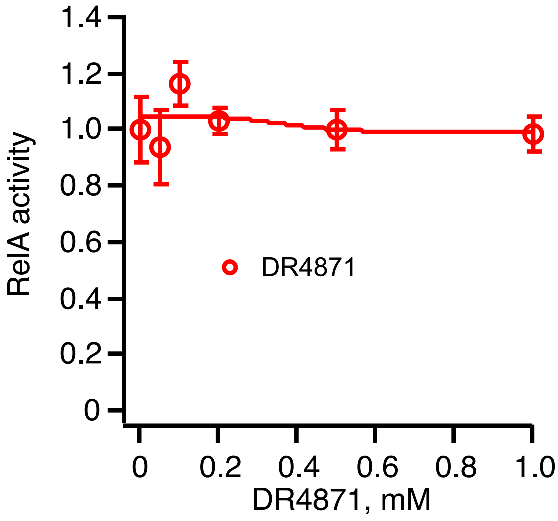
46	**DR-5788**	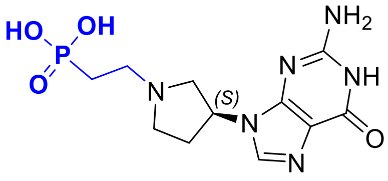	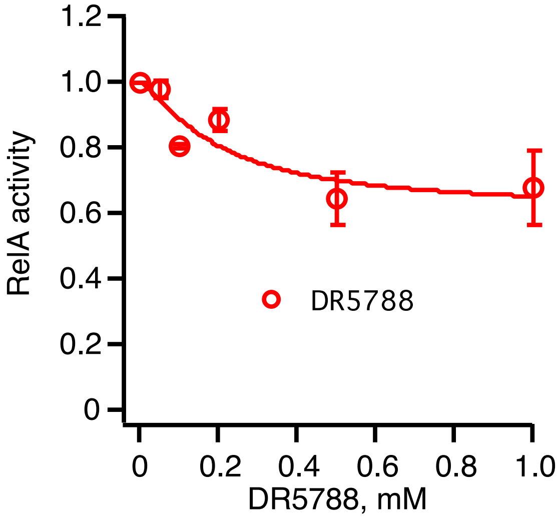
47	**DR-4516**	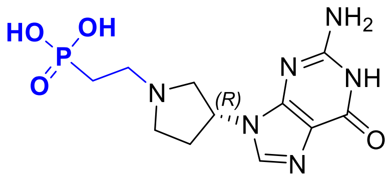	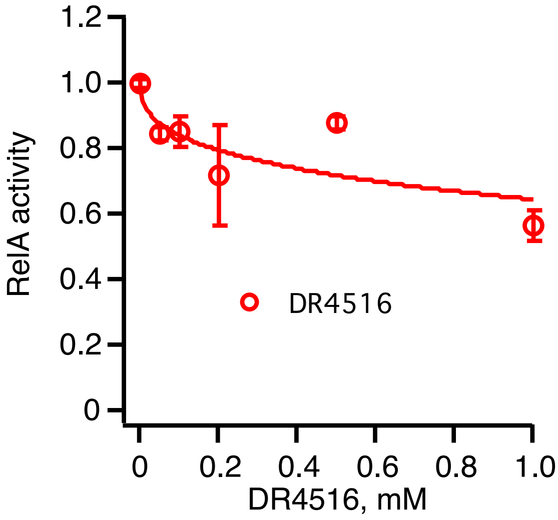

48	**DR-5787**	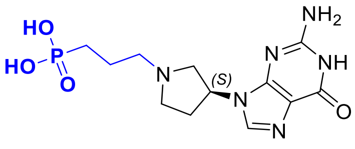	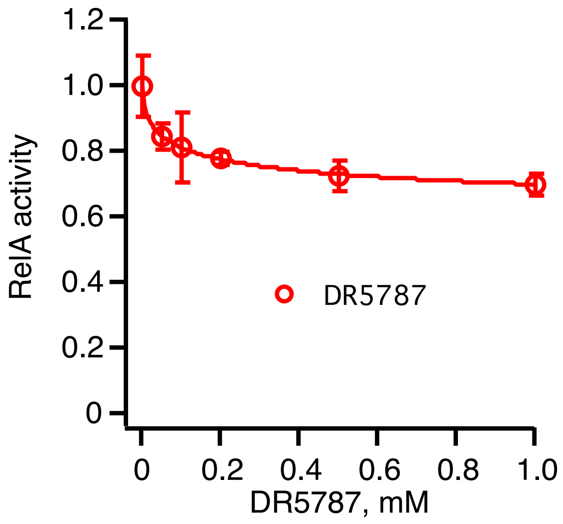
49	**DR-5786**	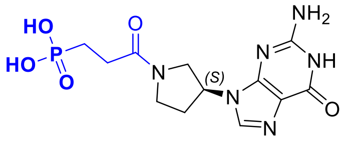	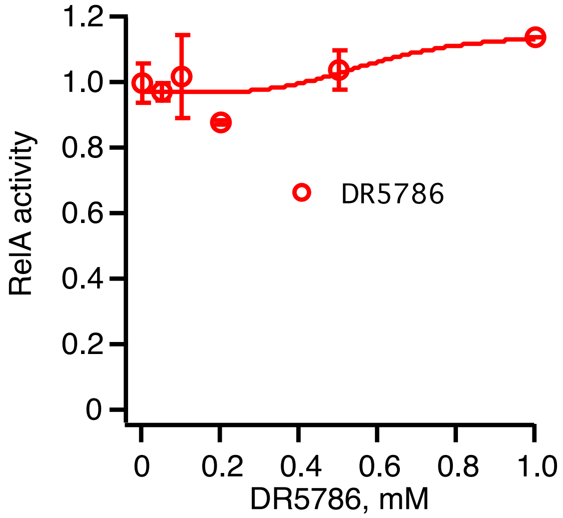
50	**DR-5279**	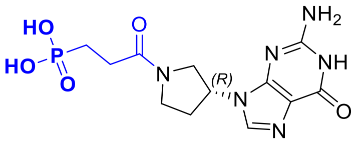	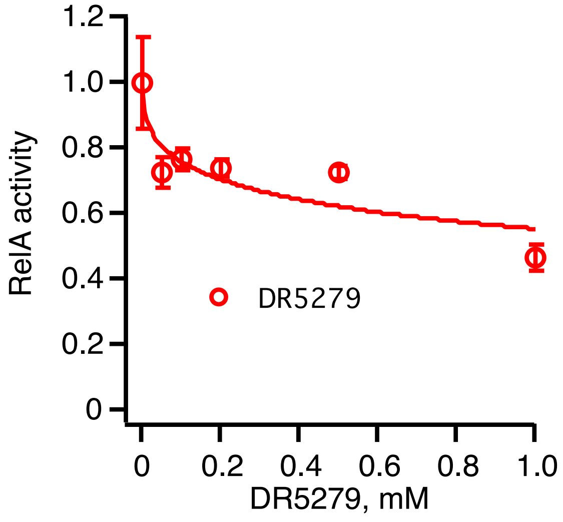

<table>
<tr><td>51</td><td>DR-4771</td><td>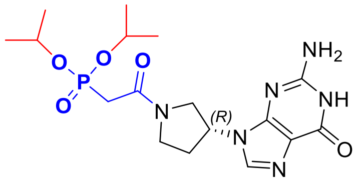
</td><td>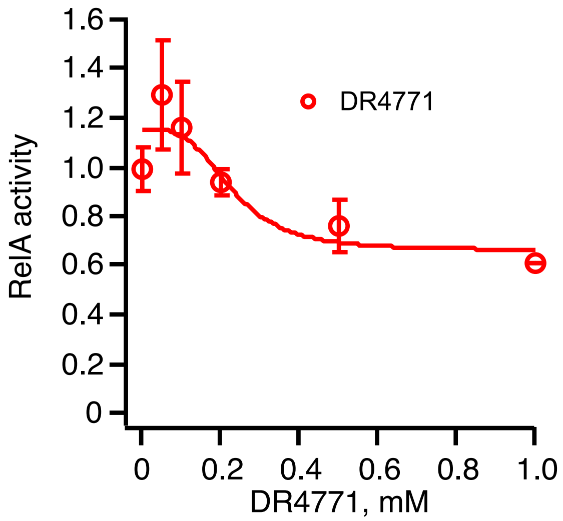
</td></tr>
<tr><td>52</td><td>DR-5891</td><td>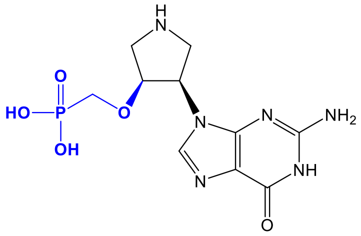
</td><td>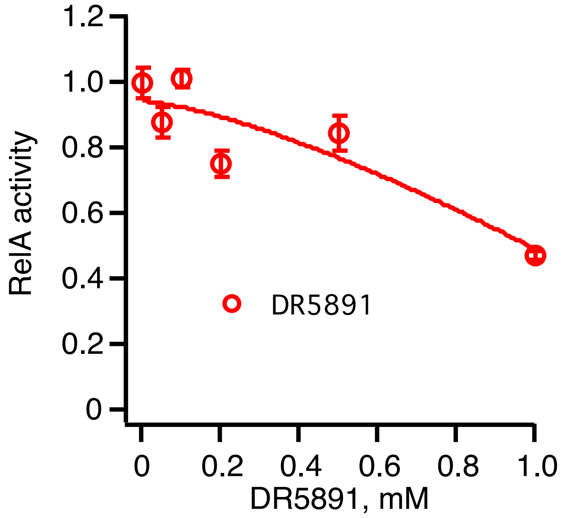
</td></tr>
<tr><td>53</td><td>DR-5895</td><td>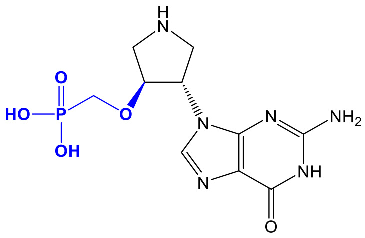
</td><td>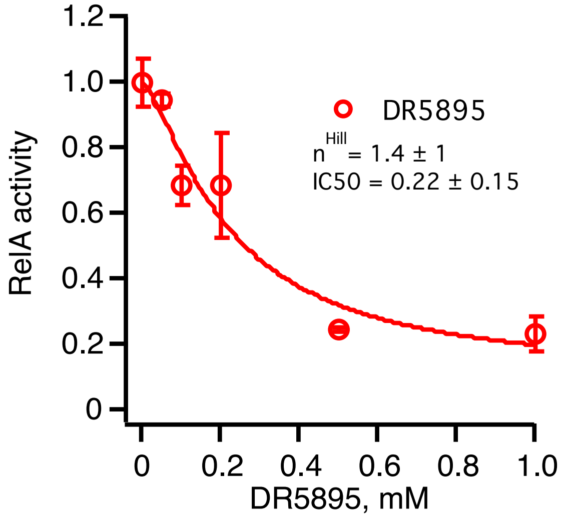
</td></tr>
<tr><td colspan="4">Prolinol phosphonates</td></tr>
</table>

54	**DR-5327**	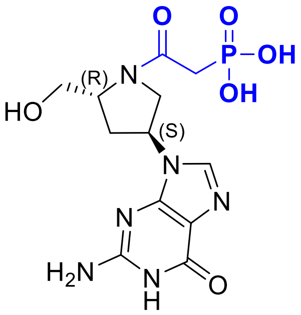	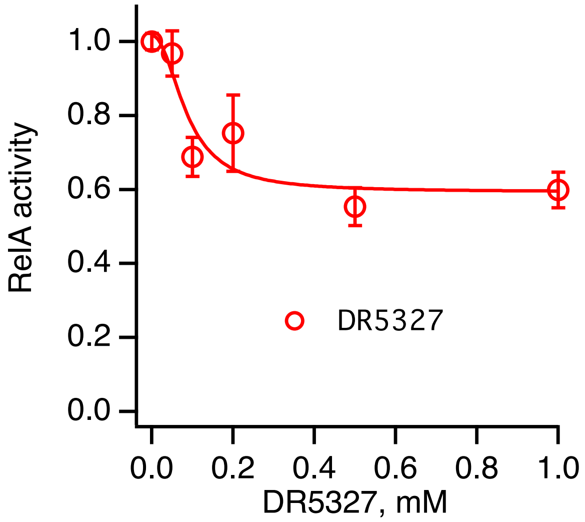
55	**DR-5247**	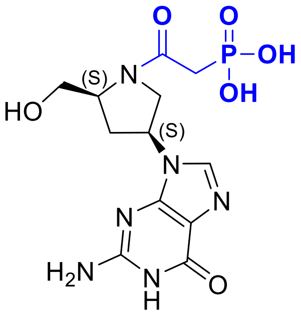	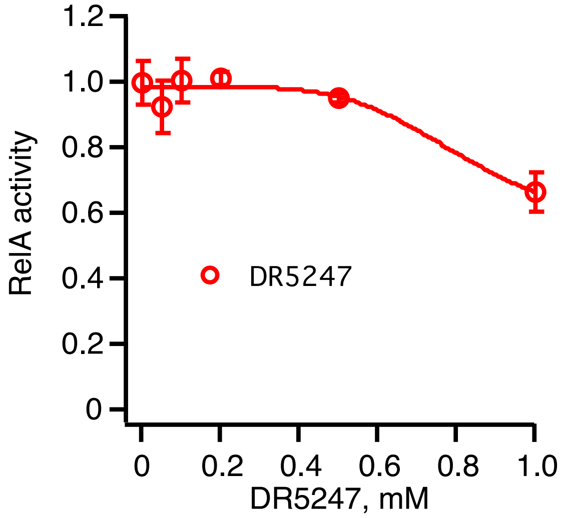
56	**DR-5234**	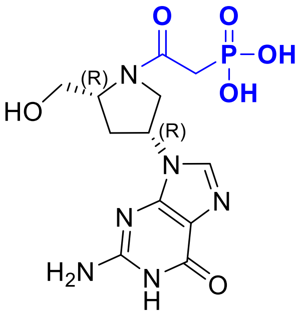	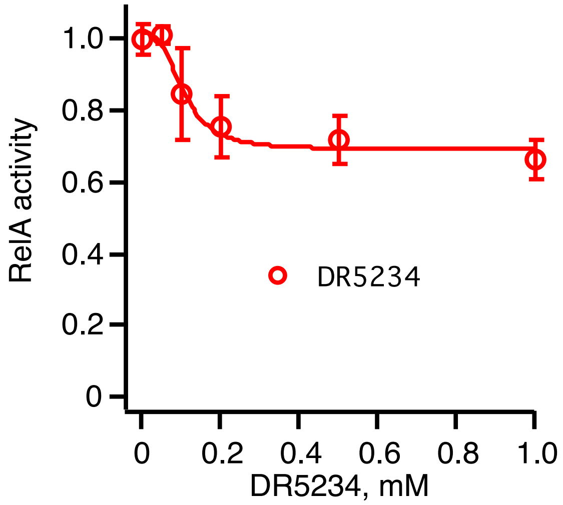

57	**DR-5294-A**	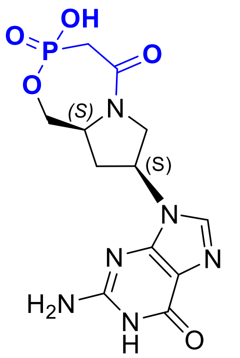	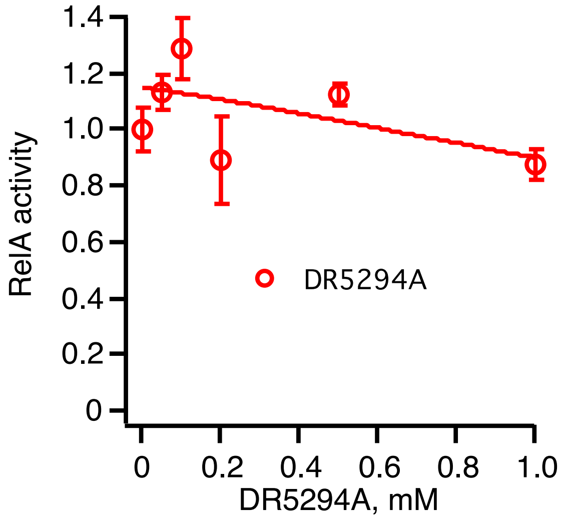
		**Acyclic phosphonates**	
58	**DR-5163**	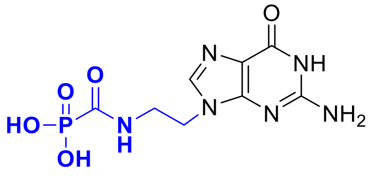 **20**	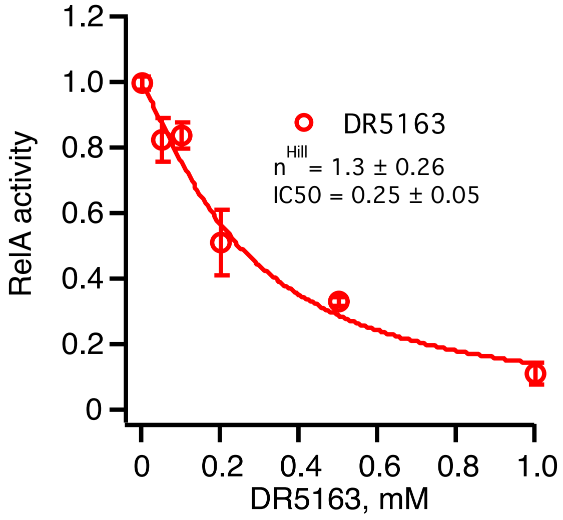
59	**DR-5164**	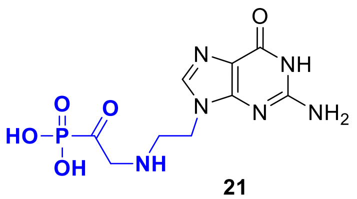 **21**	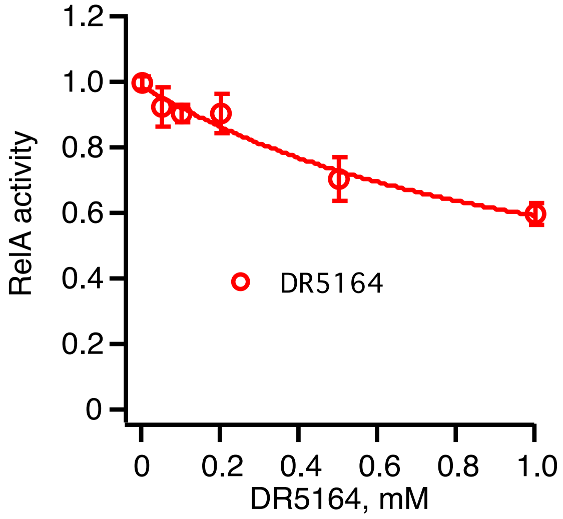

60	**DR-4590B**	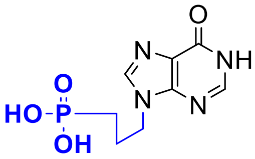	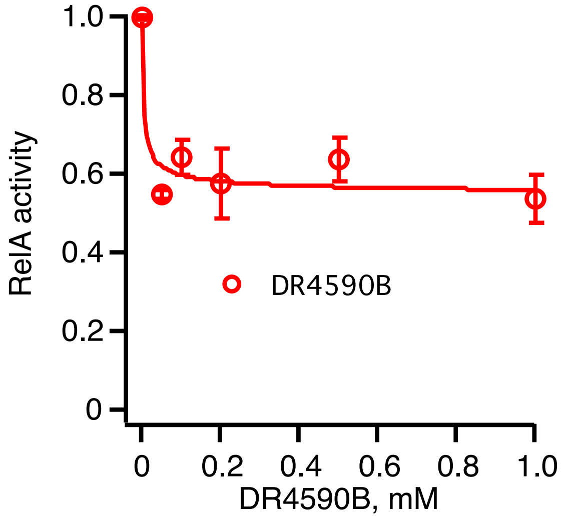
**Azetidine phosphonates**			
61	**DR-5415**	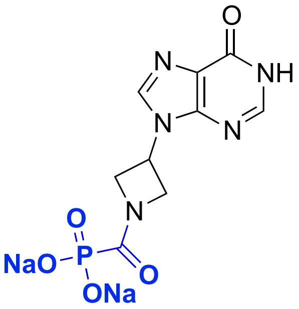	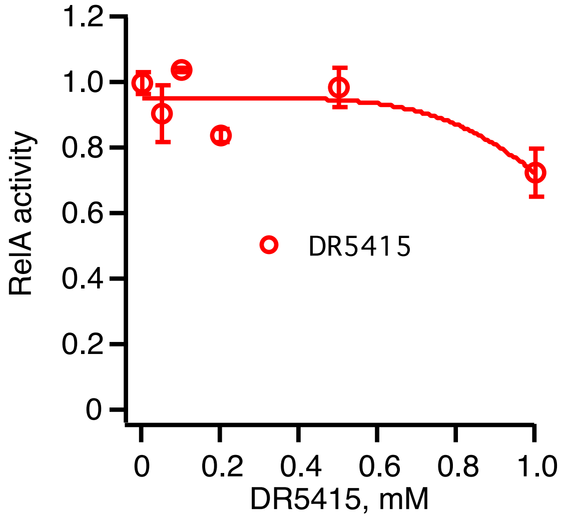
62	**DR-5407**	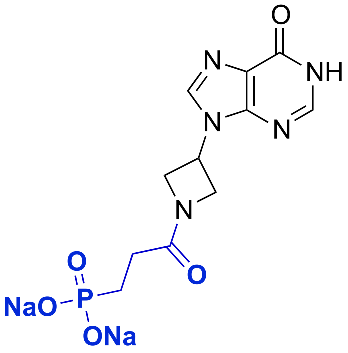	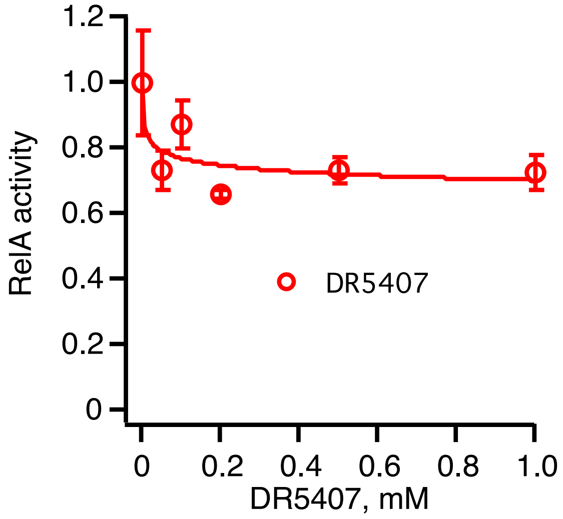

63	**DR-5410-A**	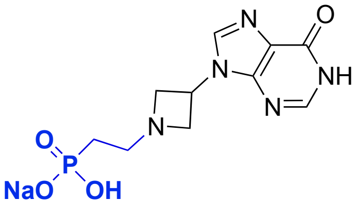	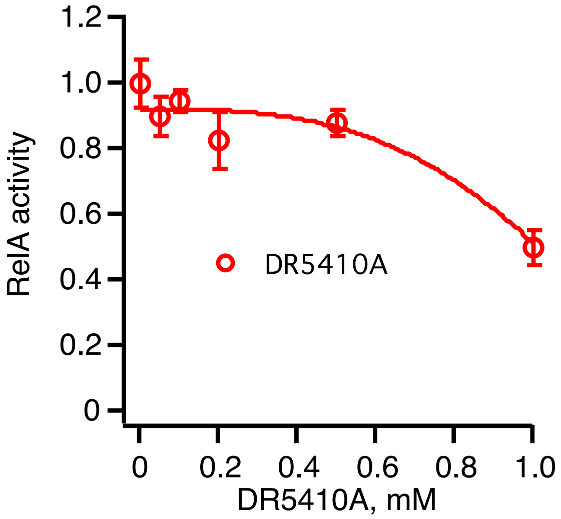
64	**DR-5406**	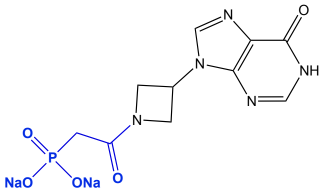	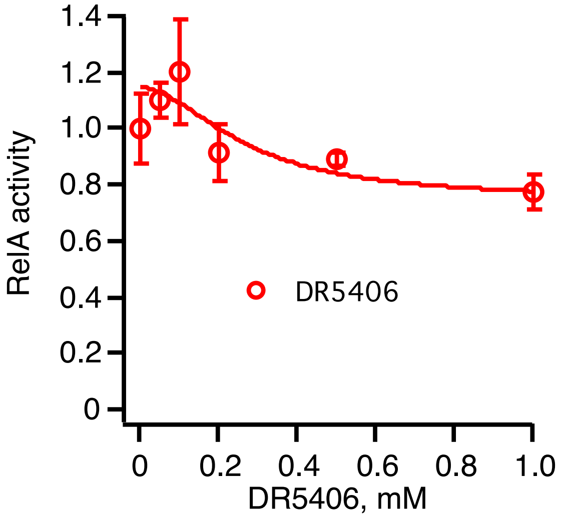
**Naturally occurring signaling nucleotides**			
65	**NADH**	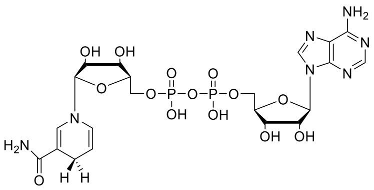	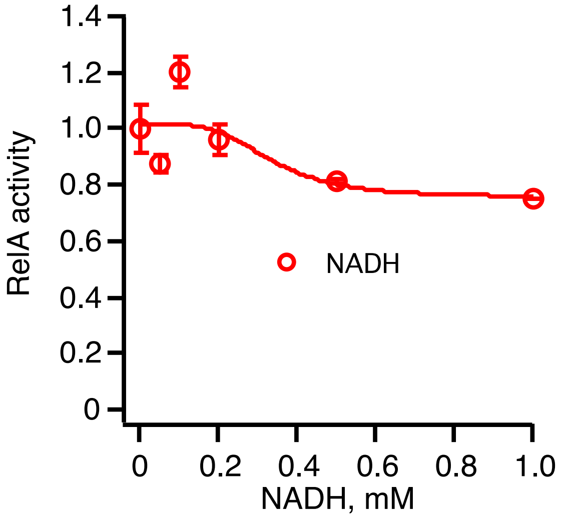

<table>
<tr><td>66</td><td>NADPH</td><td>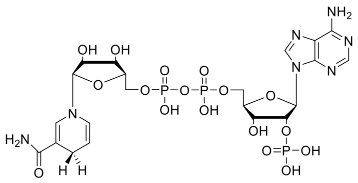</td><td>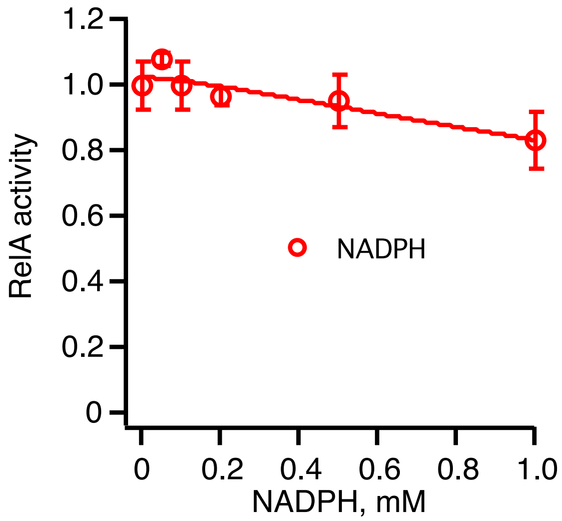
</td></tr>
<tr><td>67</td><td>c-di-AMP</td><td>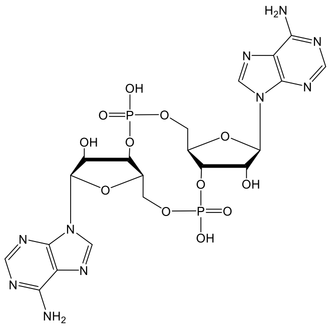</td><td>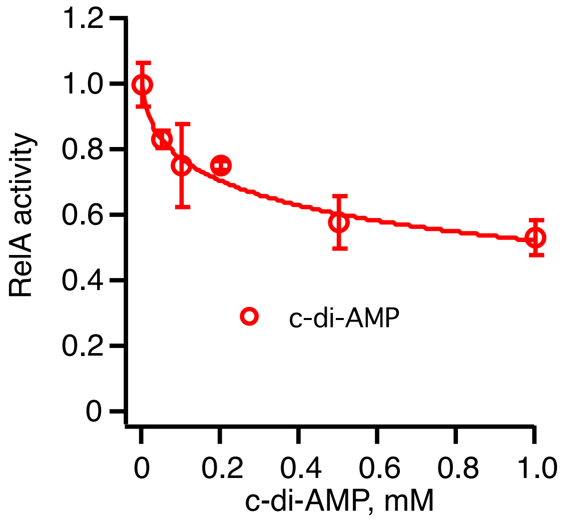
</td></tr>
<tr><td>68</td><td>c-di-GMP</td><td>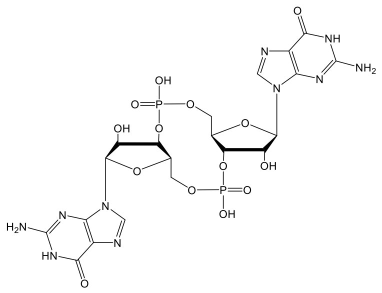</td><td>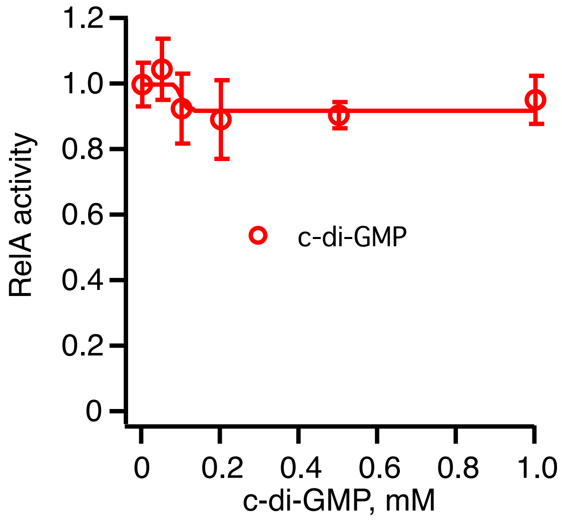
</td></tr>
</table>

69	**c-GAMP**	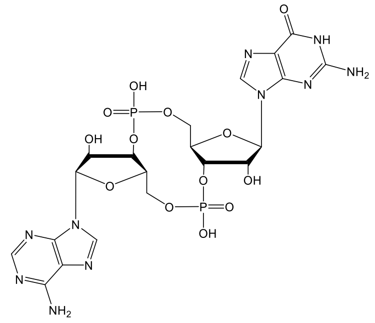	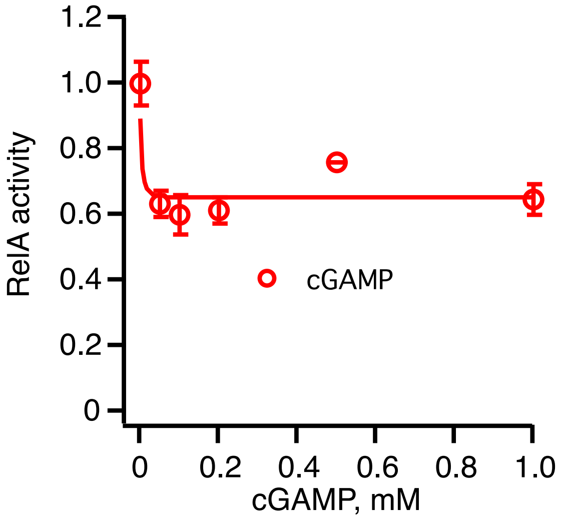
**Foscarnet**			
70	**Foscarnet**	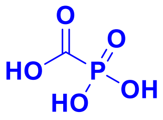	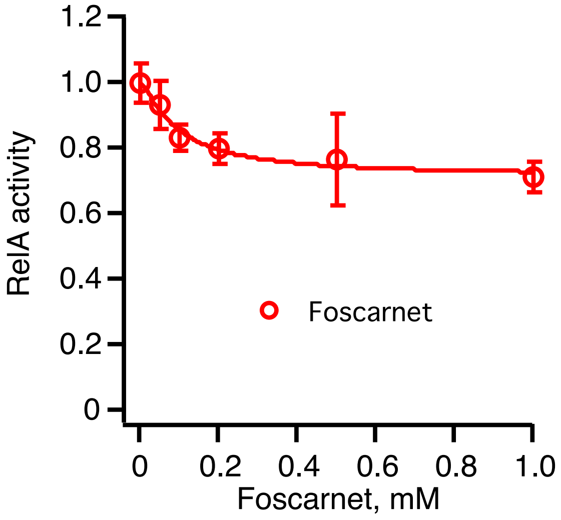

Synthesis and characterization of tested compounds

General

Unless stated otherwise, all used solvents were anhydrous. TLC was performed on silica gel pre-coated aluminium plates Silica gel/TLC-cards, UV 254 (Fluka), and compounds were detected by UV light (254 nm), by heating (detection of dimethoxytrityl group; orange color), by spraying with 1% solution of ninhydrine to visualize amines, and by spraying with 1% solution of 4-(4-nitrobenzyl)pyridine in ethanol followed by heating and treating with gaseous ammonia (blue color of mono- and diesters of phosphonic acid). Preparative column chromatography was carried out on silica gel (40-60μm; Fluka), and elution was performed at the flow rate of 40 ml/min. The following solvent systems were used for TLC and preparative chromatography: toluene-ethyl acetate 1:1 (T); chloroform-ethanol 9:1 (C1); ethyl acetate-acetone-ethanol-water 6:1:1:0.5 (H3); ethyl acetate-acetone-ethanol-water 4:1:1:1 (H1). The concentrations of solvent systems are stated in volume percents (%, v/v). Purity of prepared compounds was determined by LC-MS performed on Waters AutoPurification System with 2545 Quarternary Gradient Module and 3100 Single Quadrupole Mass Detector using LUNA C18, column (Phenomenex, 100 x 4.6 mm, 3 μm) at flow rate 1 ml/min. Typical conditions: mobile phase, A - 50mM NH_4HCO_3; B - 50 mM NH_4HCO_3 in 50% aq. CH_3CN; C – CH_3CN; A→B/10 min, B→C/10 min, C/5 min. Preparative RP HPLC was performed on LC5000 Liquid Chromatograph (INGOS-PIKRON, CR) using Luna C18 (2) column (250 x 21.2 mm, 5 μm) at flow rate of 10 ml/min by a gradient elution of methanol in 0.1M TEAB pH 7.5 (A = 0.1M TEAB; B = 0.1M TEAB in 50% aq. methanol; C = methanol) or without buffer. All final compounds were lyophilized from water. Mass spectra were recorded on LTQ Orbitrap XL (Thermo Fisher Scientific) using ESI ionization. NMR spectra were measured on Bruker AVANCE 400 (^{1}H at 400 MHz, ^{13}C at 100.6 MHz), Bruker AVANCE 500 and Varian UNITY 500 (^{1}H at 500 MHz, ^{13}C at 125.8 MHz) spectrometers. D_2O (reference (dioxane) = ^{1}H 3.75 ppm, ^{13}C 69.3 ppm. Chemical shifts (in ppm, δ scale) were referenced to TMS as internal standard; coupling constants (*J*) are given in Hz. All intermediates were determined by LC-MS.

ppGpp and **ppApp** (entry 1 and 2) were synthesized according to Schattenkerk et al. (1985)[9].

Thio-ppGpp (entry 3) was synthesized enzymatically following the same procedure as used for ppGpp using 6-thio-GDP (Jena) as a substrate.

Guanosine 3',5'-di(phosphonomethoxyphosphate) DR-4250 (entry 4) , 2-*N*-isobutyryl-guanosine 3',5'-di(phosphonomethoxyphosphate) DR-4239 (DR-6217) (entry 5), and 2-*N*-isobutyryl-2'-tetrahydropyranylguanosine 3',5'-di(phosphonomethoxyphosphate) DR-4238 (entry 6) were synthesized according to following scheme.

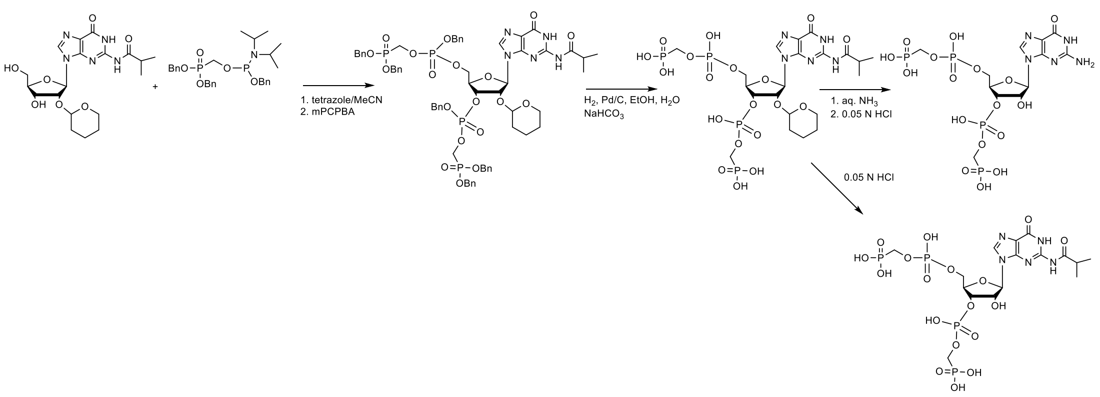

Scheme 1

Tetrazole (0.52 g, 7.4 mmol) was added to the solution of 2-*N*-isobutyryl-2'-tetrahydropyranylguanosine (0.23 g, 0.52 mmol) and benzyl ((bis(benzyloxy)phosphoryl)methyl) diisopropylphosphoramidite (0.7 g, 1.54 mmol) in MeCN (10 ml) under argon atmosphere. The reaction mixture was stirred for 40 min (followed by TLC in 10% EtOH in $CHCl_3$) and mCPBA (0.86 g, 5 mmol) was added. The reaction mixture was stirred additional 20 min and evaporated under reduced pressure. The fully protected intermediate was obtained by column chromatography on silica gel using linear gradient of ethanol in $CHCl_3$ as a mixture of diastereomers (characterized only by means of LCMS) in 94% yield (0.57 g, 0.49 mmol). The intermediate was dissolved in the mixture of EtOH (20 ml) and water (20 ml), $NaHCO_3$ (0.3 g, 3.5 mmol) and Pd/C (0.2 g) were added. The mixture was then hydrogenated overnight at the pressure of 100 kPa. After filtration over cellite the mixture was concentrated in vacuo and purified ousing preparative HPLC on reversed phase using linear gradient of MeOH in 0.1M aq. TEAB. The final deprotection was performed according to literature procedure [9]. After preparative HPLC on reversed phase using linear gradient of MeOH in 0.1M aq. TEAB and converting to ammonium salt by passing through a column of Dowex 50 in ammonia form desired completely deprotected product was obtained in 68% yield (260 mg, 0.35 mmol) as well as isobutyrylated sideproduct (determined by LCMS) in 1.6% yield (6.8 mg, 8.4 μmol).

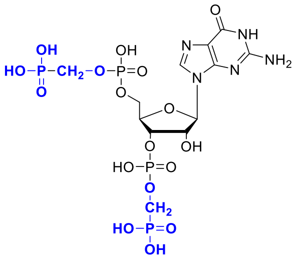

DR-4250

^{1}H NMR (500.0 MHz, D_2O, ref(dioxane) = 3.75 ppm): 3.89, 3.93 (2 × ddd, 2 × 1H, J_{gem} = 13.0, $J_{H,P}$ = 9.2, 4.9, OCH_2P); 3.96 (ddd, 1H, J_{gem} = 13.3, $J_{H,P}$ = 9.1, 4.9, OC$\mathbf{H_b}$H$_a$P); 4.02 (ddd, 1H, J_{gem} = 13.3, $J_{H,P}$ = 9.4, 5.5, OCH$_b$$\mathbf{H_a}$P); 4.18 (ddd, 1H, J_{gem} = 11.8, $J_{H,P}$ = 4.3, $J_{5'b,4'}$ = 3.2, H-5'b); 4.22 (ddd, 1H, J_{gem} = 12.0, $J_{H,P}$ = 4.8, $J_{5'a,4'}$ = 2.8, H-5'a); 4.59 (ddd, 1H, $J_{4',3'}$ = 3.4, $J_{4',5'}$ = 3.2, 2.8, H-4'); 4.85 (ddd, 1H, $J_{H,P}$ = 7.7, $J_{3',2'}$ = 5.1, $J_{3',4'}$ = 3.4, H-3'); 4.90 (ddd, 1H, $J_{2',1'}$ = 5.8, $J_{2',3'}$ = 5.8, $J_{H,P}$ = 1.3, H-2'); 6.07 (d, 1H, $J_{1',2'}$ = 5.8, H-1'); 8.63 (s, 1H, H-8).

^{13}C NMR (125.7 MHz, D_2O, ref(dioxane) = 69.30 ppm): 63.87 (dd, $J_{C,P}$ = 160.5, 7.5, CH_2P); 64.00 (dd, $J_{C,P}$ = 160.3, 7.7, CH_2P); 67.44 (d, $J_{C,P}$ = 5.1, CH_2-5'); 75.99 (d, $J_{C,P}$ = 4.8, CH-2'); 77.01 (d, $J_{C,P}$ = 5.3, CH-3'); 86.22 (dd, $J_{C,P}$ = 8.8, 3.8, CH-4'); 90.57 (CH-1'); 114.83 (C-5); 139.39 (CH-8); 153.58 (C-4); 157.50 (C-2); 159.75 (C-6).

$^{31}P\{^1H\}$ NMR (202.3 MHz, D_2O): 1.15 (d, $J_{P,P}$ = 37.1, phosphate); 1.68 (d, $J_{P,P}$ = 37.5, phosphate); 15.45 (d, $J_{P,P}$ = 37.5, phosphonate); 15.50 (d, $J_{P,P}$ = 37.1, phosphonate).

IR ν_{max}(KBr) 3540 (m, br, sh), 3423 (s, br), 3126 (m, vbr), 1696 (s), 1662 (s, sh), 1645 (s), 1534 (m), 1489 (m), 1400 (s), 1239 (s, br), 1100 (s), 1060 (vs, br), 932 (m, br), 781 (m), 689 (m).

HRMS (ESI+) for $C_{12}H_{21}N_5O_{17}P_4Na$ $(M+Na)^+$: calcd 653.97751, found 653.97782.

The compounds **DR-4239** and **DR-4238** were characterized only by means of LC-MS (data not shown).

Synthesis of phosphoramidite precursor:
benzyl ((bis(benzyloxy)phosphoryl)methyl) diisopropylphosphoramidite (DR-6200)

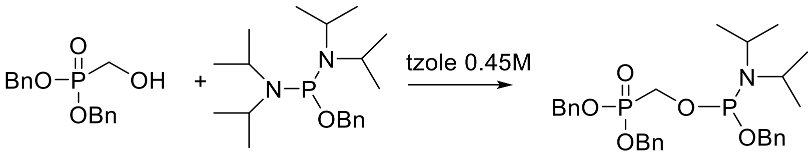

Scheme 2

Tetrazole (13 ml, 5.9 mmol, 0.45M in MeCN) was added to the solution of dibenzyl hydroxymethanphosphonate (1.7 g, 5.8 mmol) and benzyl-*N*,*N*,*N'*,*N'*-tetraisopropylphosphorodiamidite (2 g, 5.9 mmol) in MeCN (50 ml) under argon atmosphere. The reaction mixture was stirred for 1 h and evaporated under reduced pressure. The title compound was obtained by chromatography on silica gel using linear gradient of ethyl acetate in toluene in 86% yield (2.65 g, 5 mmol).

^1H NMR (400 MHz, C_6D6) R (400 MHz, J = 7.1, 1.2 Hz, 0H), 1.03 – 1.12 (m, 11H), 1.12 – 1.27 (m, 1H), 3.45 – 3.64 (m, 2H), 3.86 – 4.01 (m, 1H), 4.00 – 4.13 (m, 1H), 4.69 (dd, J = 12.6, 8.2 Hz, 1H), 4.77 (dd, J = 12.6, 8.3 Hz, 1H), 4.93 – 5.11 (m, 4H), 6.97 – 7.17 (m, 9H), 7.25 (dt, J = 7.9, 1.9 Hz, 4H), 7.28 – 7.36 (m, 2H).

^{13}C NMR (101 MHz, Benzene-d_6) NMR (101 MHz, Benzene$J$ = 10.7 Hz), 127.57, 67.45 (d, J = 6.3 Hz), 65.94 (d, J = 17.7 Hz), 58.60 (d, J = 15.1 Hz), 56.90 (d, J = 15.1 Hz), 24.37.

^{31}P NMR (162 MHz, Benzene-d_6) δ 154.31 (d, J = 37.0 Hz), 25.11 (d, J = 37.2 Hz).

HRMS (ESI+) for $C_{11}H_{16}N_6O_5P$ $(M+H)^+$: calcd, found.

2'-Deoxyguanosine 3',5'-di(phosphonomethoxyphosphate) DR-6241A (entry 7)

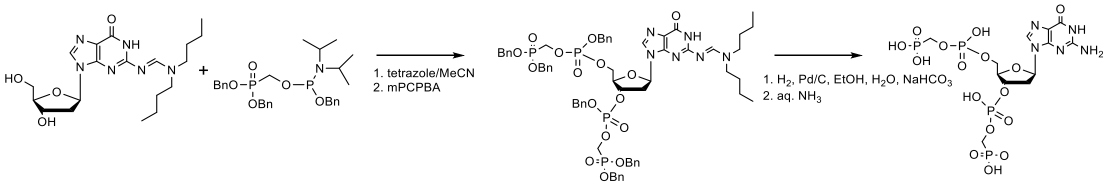

Scheme 3

Tetrazole (0.45 M in MeCN, 25 ml g, 11.3 mmol) was added to the solution of 2-*N*-dibutylaminomethylen-2'-deoxyguanosine (0.92 g, 2.26 mmol) and benzyl ((bis(benzyloxy)phosphoryl)methyl) diisopropylphosphoramidite (3 g, 5.67 mmol) in MeCN (20 ml) under argon atmosphere. The reaction mixture was stirred for 40 min (followed by TLC in 10% EtOH in $CHCl_3$) and mCPBA (1 g, 6 mmol) was added. The reaction mixture was stirred additional 20 min and evaporated under reduced pressure. The fully protected intermediate was obtained by column chromatography on silica gel using linear gradient of ethanol in $CHCl_3$ as an unseparable mixture of diastereomers (characterized only by means of LCMS) in 84% yield (2.47 g, 1.9 mmol). The intermediate was dissolved in the mixture of EtOH (50 ml) and water (50 ml), $NaHCO_3$ (0.96 g, 11.4 mmol) and Pd/C (0.2 g) were added. The mixture was then hydrogenated overnight at the pressure of 100 kPa. After filtration over cellite the mixture was concentrated in vacuo and purified ousing preparative HPLC on reversed phase using linear gradient of MeOH in 0.1M aq. TEAB. The final deprotection was performed as per Schattenkerk et al. (1985)[9] (overnight treatment with conc. aq. NH_3). After preparative HPLC on reversed phase using linear gradient of MeOH in 0.1M aq. TEAB and converting to ammonium salt by passing through a column of Dowex 50 in ammonia form desired product was obtained in 21% overall yield (308 mg, 0.4 mmol).

^{1}H NMR (500.0 MHz, D_2O): 2.81 (ddd, 1H, J_{gem} = 14.2, $J_{2'b,1'}$ = 6.4, $J_{2'b,3'}$ = 3.2, H-2'b); 2.85 (ddd, 1H, J_{gem} = 14.2, $J_{2'a,1'}$ = 7.6, $J_{2'a,3'}$ = 5.2, H-2'a); 3.80 – 3.99 (m, 4H, CH_2P); 4.13 (ddd, 1H, J_{gem} = 12.0, $J_{H,P}$ = 4.9, $J_{5'b,4'}$ = 3.6, H-5'b); 4.15 (ddd, 1H, J_{gem} = 12.0, $J_{H,P}$ = 4.5, $J_{5'a,4'}$ = 3.6, H-5'a); 4.35 (tdd, 1H, $J_{4',5'}$ = 3.6, $J_{4',3'}$ = 3.2, $J_{H,P}$ = 2.1, H-4'); 5.03 (tdd, 1H, $J_{H,P}$ = 5.2, $J_{3',2'}$ = 5.2, 3.6, $J_{3',4'}$ = 3.2, H-3'); 6.40 (dd, 1H, $J_{1',2'}$ = 7.6, 6.4, H-1'); 8.47 (s, 1H).

^{13}C NMR (125.7 MHz, D_2O): 41.15 (d, $J_{C,P}$ = 3.4, CH_2-2'); 63.91, 63.97 (2 × dd, $J_{C,P}$ = 160.5, 7.5, CH_2P); 68.03 (d, $J_{C,P}$ = 5.3, CH_2-5'); 78.91 (d, $J_{C,P}$ = 5.3, CH-3'); 87.47 (CH-1'); 88.04 (dd, $J_{C,P}$ = 8.7, 6.3, CH-4'); 115.83 (C-5); 139.51 (CH-8); 153.41 (C-4); 157.18 (C-2); 160.16 (C-6).

^{31}P{^{1}H} NMR (202.3 MHz, D_2O): 0.84, 1.69 (2 × d, 2 × 1P, $J_{P,P}$ = 38.0, phosphate); 15.30 (d, 2P, $J_{P,P}$ = 38.0, phosphonate).

IR ν_{max}(KBr) 3414 (s, br), 3200 (vs, vbr, sh), 3041 (vs, vbr), 2350 (w, vbr, sh), 2180 (w, br, sh), 1875 (w, vbr), 1721 (s, sh), 1691 (s), 1645 (sh), 1610 (m), 1576 (m, sh), 1532 (w), 1478 (m, sh), 1405 (m, sh), 1216 (s, br), 1080 (s, br, sh), 1047 (s, br), 1015 (s, br, sh), 919 (m, br), 780 (m), 695 (w), 641 (w).

HRMS (ESI-) for $C_{12}H_{20}N_5O_{16}P_4$ $(M-H)^-$: calcd 613.98610, found 613.98640.

Guanosine 5'-phosphonomethoxyphosphate DR-6222 (entry 8)

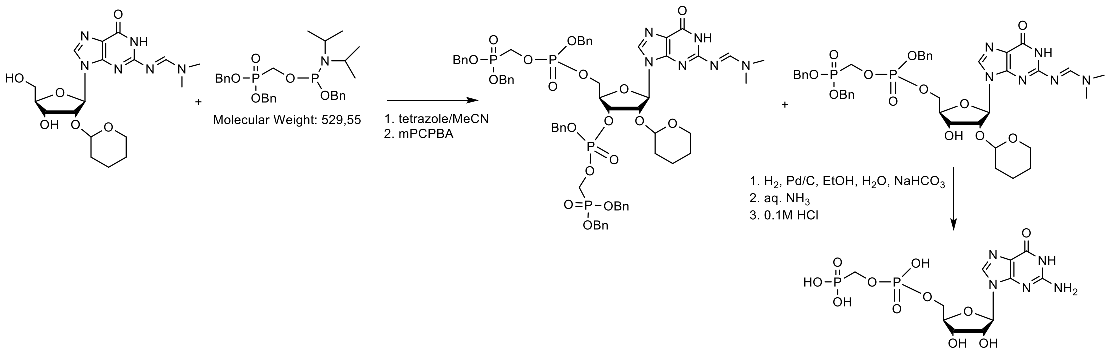

Scheme 4

Tetrazole (0.45M in MeCN, 35 ml g, 15.75 mmol) was added to the solution of 2-*N*-dimethylaminomethylen-2'-deoxyguanosine (1 g, 2.37 mmol) and benzyl ((bis(benzyloxy)phosphoryl)methyl) diisopropylphosphoramidite (1.6 g, 3 mmol) in MeCN (30 ml) under argon atmosphere. The reaction mixture was stirred for 40 min (followed by TLC in 10% EtOH in $CHCl_3$) and mCPBA (1 g, 6 mmol) was added. The reaction mixture was stirred additional 20 min and evaporated under reduced pressure. The desired monosubstituted intermediate was obtained by column chromatography on silica gel using linear gradient of ethanol in $CHCl_3$ as an unseparable mixture of diastereomers (characterized only by means of LCMS) in 28% yield (0.58 g, 0.67 mmol) as well as bissubstituted derivative in 26% yield (0.82 g, 0.63 mmol).

The monosubstituted intermediate (0.58 g, 0.67 mmol) was dissolved in the mixture of EtOH (25 ml) and water (25 ml), $NaHCO_3$ (0.23 g, 2.72 mmol) and Pd/C (0.2 g) were added. The mixture was then hydrogenated overnight at the pressure of 100 kPa. After filtration over cellite the mixture was concentrated in vacuo and purified ousing preparative HPLC on reversed phase using linear gradient of MeOH in 0.1M aq. TEAB. The final deprotection was performed according to literature procedure [9] (overnight treatment with conc. aq. NH_3 followed by treatment with 0.1M aq. HCl). After preparative HPLC on reversed phase using linear gradient of MeOH in 0.1M aq. TEAB and converting to ammonium salt by passing through a column of Dowex 50 in ammonia form desired product was obtained in 43% overall yield (149 mg, 0.29 mmol).

^{1}H NMR (500.0 MHz, D_2O): 3.86 – 3.95 (m, 2H, CH_2P); 4.15 (ddd, 1H, J_{gem} = 11.6, $J_{H,P}$ = 5.4, $J_{5'b,4'}$ = 3.4, H-5'b); 4.18 (ddd, 1H, J_{gem} = 11.6, $J_{H,P}$ = 4.8, $J_{5'a,4'}$ = 3.1, H-5'a); 4.35 (dddd, 1H, $J_{4',3'}$ = 4.0, $J_{4',5'}$ = 3.4, 3.1, $J_{H,P}$ = 2.0, H-4'); 4.50 (dd, 1H, $J_{3',2'}$ = 5.2, $J_{3',4'}$ = 4.0, H-3'); 4.73 (dd, 1H, $J_{2',1'}$ = 5.5, $J_{2',3'}$ = 5.2, H-2'); 5.93 (d, 1H, $J_{1',2'}$ = 5.5, H-1'); 8.18 (s, 1H).

^{13}C NMR (125.7 MHz, D_2O): 63.96 (dd, $J_{C,P}$ = 160.5, 7.6, CH_2P); 67.48 (d, $J_{C,P}$ = 5.3, CH_2-5'); 72.86 (CH-3'); 76.54 (CH-2'); 86.36 (d, $J_{C,P}$ = 8.7, CH-4'); 89.97 (CH-1'); 117.96 (C-5); 139.87 (CH-8); 154.12 (C-4); 156.77 (C-2); 161.11 (C-6).

^{31}P{^{1}H} NMR (202.3 MHz, D_2O): 1.81 (d, $J_{P,P}$ = 37.5, phosphate); 15.21 (d, $J_{P,P}$ = 37.5, phosphonate).

IR ν_{max}(KBr) 3133 (vs, vbr), 2775 (m, br, sh), 2360 (w, vbr, sh), 1693 (vs), 1658 (m, sh), 1614 (m), 1580 (w), 1533 (m), 1483 (m), 1403 (m), 1219 (s, br), 1178

(m), 1084 (s, br), 1039 (s, br), 992 (m, sh), 909 (m), 809 (m), 779 (m), 691 (w), 639 (w).

HRMS (ESI-) for $C_{11}H_{16}N_5O_{11}P_2$ $(M\text{-}H)^-$: calcd 456.03270, found 456.03220.

2'-Deoxyguanosine 3',5'-di-*O*-(methylenephosphonate) DR-5799C (entry 9)

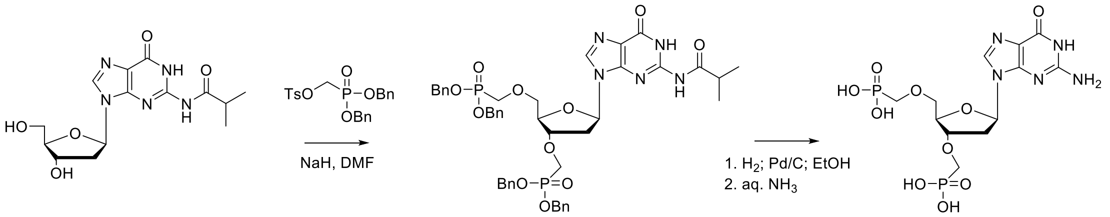

Scheme 5

Sodium hydride (45 mg, 1.12 mmol) was added to the solution of 2-*N*-isobutyryl-2'-deoxyguanosine (94 mg, 0,28 mmol) and dibenzyl tosyloxymethanephosphonate (0.31 g, 0.69 mmol) in DMF (3 ml) at rt under argon atmosphere. The reaction mixture was stirring overnight. The reaction mixture was cooled to 0 °C and acetic acid (64 µl, 1.12 mmol) was added. The reaction mixture was stirred additional 10 min and concentrated in vacuo. Tetrabenzyl ester intermediate was obtained by column chromatography on silica gel using linear gradient of ethanol in chloroform and without characterization dissolved in EtOH (10 ml). Pd/C (50 mg) was added and the reaction mixture was hydrogenated at 1 atm of H_2 overnight. The suspension was filtered, the filtrate concentrated in vacuo. The final deprotection was performed according to literature procedure [9] (overnight treatment with conc. aq. NH_3 followed by treatment with 0.1M aq. HCl). The final product was obtained by preparative HPLC on reversed phase using linear gradient of methanol in 0.1% aq. TEAB in 25% overall yield (115 mg, 0.21 mmol) after conversion to ammonium salt by passing through small column of Dowex 50 in NH_4^+ form.

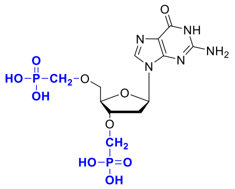

^{1}H NMR (500.0 MHz, D_2O, ref(dioxane) = 3.75 ppm): 2.71 (ddd, 1H, J_{gem} = 14.2, $J_{2'b,1'}$ = 6.4, $J_{2'b,3'}$ = 2.9, H-2'b); 2.77 (ddd, 1H, J_{gem} = 14.2, $J_{2'a,1'}$ = 7.8, $J_{2'a,3'}$ = 5.8, H-2'b); 3.63 – 3.76 (m, 4H, CH_2P); 3.79 (dd, 1H, J_{gem} = 11.0, $J_{5'b,4'}$ = 4.8, H-5'b); 3.85 (dd, 1H, J_{gem} = 11.0, $J_{5'a,4'}$ = 3.4, H-5'b); 4.41 (ddd, 1H, $J_{4',5'}$ = 4.8, 3.4, $J_{4',3'}$ = 2.4, H-4'); 4.48 (ddd, 1H, $J_{3',2'}$ = 5.8, 2.9, $J_{3',4'}$ = 2.4, H-3'); 6.33 (dd, 1H, $J_{1',2'}$ = 7.8, 6.4, H-1'); 8.47 (s, 1H, H-8).

^{13}C NMR (125.7 MHz, D_2O, ref(dioxane) = 69.3 ppm): 39.29 (CH_2-2'); 67.95 (d, $J_{C,P}$ = 157.4, CH_2P); 70.10 (d, $J_{C,P}$ = 156.8, CH_2P); 75.50 (d, $J_{C,P}$ = 11.8, CH_2-5'); 84.50 (d, $J_{C,P}$ = 12.3, CH-3'); 86.73 (CH-4'); 87.22 (CH-1'); 116.18 (C-5); 139.91 (CH-8); 153.51 (C-4); 157.09 (C-2); 160.39 (C-6).

^{31}P{^{1}H} NMR (202.3 MHz, D_2O): 16.24, 16.27.

HRMS (ESI-) for $C_{12}H_{18}N_5O_{10}P_2$ $(M\text{-}H)^-$: calcd 454.05289, found 454.05260.

Guanosine 3',5'-di-*O*-(methylenephosphonate) DR-6331 (entry 10)

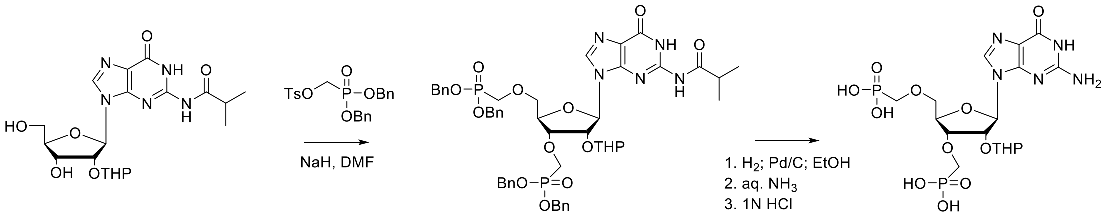

Scheme 6

Sodium hydride (270 mg, 6.72 mmol) was added to the solution of 2-*N*-isobutyryl-2'-*O*-tetrahydropyranylguanosine (0.37 g, 0.85 mmol) and dibenzyl tosyloxymethanephosphonate (1.86 g, 4.14 mmol) in DMF (10 ml) at rt under argon atmosphere. The reaction mixture was stirring overnight. The reaction mixture was cooled to 0 °C and acetic acid (0.38 ml, 6.72 mmol) was added. The reaction mixture was stirred additional 10 min and concentrated in vacuo. Tetrabenzyl ester intermediate was obtained by column chromatography on silica gel using linear gradient of ethanol in chloroform and without characterization dissolved in EtOH (10 ml). Pd/C (50 mg) was added and the reaction mixture was hydrogenated at 1 atm of H_2 overnight. The suspension was filtered, the filtrate concentrated in vacuo and dissolved in conc. aq. NH_3. The mixture was left aside overnight, concentrated, and the final product was obtained by preparative HPLC on reversed phase using linear gradient of methanol in 0.1% aq. TEAB in % overall yield (115 mg, 0.21 mmol) after conversion to ammonium salt by passing through small column of Dowex 50 in NH_4^+ form.

^{1}H NMR (500.0 MHz, D_2O): 3.70, 3.74 (2 × dd, 2 × 1H, J_{gem} = 13.4, $J_{H,P}$ = 8.6, CH_2P-5'); 3.79, 3.85 (2 × dd, 2 × 1H, J_{gem} = 13.1, $J_{H,P}$ = 9.1, CH_2P-3'); 3.85 (dd, 1H, J_{gem} = 11.2, $J_{5'b,4'}$ = 4.1, H-5'b); 3.92 (dd, 1H, J_{gem} = 11.2, $J_{5'a,4'}$ = 2.9, H-5'a); 4.34 (dd, 1H, $J_{3',2'}$ = 5.1, $J_{3',4'}$ = 3.9, H-3'); 4.46 (ddd, 1H, $J_{4',5'}$ = 4.1, 2.9, $J_{4',3'}$ = 3.9, H-4'); 4.86 (dd, 1H, $J_{2',1'}$ = 5.6, $J_{2',3'}$ = 5.1, H-2'); 5.97 (d, 1H, $J_{1',2'}$ = 5.6, H-1'); 8.35 (s, 1H).

^{13}C NMR (125.7 MHz, D_2O): 69.16 (d, $J_{C,P}$ = 156.5, CH_2P-3'); 70.14 (d, $J_{C,P}$ = 156.8, CH_2P-5'); 74.86 (d, $J_{C,P}$ = 12.0, CH_2-5'); 75.97 (CH-2'); 82.75 (d, $J_{C,P}$ = 11.6, CH-3'); 84.52 (CH-4'); 90.14 (CH-1'); 117.20 (C-5); 140.03 (CH-8); 154.02 (C-4); 156.95 (C-2); 160.81 (C-6).

^{31}P{^{1}H} NMR (202.3 MHz, D_2O): 15.40, 15.74.

IR ν_{max}(KBr) 3395 (m, br, sh), 3325 (m, br, sh), 3130 (vs, br), 3015 (s, sh), 2780 (m, br, sh), 2350 (w, vbr), 1692 (vs), 1653 (m, sh), 1612 (m), 1581 (w), 1533 (m), 1489 (m), 1401 (s), 1176 (m), 1140 (s, br), 1068 (s, br), 1041 (m, sh), 923 (m, br), 802 (w), 780 (w), 692 (w), 635 (w).

HRMS (ESI-) for $C_{12}H_{18}N_5O_{11}P_2$ $(M-H)^-$: calcd 470.04835, found 470.04855.

2-*N*-isobutyryl-2'-deoxyguanosine 3',5'-di-*O*-(2-phosphonoacetate) DR-5824A (entry 11)

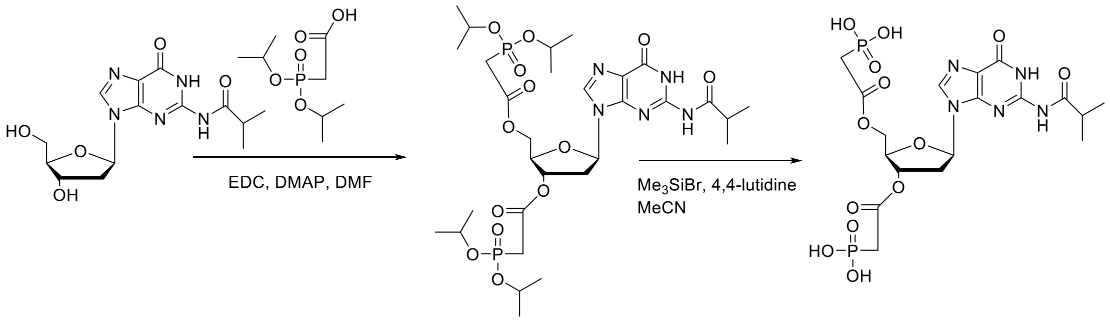

Scheme 7

EDC (1.4 g, 7.4 mmol) was added to the mixture of 2-*N*-isobutyryldeoxyguanosine (0.5 g, 1.48 mmol), diisopropyl 2-phosphonopropionic acid (1 g, 4.45 mmol), and DMAP (0.1 g, 0.74 mmol) in DMF (15 ml). The reaction mixture was stirred under argon atmosphere at 70 °C two days. The reaction mixture was concentrated in vacuo and tetraisopropyl ester intermediate was obtained by column chromatography on silica gel using linear gradient of ethanol in chloroform in 65% (0.73 g, 0.97 mmol) and used without further characterization (LCMS only). The intermediate (0.73 g, 0.97 mmol) was co-evaporated with MeCN (2x 10 ml) and dissolved in the same solvent (10 ml). 4,4-lutidine (1.13 ml, 9.7 mmol) and Me_3SiBr (0.9 ml, 6.79 mmol) were subsequently added under argon atmosphere. The reaction mixture was stirred under argon atmosphere at rt overnight. The reaction mixture was concentrated under reduced pressure, 0.2M TEAB (5 ml) and EtOH (10 ml) were added and the mixture was concentrated again under reduced pressure. The final product was obtained by preparative HPLC on reversed phase using linear gradient of methanol in 0.1% aq. TEAB in 57% yield (360 mg, 0.55 mmol) after conversion to ammonium salt by passing through column of Dowex 50 in NH_4^+ form (30 ml).

^{1}H NMR (500.0 MHz, D_2O, ref(dioxane) = 3.75 ppm): 1.23 (d, 6H, J_{vic} = 6.9, (C**H**$_3$)$_2$CH); 2.76 (ddd, 1H, J_{gem} =14.6, $J_{2'b,1'}$ = 6.2, $J_{2'b,1'}$ = 2.7, H-2'b); 2.80 (sep, 1H, J_{vic} = 6.9, C**H**(CH$_3$)$_2$); 2.82 (d, 2H, $J_{H,P}$ = 20.5, CH$_2$P-5'); 2.86 – 2.98 (m, 2H, CH$_2$P-3'); 3.09 (ddd, 1H, J_{gem} = 14.6, $J_{2'a,1'}$ = 7.9, $J_{2'a,3'}$ = 6.5, H-2'a); 4.43 (d, 2H, $J_{5',4'}$ = 4.5, H-5'); 4.57 (td, 1H, $J_{4',5'}$ = 4.5, $J_{4',3'}$ = 2.7, H-4'); 5.56 (dt, 1H, $J_{3',2'}$ = 6.5, 2.7, $J_{3',4'}$ = 2.7, H-3'); 6.48 (dd, 1H, $J_{1',2'}$ = 7.9, 6.2, H-1'); 8.25 (s, 1H, H-8).

^{13}C NMR (125.7 MHz, D_2O, ref(dioxane) = 69.3 ppm): 20.91 ((**C**H$_3$)$_2$CH); 38.51 (**C**H(CH$_3$)$_2$); 38.66 (CH$_2$-2'); 39.30 (d, $J_{C,P}$ = 118.1, CH$_2$P-5'); 39.60 (d, $J_{C,P}$ = 116.9, CH$_2$P-3'); 67.34 (CH$_2$-5'); 77.94 (CH-3'); 84.92 (CH-4'); 87.03 (CH-1'); 122.73 (C-5); 142.35 (CH-8); 150.65 (C-2); 152.07 (C-4); 160.11 (C-6); 172.92 (d, $J_{C,P}$ = 6.4, COO-3'); 173.09 (d, J = 6.4, COO-5'); 184.87 (CONHiPr).

^{31}P{^{1}H} NMR (202.3 MHz, D_2O): 12.46, 12.64.

IR ν_{max}(KBr) 3135 (s, vbr), 2978 (s), 2940 (s), 2878 (m), 1716 (vs), 1685 (vs), 1609 (s), 1565 (m), 1540 (w, sh), 1474 (m), 1403 (s), 1375 (w, sh), 1261 (s, br), 1212 (m, sh), 1190 (s), 1158 (s), 1106 (s), 1057 (s), 1031 (m, sh), 916 (m, br), 799 (w), 720 (w), 642 (w).

HRMS (ESI-) for $C_{18}H_{24}N_5O_{13}P_2$ $(M-H)^-$: calcd 580.08513, found 580.08531.

2-*N*-isobutyryl-2'-deoxyguanosine 3',5'-di-*O*-(3-phosphonopropionate) DR-5825A (entry 12)

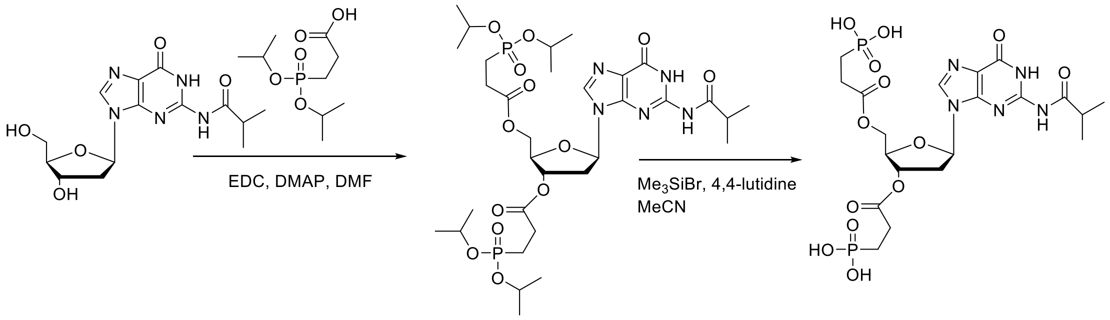

Scheme 8

EDC (1.4 g, 7.4 mmol) was added to the mixture of 2-*N*-isobutyryldeoxyguanosine (0.5 g, 1.48 mmol), diisopropyl 2-phosphonopropionic acid (1 g, 4.2 mmol), and DMAP (0.2 g, 1.6 mmol) in DMF (15 ml). The reaction mixture was stirred under argon atmosphere at 70 °C two days. The reaction mixture was concentrated in vacuo and tetraisopropyl ester intermediate was obtained by column chromatography on silica gel using linear gradient of ethanol in chloroform in 67% (0.77 g, 0.99 mmol) and used without further characterization (LCMS only). The intermediate (0.77 g, 0.99 mmol) was co-evaporated with MeCN (2x 15 ml) and dissolved in the same solvent (10 ml). 4,4-lutidine (1.15 ml, 9.9 mmol) and Me_3SiBr (1 ml, 7.9 mmol) were subsequently added under argon atmosphere. The reaction mixture was stirred under argon atmosphere at rt overnight. The reaction mixture was concentrated under reduced pressure, 0.2M TEAB (5 ml) and EtOH (10 ml) were added and the mixture was concentrated again under reduced pressure. The final product was obtained by preparative HPLC on reversed phase using linear gradient of methanol in 0.1% aq. TEAB in 40% yield (268.4 mg, 0.4 mmol) after conversion to ammonium salt by passing through column of Dowex 50 in NH_4^+ form (30 ml).

^{1}H NMR (600.1 MHz, D_2O, ref(dioxane) = 3.75 ppm): 1.225, 1.227 (2 × d, 2 × 3H, J_{vic} = 6.9, (C**H₃**)₂CH); 1.78 – 1.86 (m, 2H, PC**H₂**CH₂CO-5'); 1.90 – 1.96 (m, 2H, PC**H₂**CH₂CO-3'); 2.51 – 2.63 (m, 2H, PCH₂C**H₂**CO-5'); 2.65 – 2.71 (m, 2H, PCH₂C**H₂**CO-3'); 2.77 (ddd, 1H, J_{gem} =14.5, $J_{2'b,1'}$ = 6.4, $J_{2'b,1'}$ = 2.9, H-2'b); 2.80 (sep, 1H, J_{vic} = 6.9, C**H**(CH₃)₂); 3.06 (ddd, 1H, J_{gem} = 14.5, $J_{2'a,1'}$ = 7.5, $J_{2'a,3'}$ = 6.6, H-2'a); 4.39 – 4.45 (m, 2H, H-5'); 4.54 (td, 1H, $J_{4',5'}$ = 4.7, $J_{4',3'}$ = 2.9, H-4'); 5.55 (dt, 1H, $J_{3',2'}$ = 6.6, 2.9, $J_{3',4'}$ = 2.9, H-3'); 6.45 (dd, 1H, $J_{1',2'}$ = 7.5, 6.4, H-1'); 8.21 (s, 1H, H-8).

^{13}C NMR (150.9 MHz, D_2O, ref(dioxane) = 69.3 ppm): 20.88, 20.92 ((**C**H₃)₂CH); 25.70, 25.74 (d, $J_{C,P}$ = 135.6, P**C**H₂CH₂CO-3',5'); 31.15 (d, $J_{C,P}$ = 3.0, PCH₂**C**H₂CO-5'); 31.40 (d, $J_{C,P}$ = 3.0, PCH₂**C**H₂CO-3'); 38.53 (**C**H(CH₃)₂); 38.60 (CH₂-2'); 67.05 (CH₂-5'); 77.69 (CH-3'); 84.99 (CH-4'); 87.23 (CH-1'); 122.66 (C-5); 142.15 (CH-8); 150.63 (C-2); 151.93 (C-4); 159.99 (C-6); 177.58 (d, $J_{C,P}$ = 18.0, COO-3'); 177.82 (d, J = 18.7, COO-5').

^{31}P{^{1}H} NMR (202.3 MHz, D_2O): 23.92, 24.04.

IR ν_{max}(KBr) 3206 (w, vbr), 2977 (w), 2939 (w), 2879 (w), 1736 (m), 1715 (m, sh), 1683 (m), 1608 (m), 1564 (m), 1540 (vw-m, sh), 1472 (m), 1403 (s), 1380 (m, sh), 1231 (s), 1101 (vs, br), 1062 (vs, vbr), 914 (m, br), 796 (m, br), 720 (vw), 645

(vw).

HRMS (ESI-) for $C_{20}H_{28}N_5O_{13}P_2$ $(M-H)^-$: calcd 608.11643, found 608.11545.

2'-Deoxyguanosine 3',5'-di-*O*-(2-phosphonoacetate) DR-5839A (entry 13)

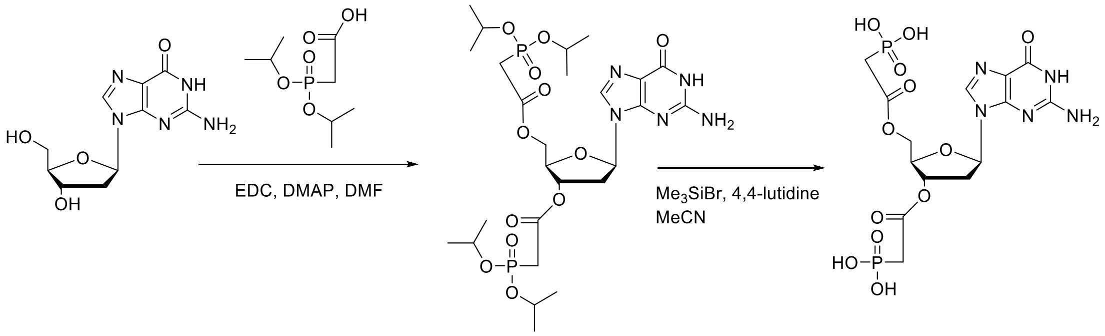

Scheme 9

EDC (3.2 g, 16.85 mmol) was added to the mixture of deoxyguanosine (0.9 g, 3.37 mmol), diisopropyl 2-phosphonoacetic acid (2 g, 8.42 mmol), and DMAP (0.2 g, 1.6 mmol) in DMF (35 ml). The reaction mixture was stirred under argon atmosphere at 70 °C two days. The reaction mixture was concentrated in vacuo and tetraisopropyl ester intermediate was obtained by column chromatography on silica gel using linear gradient of ethanol in chloroform in 51% (1.18 g, 1.73 mmol) and used without further characterization (LCMS only). The intermediate (1.18 g, 1.73 mmol) was co-evaporated with MeCN (2x 15 ml) and dissolved in the same solvent (10 ml). 4,4-lutidine (2 ml, 17.3 mmol) and Me_3SiBr (1.6 ml, 12.15 mmol) were subsequently added under argon atmosphere. The reaction mixture was stirred under argon atmosphere at rt overnight. The reaction mixture was concentrated under reduced pressure, 0.2M TEAB (5 ml) and EtOH (10 ml) were added and the mixture was concentrated again under reduced pressure. The final product was obtained by preparative HPLC on reversed phase using linear gradient of methanol in 0.1% aq. TEAB in 36% yield (360 mg, 0.62 mmol) after conversion to ammonium salt by passing through column of Dowex 50 in NH_4^+ form (30 ml).

^1H NMR (500.0 MHz, D_2O, ref(dioxane) = 3.75 ppm): 2.73 (ddd, 1H, J_{gem} =14.5, $J_{2'b,1'}$ = 6.1, $J_{2'b,1'}$ = 2.8, H-2'b); 2.81 – 2.90 (m, 2H, CH_2P-5'); 2.90 – 3.00 (m, 2H, CH_2P-3'); 2.99 (ddd, 1H, J_{gem} = 14.5, $J_{2'a,1'}$ = 7.9, $J_{2'a,3'}$ = 6.1, H-2'a); 4.41 (d, 2H, $J_{5',4'}$ = 4.3, H-5'); 4.53 (td, 1H, $J_{4',5'}$ = 4.3, $J_{4',3'}$ = 2.8, H-4'); 5.54 (dt, 1H, $J_{3',2'}$ = 6.1, 2.8, $J_{3',4'}$ = 2.8, H-3'); 6.27 (dd, 1H, $J_{1',2'}$ = 7.9, 6.1, H-1'); 8.01 (s, 1H, H-8).

^{13}C NMR (125.7 MHz, D_2O, ref(dioxane) = 69.3 ppm): 38.60 (CH_2-2'); 39.27 (d, $J_{C,P}$ = 118.4, CH_2P-5'); 39.57 (d, $J_{C,P}$ = 117.4, CH_2P-3'); 67.25 (CH_2-5'); 77.90 (CH-3'); 84.71 (CH-4'); 86.46 (CH-1'); 118.92 (C-5); 140.06 (CH-8); 154.06 (C-4); 156.47 (C-2); 161.43 (C-6); 172.82 (d, $J_{C,P}$ = 6.4, COO-3'); 173.03 (d, J = 6.3, COO-5').

$^{31}P\{^1H\}$ NMR (202.3 MHz, D_2O): 12.62, 12.79.

IR ν_{max}(KBr) 3411 (s, br), 3230 (s, br, sh), 3125 (s, vbr), 3010 (s, br, sh), 1719 (s), 1688 (vs), 1650 (s), 1609 (m), 1585 (w), 1534 (w), 1485 (m), 1413 (m), 1404 (m, sh), 1274 (m, br), 1166 (m, br), 1115 (m, br), 1056 (m, br), 1028 (m, sh), 919 (m, br), 803 (w), 782 (w), 729 (vw), 639 (w).

HRMS (ESI-) for $C_{14}H_{18}N_5O_{12}P_2$ $(M-H)^-$: calcd 510.04327, found 510.04295.

2'-Deoxyguanosine 3',5'-di-*O*-(3-phosphonopropionate) DR-5835 (entry 14)

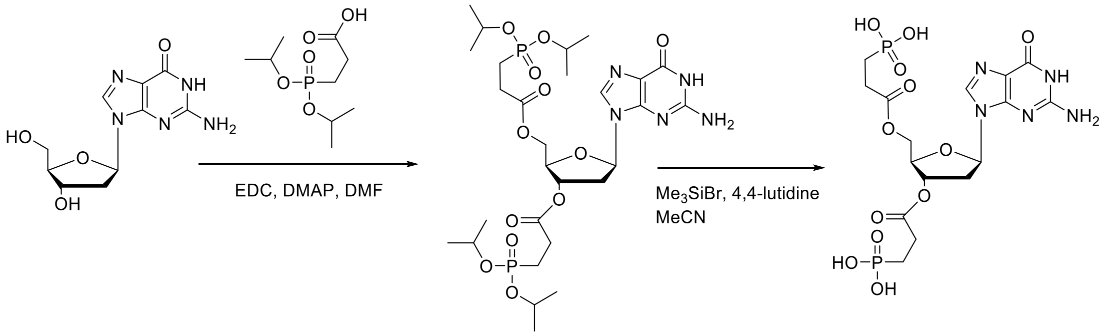

Scheme 10

EDC (4.8 g, 24.85 mmol) was added to the mixture of deoxyguanosine (0.95 g, 3.55 mmol), diisopropyl 3-phosphonopropionic acid (2.5 g, 10.7 mmol), and DMAP (0.4 g, 3.55 mmol) in DMF (30 ml). The reaction mixture was stirred under argon atmosphere at 70 °C two days. The reaction mixture was concentrated in vacuo and tetraisopropyl ester intermediate was obtained by column chromatography on silica gel using linear gradient of ethanol in chloroform in 80% (2.3 g, 2.83 mmol) and used without further characterization (LCMS only). The intermediate (0.54 g, 0,76 mmol) was co-evaporated with MeCN (2x 15 ml) and dissolved in the same solvent (10 ml). 4,4-lutidine (0.89 ml, 7.6 mmol) and Me_3SiBr (0.71 ml, 5.34 mmol) were subsequently added under argon atmosphere. The reaction mixture was stirred under argon atmosphere at rt overnight. The reaction mixture was concentrated under reduced pressure, 0.2M TEAB (5 ml) and EtOH (10 ml) were added and the mixture was concentrated again under reduced pressure. The final product was obtained by preparative HPLC on reversed phase using linear gradient of methanol in 0.1% aq. TEAB in 71% yield (330 mg, 0.45 mmol) after conversion to ammonium salt by passing through column of Dowex 50 in NH_4^+ form (30 ml).

^1H NMR (500.0 MHz, D_2O, ref(dioxane) = 3.75 ppm): 1.78 – 1.98 (m, 4H, PC**H**$_2$CH$_2$CO-3',5'); 2.50 – 2.71 (m, 4H, PCH$_2$C**H**$_2$CO-3',5'); 2.73 (ddd, 1H, J_{gem} =14.6, $J_{2'b,1'}$ = 6.3, $J_{2'b,1'}$ = 3.0, H-2'b); 2.99 (ddd, 1H, J_{gem} = 14.6, $J_{2'a,1'}$ = 7.4, $J_{2'a,3'}$ = 6.3, H-2'a); 4.35 – 4.42 (m, 2H, H-5'); 4.51 (td, 1H, $J_{4',5'}$ = 4.4, $J_{4',3'}$ = 3.0, H-4'); 5.52 (dt, 1H, $J_{3',2'}$ = 6.3, 3.0, $J_{3',4'}$ = 3.0, H-3'); 6.31 (dd, 1H, $J_{1',2'}$ = 7.3, 6.3, H-1'); 8.04 (s, 1H, H-8).

^{13}C NMR (125.7 MHz, D_2O, ref(dioxane) = 69.3 ppm): 25.73, 25.78 (d, $J_{C,P}$ = 135.5, P**C**H$_2$CH$_2$CO-3',5'); 31.16 (d, $J_{C,P}$ = 2.9, PCH$_2$**C**H$_2$CO-5'); 31.41 (d, $J_{C,P}$ = 3.0, PCH$_2$**C**H$_2$CO-3'); 38.54 (CH$_2$-2'); 66.91 (CH$_2$-5'); 77.54 (CH-3'); 84.82 (CH-4'); 86.79 (CH-1'); 118.67 (C-5); 139.99 (CH-8); 154.05 (C-4); 156.60 (C-2); 161.38 (C-6); 177.58 (d, $J_{C,P}$ = 17.9, COO-3'); 177.82 (d, J = 18.8, COO-5').

$^{31}P\{^1H\}$ NMR (202.3 MHz, D_2O): 23.83, 23.99.

IR ν_{max}(KBr) 3421 (vs, br), 3220 (vs, vbr, sh), 3127 (vs, vbr), 3048 (s, vbr, sh), 2843 (s, sh), 1729 (s, br), 1688 (vs), 1650 (s), 1610 (m), 1583 (m), 1534 (m), 1485 (m), 1420 (m), 1405 (m, sh), 1241 (m), 1172 (m, sh), 1128 (m, br), 1049 (m,

br), 909 (m, br), 799 (w), 781 (w), 728 (vw), 639 (w).

HRMS (ESI-) for $C_{16}H_{22}N_5O_{12}P_2$ $(M-H)^-$: calcd 538.07457, found 538.07458.

2'-Deoxyadenosine 3',5'-di-*O*-(3-phosphonopropionate) DR-5836 (entry 15)

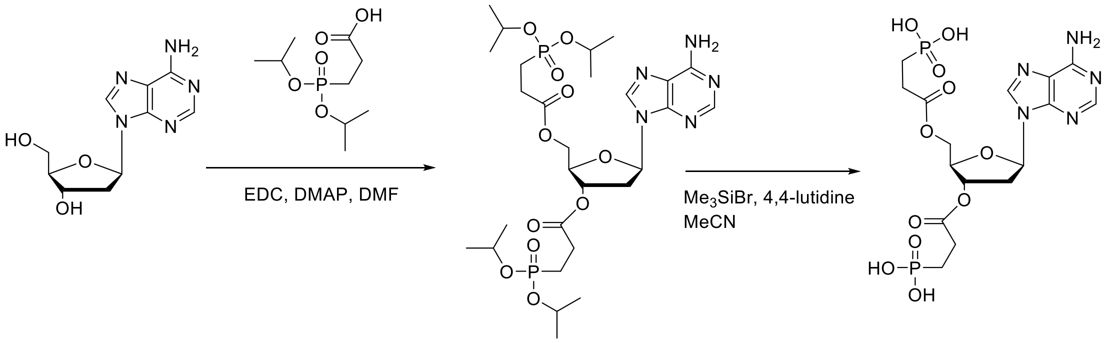

Scheme 11

EDC (7.8 g, 41 mmol) was added to the mixture of deoxyadenosine (1.58 g, 5.86 mmol), Diisopropyl 3-phosphonopropionic acid (4.19 g, 17.6 mmol), and DMAP (0.71 g, 5.86 mmol) in DMF (50 ml). The reaction mixture was stirred under argon atmosphere at 70 °C two days. The reaction mixture was concentrated in vacuo and tetraisopropyl ester intermediate was obtained by column chromatography on silica gel using linear gradient of ethanol in chloroform in 38% (1.53 g, 2.21 mmol) and used without further characterization (LCMS only). It was co-evaporated with MeCN (2x 20 ml) and dissolved in the same solvent (25 ml). 4,4-lutidine (2.6 ml, 22.1 mmol) and Me_3SiBr (2 ml, 15.48 mmol) were subsequently added under argon atmosphere. The reaction mixture was stirred under argon atmosphere at rt overnight. The reaction mixture was concentrated under reduced pressure, 0.2M TEAB (5 ml) and EtOH (10 ml) were added and the mixture was concentrated again under reduced pressure. The final product was obtained by preparative HPLC on reversed phase using linear gradient of methanol in 0.1% aq. TEAB in 20% overall yield (265 mg, 0.45 mmol) after conversion to ammonium salt by passing through column of Dowex 50 in NH_4^+ form (50 ml).

^{1}H NMR (500.0 MHz, D_2O, ref(dioxane) = 3.75 ppm): 1.75 – 1.85 (m, 2H, PC**H$_2$**CH$_2$CO-5'); 1.89 – 2.01 (m, 2H, PC**H$_2$**CH$_2$CO-3'); 2.43 – 2.61 (m, 2H, PCH$_2$C**H$_2$**CO-5'); 2.65 – 2.75 (m, 2H, PCH$_2$C**H$_2$**CO-3'); 2.81 (ddd, 1H, J_{gem} =14.5, $J_{2'b,1'}$ = 6.3, $J_{2'b,1'}$ = 3.2, H-2'b); 3.03 (ddd, 1H, J_{gem} = 14.5, $J_{2'a,1'}$ = 7.3, $J_{2'a,3'}$ = 6.5, H-2'a); 4.38 (d, 2H, $J_{5',4'}$ = 4.1, H-5'); 4.53 (td, 1H, $J_{4',5'}$ = 4.1, $J_{4',3'}$ = 3.2, H-4'); 5.54 (dt, 1H, $J_{3',2'}$ = 6.5, 3.2, $J_{3',4'}$ = 3.2, H-3'); 6.46 (dd, 1H, $J_{1',2'}$ = 7.3, 6.2, H-1'); 8.34 (s, 1H, H-8).

^{13}C NMR (125.7 MHz, D_2O, ref(dioxane) = 69.3 ppm): 25.76 (d, $J_{C,P}$ = 135.5, P**C**H$_2$CH$_2$CO-5'); 25.78 (d, $J_{C,P}$ = 135.5, P**C**H$_2$CH$_2$CO-3'); 31.17 (d, $J_{C,P}$ = 2.8, PCH$_2$**C**H$_2$CO-5'); 31.42 (d, $J_{C,P}$ = 3.0, PCH$_2$**C**H$_2$CO-3'); 38.89 (CH$_2$-2'); 66.84 (CH$_2$-5'); 77.40 (CH-3'); 84.97 (CH-4'); 86.95 (CH-1'); 121.44 (C-5); 142.78 (CH-8); 151.30 (C-4); 154.23 (CH-2); 157.35 (C-6); 177.60 (d, $J_{C,P}$ = 18.0, COO-3'); 177.76 (d, J = 19.1, COO-5').

^{31}P{^{1}H} NMR (202.3 MHz, D_2O): 23.69, 23.90.

IR ν_{max}(KBr) 3425 (vs, br), 3190 (vs, vbr), 3035 (s, vbr), 2924 (s), 2854 (s), 1737

(s), 1646 (s), 1607 (m), 1577 (m), 1507 (w, sh), 1478 (m), 1422 (m), 1334 (w), 1297 (w), 1241 (s), 1132 (s), 1048 (s, br), 901 (m, br), 798 (w), 726 (w), 646 (w).

HRMS (ESI-) for $C_{14}H_{18}N_5O_{11}P_2$ (M-H)$^+$: calcd 494.04780, found 494.04706.

Relacin (entry 16) was prepared according to [10].

Deisobutyrylated Relacin DR-DiBuRel (entry 17)

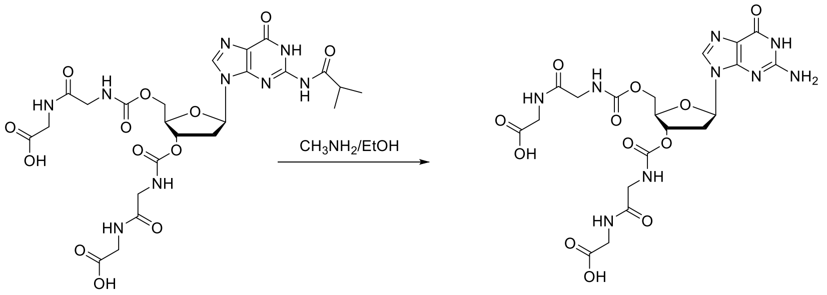

Scheme 12

Relacin was treated with 2M ethanolic methylamine (10 ml/mmol). Progress of the reaction was carefully monitored using HPLC. After complet conversion the desired deprotected relacin by preparative HPLC on reversed phase using linear gradient of MeOH in 0.1M aq. TEAB affording, after converting to ammonium salt by passing through a column of Dowex 50 in NH_4^+ form, title product in almost quantitative yield.

^{1}H NMR (500.0 MHz, D_2O): 2.71 (ddd, 1H, J_{gem} = 14.4, $J_{2'b,1'}$ = 6.3, $J_{2'b,3'}$ = 3.2, H-2'b); 2.94 (ddd, 1H, J_{gem} = 14.4, $J_{2'a,1'}$ = 7.7, $J_{2'a,3'}$ = 6.3, H-2'); 3.77 (d, 1H, J_{gem} = 17.4, CH$_a$**H$_b$**N); 3.82 (s, 2H, CH_2N); 3.83 (d, 1H, J_{gem} = 17.4, C**H$_a$**H$_b$N); 3.83, 3.93 (2 × s, 2 × 2H, CH_2N); 4.31 (dd, 1H, J_{gem} = 11.8, $J_{5'b,4'}$ = 4.8, H-5'b); 4.43 (dd, 1H, J_{gem} = 11.8, $J_{5'a,4'}$ = 3.5, H-5'b); 4.46 (ddd, 1H, $J_{4',5'}$ = 4.8, 3.5, $J_{4',3'}$ = 2.6, H-4'); 5.44 (ddd, 1H, $J_{3',2'}$ = 6.3, 3.2, $J_{3',4'}$ = 2.6, H-3'); 6.29 (dd, 1H, $J_{1',2'}$ = 7.7, 6.3, H-1'); 7.97 (s, 1H, H-8).

^{13}C NMR (125.7 MHz, D_2O): 38.73 (CH_2-2'); 45.40, 45.50, 46.30, 46.32 (CH_2N); 67.13 (CH_2-5'); 78.04 (CH-3'); 85.31 (CH-4'); 86.64 (CH-1'); 119.01 (C-5); 140.15 (CH-8); 154.18 (C-4); 156.54 (C-2); 160.33, 160.65 (OCONH); 161.59 (C-6); 174.74 (CONH); 178.55, 178.70 (COOH).

HRMS (ESI-) for $C_{20}H_{24}N_9O_{12}$ (M-H)$^-$: calcd 582.15444, found 582.15420.

Thio-DeiBu-Relacin DR-5732 (entry 18)

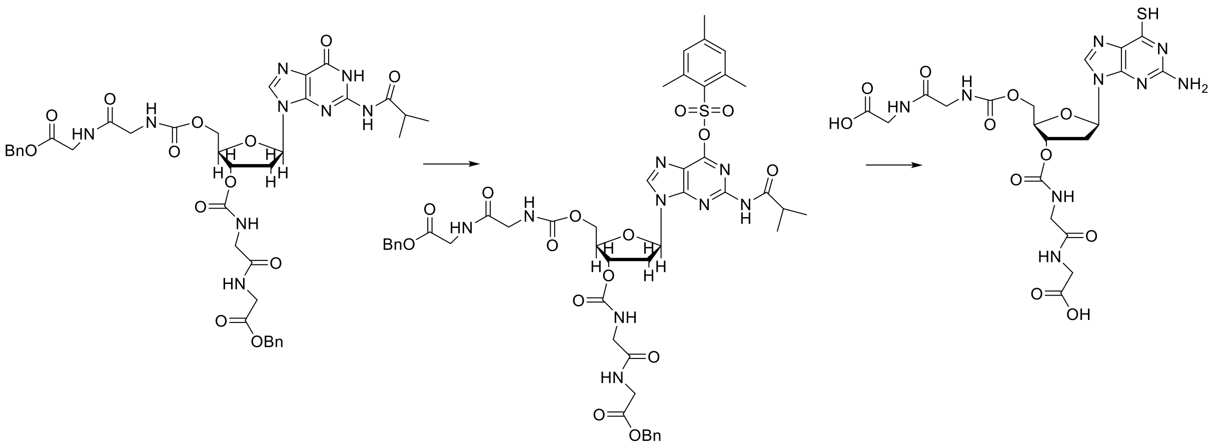

Scheme 13

Mesitylensulfonyl chloride was added to the mixture of dibenzyl-Relacin [10] (0.64 g, 0.77 mmol), triethylamine (0.22 ml, 1.6 mmol) and DMAP (52 mg, 0,43 mmol) in DCM (10 ml). The reaction mixture was stirred at rt under argon atmosphere overnight. The mixture was concentrated under reduced pressure and intermediate was obtained by column chromatography on silica gel using linear gradient of ethanol in chloroform in 30% yield (0.23 g, 0.23 mmol) that was without further characterization used in next step consisting in stirring with NaHS (0.2g, 2,26 mmol) in MeOH (5 ml) at 60 °C for 3h and then 48h at rt. The solvent was removed in vacuo the reaction mixture was purified on silica gel by fast linear gradient of ethanol in chloroform. Obtained intermediate was without further characterization (only LCMS) dissolved in 0.1M K_2CO_3 in water/MeOH (1:1) and stirred at rt for 1 h. pH of the reaction mixture was adjusted with AcOH to 7, end concentrated under reduced pressure. The final product was obtained by preparative HPLC on reversed phase using linear gradient of MeOH in 0.1M aq. TEAB affording, after converting to ammonium salt by passing through a column of Dowex 50 in NH_4^+ form, title product in 19% overall yield (26 mg, 0,043 mmol).

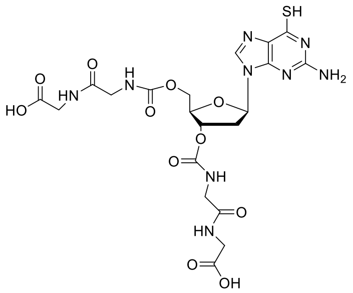

^{1}H NMR (500.0 MHz, D_2O): 2.72 (ddd, 1H, J_{gem} = 14.5, $J_{2'b,1'}$ = 6.4, $J_{2'b,3'}$ = 3.4, H-2'b); 2.97 (ddd, 1H, J_{gem} = 14.5, $J_{2'a,1'}$ = 7.2, $J_{2'a,3'}$ = 6.3, H-2'); 3.76, 3.81 (2 × d, 2 × 1H, J_{gem} = 17.4, CH_2N); 3.81, 3.82, 3.93 (3 × s, 3 × 2H, CH_2N); 4.31 (dd, 1H, J_{gem} = 11.7, $J_{5'b,4'}$ = 4.7, H-5'b); 4.43 (dd, 1H, J_{gem} = 11.7, $J_{5'a,4'}$ = 3.4, H-5'b); 4.46 (dt, 1H, $J_{4',5'}$ = 4.7, 3.4, $J_{4',3'}$ = 3.4, H-4'); 5.45 (dt, 1H, $J_{3',2'}$ = 6.3, 3.4, $J_{3',4'}$ = 3.4, H-3'); 6.29 (dd, 1H, $J_{1',2'}$ = 7.2, 6.4, H-1'); 8.09 (s, 1H, H-8).

^{13}C NMR (125.7 MHz, D_2O): 38.58 (CH_2-2'); 45.59, 45.66, 46.32, 46.33 (CH_2N); 67.07 (CH_2-5'); 77.97 (CH-3'); 85.35 (CH-4'); 86.90 (CH-1'); 131.23 (C-5); 142.93

(CH-8); 150.51 (C-4); 156.04 (C-2); 160.31, 160.62 (OCONH); 174.62, 174.64 (CONH); 176.47 (C-6); 178.80, 178.92 (COOH).

HRMS (ESI+) for $C_{20}H_{24}N_{9}O_{11}S$ $(M+H)^{+}$: calcd 598.13160, found 598.13155.

Azanucleotides

All azanucleotides were prepared according to general synthetic scheme 1S. In the first step a nucleobase has been attached to appropriately protected iminosugar (nitrogen containing heterocycle). Subsequently a phosphonic function has been attached followed by final deprotection.

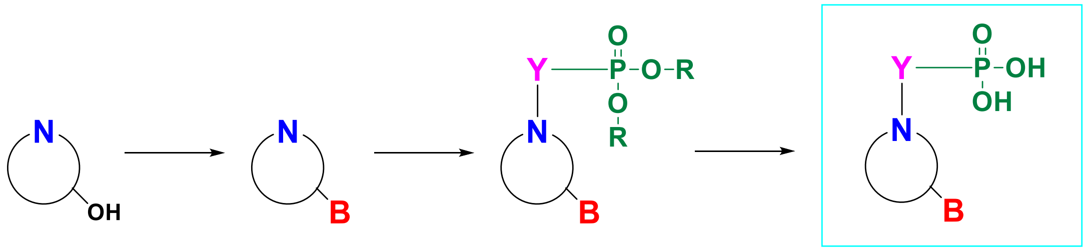

Scheme 14

Piperidine phosphonates (entries 19-31)

Piperidine phosphonates were prepared by already developed phosphonylation methods[11-16][ref] from piperidine nucleosides[17].

Herein we present the synthesis of the most relevant piperidine nucleotides in respect to RelA. The whole series of piperidine nucleotides will be published separately elsewhere.

4-(Guanin-9-yl)piperidin-1-*N*-ylcarbonylphosphonic acid DR-M014, DR-6011A

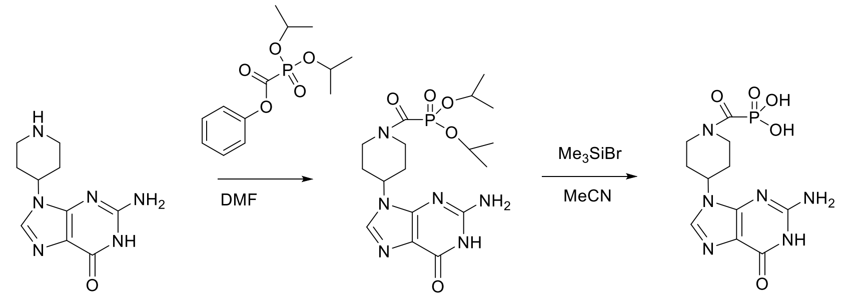

Scheme 15

Diisopropyl phenylphosphonoformiate (0.59 g, 2 mmol) was added to the suspension of 9-(piperidin-4-yl)guanine (0.4 g, 1.71 mmol) in DMF (20 ml). The reaction mixture was stirred at 90 °C overnight and concentrated under reduced pressure. Diisopropyl intermediate was obtained by column chromatography on silica gel using linear gradient of ethanol in chloroform in 55% yield (0.4 g, 0.94 mmol). The intermediate (characterized by means of LCMS only) was co-evaporated with MeCN (2x 10 ml), dissolved in MeCN (10 ml) and Me_3SiBr (0.5 ml, 3.75 mmol) was added under argon atmosphere. The reaction mixture was stirring under argon atmosphere at rt overnight, concentrated in vacuo, quenched

with 2M TEAB (2 ml) and EtOH (10 ml), evaporated, and the final product was obtained by preparative HPLC on reversed phase using linear gradient of MeOH in 0.1M aq. TEAB affording, after converting to ammonium salt by passing through a column of Dowex 50 in Na^+ form, title product in 25% overall yield (85 mg, 0,23 mmol) in the form of white amorphous solid.

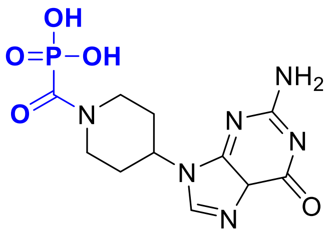

^{1}H NMR (600.1 MHz, D_2O, ref(dioxane) = 3.75 ppm): 1.91 (qd, 1H, J_{gem} = $J_{3= xane)}$ = $J_{3= xane}$ = 12.1, $J_{3= 12.1,}$ = 4.3, H-33.75 ppm): 1.91 (qd,J_{gem} = $J_{5= 4.3, H}$ = $J_{5= 4.3,}$ = 12.1, $J_{5= 12.1,}$ = 4.3, H-5′ax); 2.12 – 2.24 (m, 2H, H-2′eq,5′eq); 2.86 (ddd, 1H, J_{gem} = 13.8, $J_{2= 13.8,}$ = 12.1, $J_{2= 12.1,}$ = 2.7, H-2ddd, 1H, 2H, H-2qd, J_{gem} = 13.8, $J_{6= 13.8,}$ = 12.1, $J_{6= 12.1,}$ = 2.7, H-6ddd, 1H, 2H, H-2qd,$J_{4= 2.7,}$ = $J_{4= 2.7,}$ = 12.0, $J_{4= 12.0}$ = $J_{4= 12.0}$ = 4.1, H-4ddd, 1H, 2H, H-2qJ_{gem} = 13.8, H-2dd, 1H, 2H, H-2qd,H-613.8); 7.88 (s, 1H, H-8).

^{13}C NMR (150.9 MHz, D_2O, ref(dioxane) = 69.3 ppm): 33.87 (CH_2-3oxa 34.58 (CH_2-54.58 (CH 69.3 $J_{C,P}$ = 3.5, CH_2-2 3. 48.33 (CH_2-68.33 (CH 69.3 ppm): 33.87 (CH (CH(CH, H, 85 mg, 0,23 mmol)y passing through a column of Dowex 50 in $J_{C,P}$ = 201.6, COP).

^{31}P{^{1}H} NMR (202.3 MHz, D_2O): -1.94.

IR ν_{max}(KBr) 3426 (s, br), 3130 (s, br), 3030 (s, br, sh), 2948 (m), 2841 (m), 1687 (vs), 1630 (s), 1598 (vs), 1533 (m), 1478 (m), 1443 (m), 1403 (m), 1368 (m), 1314 (w), 1173 (m), 1075 (s), 918 (m), 782 (w), 673 (w), 641 (m).

HRMS (ESI+) for $C_{11}H_{16}N_6O_5P$ $(M+H)^+$: calcd 343.09143, found 343.09154.

4-(Adenin-9-yl)piperidin-1-*N*-ylcarbonylphosphonic acid DR-5994A

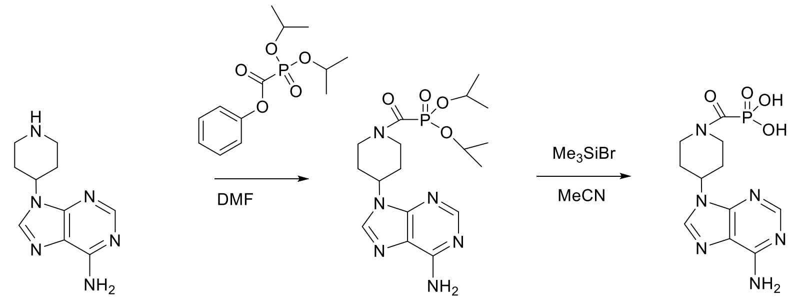

Scheme 16

Diisopropyl phenylphosphonoformiate (0.17 g, 0.6 mmol) was added to the suspension of 9-(piperidin-4-yl)adenine (0.4 g, 1.71 mmol) in DMF (5 ml). The reaction mixture was stirred at 90 °C overnight and concentrated under reduced pressure. Diisopropyl intermediate was obtained by column chromatography on silica gel using linear gradient of ethanol in chloroform in 73% yield (0.18 g, 0.44

mmol). The intermediate (characterized by means of LCMS only) was co-evaporated with MeCN (2x 10 ml), dissolved in MeCN (10 ml) and Me_3SiBr (0.29 ml, 2.19 mmol) was added under argon atmosphere. The reaction mixture was stirring under argon atmosphere at rt overnight, concentrated in vacuo, quenched with 2M TEAB (2 ml) and EtOH (10 ml), evaporated, and the final product was obtained by preparative HPLC on reversed phase using linear gradient of MeOH in 0.1M aq. TEAB affording, after converting to ammonium salt by passing through a column of Dowex 50 in Na^+ form, title product in 53% overall yield (86 mg, 0,23 mmol) in the form of white amorphous solid.

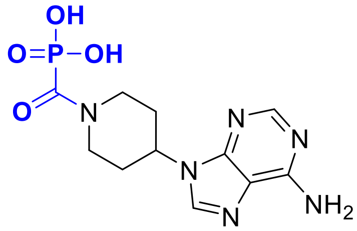

^{1}H NMR (500.0 MHz, D_2O, ref(dioxane) = 3.75 ppm): 1.97 (qd, 1H, $J_{gem} = J_{3'ax,2'ax} = J_{3'ax,4'} = 12.5$, $J_{3'ax,2'eq} = 4.4$, H-3'ax); 2.13 (qd, 1H, $J_{gem} = J_{5'ax,4'} = J_{5'ax,6'ax} = 12.5$, $J_{5'ax,6'eq} = 4.4$, H-5'ax); 2.23 (m, 1H, H-3'eq); 2.26 (m, 1H, H-5'eq); 2.92 (m, 1H, H-2'ax); 3.40 (m, 1H, H-6'ax); 4.63 (m, 1H, H-2'eq); 4.68 (tt, 1H, $J_{4',3'ax} = J_{4',5'ax} = 12.5$, $J_{4',3'eq} = J_{4',5'eq} = 4.4$, H-4'); 4.87 (ddt, 1H, $J_{gem} = 14.0$, $J_{6'eq,5'} =$ 4.4, 2.5, $J_{6'eq,2'eq} = 2.5$, H-6'eq); 8.14 (s, 1H, H-2); 8.19 (s, 1H, H-8).

^{13}C NMR (125.7 MHz, D_2O, ref(dioxane) = 69.3 ppm): 33.85 (CH_2-3'); 34.50 (CH_2-5'); 43.41 (d, $J_{C,P} = 4.0$, CH_2-2'); 48.28 (CH_2-6'); 55.20 (CH-4'); 121.16 (C-5); 142.78 (CH-8); 151.06 (C-4); 154.51 (CH-2); 157.90 (C-6); 176.85 (d, $J_{C,P} = 204.2$, COP).

^{31}P{^{1}H} NMR (202.3 MHz, D_2O): -1.29

IR ν_{max}(KBr) 3433 (vs, br), 3204 (m, vbr), 2931 (w), 2866 (w), 2340 (vw, vbr), 1642 (s), 1594 (s), 1577 (s), 1475 (w), 1450 (w), 1415 (w), 1370 (w), 1329 (w), 1303 (w), 1162 (w), 1105 (m, sh), 1089 (m, br), 995 (w), 912 (w, br), 799 (w), 724 (w), 650 (w).

HRMS (ESI-) for $C_{11}H_{14}N_6O_4P$ $(M-H)^-$: calcd 325.08196, found 325.08148.

Pyrrolidine phosphonates (entries 32-53)
Pyrrolidine phosphonates were prepared by procedures already published: [11, 14-16, 18-20].

Prolinol phosphonates (entries 54-57)
The detailed description of the synthesis will be published separately elsewhere.

Acyclic phosphonates (entries 58-60)

(2-(guanin-9-yl)ethyl)carbamoylphosphonic acid DR-5163

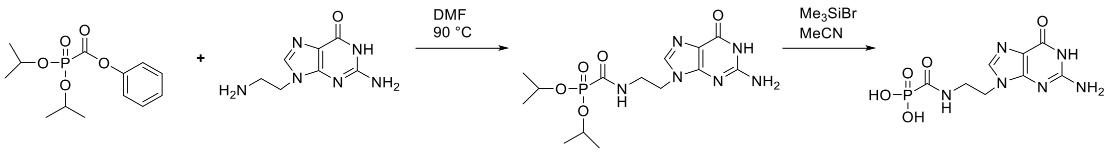

Scheme 17

Diisopropyl phenylphosphonoformiate (0.39 g, 1.36 mmol) was added to the suspension of 9-(2-aminoethyl)guanine (0.24 g, 1.24 mmol) in DMF (15 ml). The reaction mixture was stirred at 90 °C overnight and concentrated under reduced pressure. Diisopropyl intermediate was obtained by column chromatography on silica gel using linear gradient of ethanol in chloroform in 60% yield (0.29 g, 0.74 mmol). The intermediate (characterized by means of LCMS only) was co-evaporated with MeCN (2x 10 ml), dissolved in MeCN (10 ml) and Me_3SiBr (0.49 ml, 3.7 mmol) was added under argon atmosphere. The reaction mixture was stirring under argon atmosphere at rt overnight, concentrated in vacuo, quenched with 2M TEAB (2 ml) and EtOH (10 ml), evaporated, and the final product was obtained by preparative HPLC on reversed phase using linear gradient of MeOH in 0.1M aq. TEAB affording, after converting to ammonium salt by passing through a column of Dowex 50 in Na^+ form, title product in 47% yield (120 mg, 0,35 mmol) in the form of white amorphous solid.

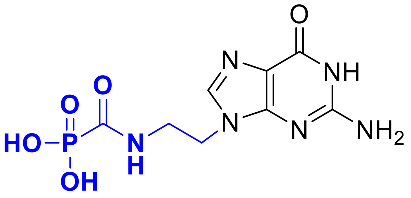

^{1}H NMR (500.0 MHz, D_2O, ref(dioxane) = 3.75 ppm): 3.64 (m, 2H, NHC$\mathbf{H_2}$CH$_2$N); 4.23 (m, 2H, NHCH$_2$C$\mathbf{H_2}$N); 7.78 (s, 1H, H-8).

^{13}C NMR (125.7 MHz, D_2O, ref(dioxane) = 69.3 ppm): 41.06 (d, $J_{C,P}$ = 6.6, NH**C**H$_2$CH$_2$N); 45.73 (NHCH$_2$**C**H$_2$N); 118.68 (C-5); 142.97 (CH-8); 154.47 (C-4); 156.49 (C-2); 161.72 (C-6); 179.33 (d, $J_{C,P}$ = 200.0, COP).

^{31}P NMR (202.3 MHz, D_2O): -2.24.

IR ν_{max}(KBr) 3433 (vs, br), 3351 (s), 3207 (m), 3153 (w), 3037 (w), 2944 (vw), 2758 (w, br), 2340 (w, br), 1707 (s, sh), 1687 (s), 1635 (vs), 1617 (vs), 1566 (m, sh), 1540 (m), 1523 (m), 1489 (w), 1447 (w), 1438 (w), 1413 (w), 1389 (w), 1369 (w), 1230 (w, br), 1069 (m), 923 (w), 784 (w), 774 (w), 682 (w), 639 (m)

HRMS (ESI+) for $C_8H_{11}NO_5NaP$ $(M+Na)^+$: calcd 325.04208, found 325.04211.

Azetidine phosphonates (entries 61-64)

The detailed description of the synthesis will be published separately elsewhere.

Naturally occurring nucleotides (entries 65-70) were purchased from Sigma-Aldrich

Supplementary References:

1. Mechold, U., Murphy, H., Brown, L. & Cashel, M. Intramolecular regulation of the opposing (p)ppGpp catalytic activities of Rel(Seq), the Rel/Spo enzyme from Streptococcus equisimilis. *J Bacteriol* **184**, 2878-88 (2002).
2. Shyp, V. et al. Positive allosteric feedback regulation of the stringent response enzyme RelA by its product. *EMBO Rep* **13**, 835-9 (2012).
3. Sebaugh, J.L. Guidelines for accurate EC50/IC50 estimation. *Pharm Stat* **10**, 128-34 (2011).
4. Bernardo, L.M., Johansson, L.U., Solera, D., Skarfstad, E. & Shingler, V. The guanosine tetraphosphate (ppGpp) alarmone, DksA and promoter affinity for RNA polymerase in regulation of sigma-dependent transcription. *Mol Microbiol* **60**, 749-64 (2006).
5. Nanamiya, H. et al. Identification and functional analysis of novel (p)ppGpp synthetase genes in Bacillus subtilis. *Mol Microbiol* **67**, 291-304 (2008).
6. Vasantha, N. & Freese, E. Enzyme changes during Bacillus subtilis sporulation caused by deprivation of guanine nucleotides. *J Bacteriol* **144**, 1119-25 (1980).
7. Cutting, S.M. & Horn, P.B.V. in Modern Microbiological Methods (eds. Harwood, C.R. & Cutting, S.M.) (John Wiley & Sons, Inc., New York, 1990).
8. Cutting, S.M. & Horn, P.B.V. in Molecular biological methods for Bacillus (eds. Hardwood, C.R. & Cutting, S.M.) (Cutting, New York, 1990).
9. Schattenkerk, C., Wreesmann, C.T.J., van der Marel, G.A. & van Boom, J.H. Synthesis of riboguanosine pentaphosphate ppprGpp (Magic Spot II) via a phosphotriester approach. *Nucleic Acids Research* **13**, 3635-3649 (1985).
10. Gaca, A.O. et al. From (p)ppGpp to (pp)pGpp: Characterization of Regulatory Effects of pGpp Synthesized by the Small Alarmone Synthetase of Enterococcus faecalis. *J Bacteriol* (2015).
11. Keough, D.T. et al. Inhibition of the Escherichia coli 6-Oxopurine Phosphoribosyltransferases by Nucleoside Phosphonates: Potential for New Antibacterial Agents. *Journal of Medicinal Chemistry* **56**, 6967-6984 (2013).
12. Kocalka, P. et al. in Chemistry of Nucleic Acid Components 415-416 (2005).
13. Pohl, R. et al. in Chemistry of Nucleic Acid Components 435-436 (2008).
14. Slavetinska, L.P., Rejman, D. & Pohl, R. Pyrrolidine nucleotide analogs with a tunable conformation. *Beilstein Journal of Organic Chemistry* **10**, 1967-1980 (2014).
15. Rejman, D., Pohl, R., Kocalka, P., Masojidkova, M. & Rosenberg, I. Pyrrolidine N-alkylphosphonates and related nucleotide analogues: synthesis and stereochemistry. *Tetrahedron* **65**, 3673-3681 (2009).
16. Rejman, D. et al. N-Phosphonocarbonylpyrrolidine Derivatives of Guanine: A New Class of Bi-Substrate Inhibitors of Human Purine Nucleoside Phosphorylase. *Journal of Medicinal Chemistry* **55**, 1612-1621 (2012).
17. Kovackova, S., Dracinsky, M. & Rejman, D. The synthesis of piperidine nucleoside analogs-a comparison of several methods to access the introduction of nucleobases. *Tetrahedron* **67**, 1485-1500 (2011).
18. Kocalka, P., Pohl, R., Rejman, D. & Rosenberg, I. Synthesis of racemic and enantiomeric 3-pyrrolidinyl derivatives of nucleobases. *Tetrahedron* **62**, 5763-5774 (2006).

19. Rejman, D., Kocalka, P., Budesinsky, M., Pohl, R. & Rosenberg, I. Synthesis of diastereomeric 3-hydroxy-4-pyrrolidinyl derivatives of nucleobases. *Tetrahedron* **63**, 1243-1253 (2007).
20. Pohl, R. et al. Synthesis, conformational studies, and biological properties of phosphonomethoxyethyl derivatives of nucleobases with a locked conformation via a pyrrolidine ring. *Org Biomol Chem* **13**, 4693-705 (2015).
